# Unambiguous Spectroscopic Characterization of a Gold Difluorocarbene

**DOI:** 10.1021/jacsau.5c00911

**Published:** 2025-09-04

**Authors:** Miquel Navarro, Louise Delaurenti, Alejandra Pita-Milleiro, Jesús Campos

**Affiliations:** Instituto de Investigaciones Químicas (IIQ), Departamento de Química Inorgánica and Centro de Innovación en Química Avanzada (ORFEO−CINQA), 518805 Consejo Superior de Investigaciones Científicas (CSIC) and University of Sevilla, Seville 41092, Spain

**Keywords:** carbene, alkylidene, gold(I), bulky phosphine, fluorine, ligand cavity

## Abstract

Gold­(I) carbenes, defined as [LAu=CR_2_]^+^, are key intermediates in catalysis. Their isolation is challenging due to the high electrophilic character of the carbene that cannot be tempered solely by π-backdonation from gold. Among those, the simple difluorocarbene complex has been invoked in several studies, but attempts to isolate it have failed, while its identification remains questionable. In this study, we report the unambiguous spectroscopic characterization of the gold difluorocarbene complex [P–Au=CF_2_]^+^, providing key insights into its structural and electronic features. The kinetic stabilization of the carbene was only possible owing to the shielding offered by a congested and cavity-shaped phosphine that prevents decomposition under strict anhydrous conditions. Besides, the nature of the anion is key to provide further thermal stability for prolonged times.

## Graphical abstract

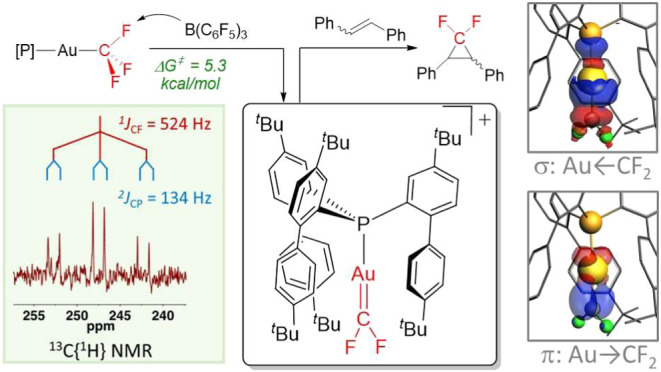

Gold catalysis has become a powerful tool for the synthesis of complex organic compounds, and the number of novel gold-mediated reactions has increased exponentially.^1^ However, the isolation and characterization of different important classes of gold compounds, such as π complexes,^2,3^ hydrides,^4^ hydroxides,^5^ carbonyls,^6^ and carbenoids,^7^ that are considered catalytically active intermediates in these transformations, have advanced at a much slower pace. In this regard, isolation of cationic nonstabilized carbene gold complexes, [LAu=CR_2_]^+^ (where R stands for H, alkyl, or aryl) has gathered increasing attention over the last years due to their involvement in a wide range of catalytic transformations.^8^

This type of species is intrinsically unstable in the absence of heteroatoms bound to the carbenic carbon. In those cases, the limited ability of [LAu]^+^ fragments for π-backdonation to the empty carbene p-orbital renders the carbene too electrophilic for efficient stabilization, though highly reactive and thereby relevant to catalysis.^8^ Nevertheless, different strategies have succeeded in stabilizing, isolating, and even structurally characterizing some examples (Figure 1). For instance, Fürstner and Widenhoefer described the isolation of two cationic gold carbenes stabilized by conjugation with appropriately substituted arenes ([Cy_3_PAuCAr_2_]^+^ (Ar = p-MeOC_6_H_4_^–^), **A**)^9^ or within the aromatic ring in the gold cycloheptatrienylidene complex **B**.^10^ Also, Straub has reported the isolation of a cationic gold carbene via extreme steric shielding conferred by the IPr** ancillary ligand (**C**).^11^ On the other hand, Bourissou has capitalized on the use of bidentate (o-carboranyl)-diphosphines to describe a family of tridentate gold­(I) cationic carbene species (**D**), in which enhanced π-backdonation (owing to P∧P chelation) from gold to the carbene fragment stabilizes the corresponding gold–carbene bond.^12^

**Figure 1:**
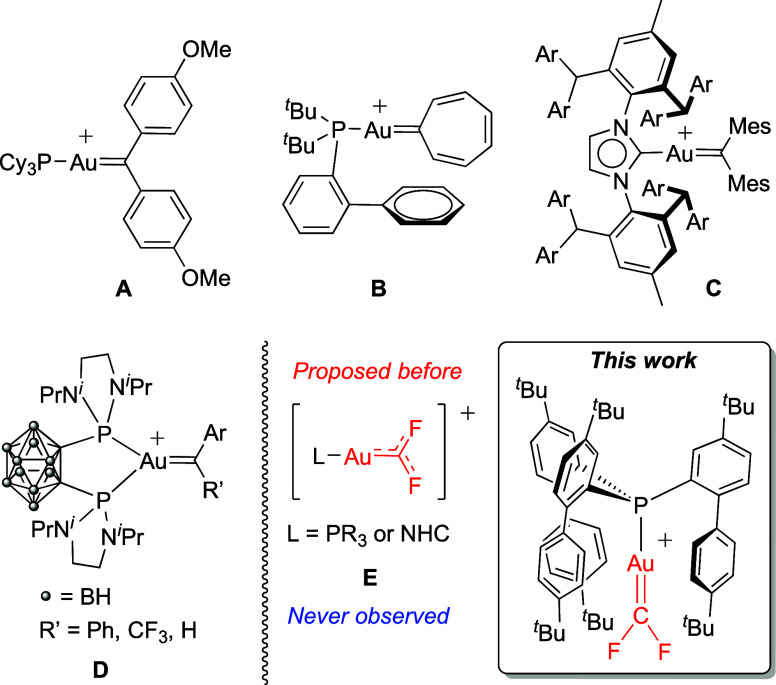
Representative examples of structurally characterized cationic gold­(I) carbene complexes (A–D), schematic representation of often postulated but never identified gold difluorocarbene complexes E stabilized by π-donation from the fluorine lone pairs, and structure of the first unambiguously characterized gold-difluorocarbene complex (this work).

Despite these advances, there is still an unmet synthetic challenge, namely, a gold-bound difluorocarbene (:CF_2_). This is a particularly appealing goal, even more considering that Toste et al. proposed a F-rebound mechanism in C­(sp^3^)–CF_3_ coupling involving gold­(III) difluorocarbenes as key intermediates.^13^ However, despite being proposed in several works,^14^ the identification of such a fleeting species remains a gap in the organometallic chemistry of gold. In this line, Fürstner suggested that gold­(I) difluorocarbenes (**E**) are likely at the edge of being observable.^15^ In principle, the strongly electron-withdrawing σ-character of the fluorine substituents might be partly compensated by some degree of F-π-donation (Figure 1).^15^ However, only gold difluorocarbenoid complexes with retained coordinated anions, such as triflate or triflimide, have been characterized, though they exhibited spectroscopic data opposing the definition of a true carbene. More recently, Nolan and co-workers tentatively suggested the formation of a transient NHC-supported gold­(I) difluorocarbene, by indirect means although it could not be spectroscopically identified due to its fleeting nature.^16^ Although a broad ^19^F NMR signal at 247 ppm was suggested to result from a CF_2_ moiety, there was no ^13^C NMR signal with appropriate multiplicity and chemical shift fitting a postulated difluorocarbene fragment, therefore lacking spectroscopic identification.

Our group has recently leveraged the cavity-shaped ligand tris-2-(4,4′-di*tert*-butylbiphenylyl)­phosphine (**L1**) to access otherwise unstable gold­(I) adducts,^17^ including the first dicoordinate gold­(I) ethylene^18^ and acetylene complexes.^19^ The pocket offered by this ligand proved crucial for the kinetic stabilization of these species, and therefore, we envisioned it as an ideal candidate to accommodate a difluorocarbene fragment. Accordingly, we describe, for the first time, the unequivocal spectroscopic identification of a gold­(I) difluorocarbene complex.^200^ Besides, we have explored its reactivity and analyzed its bonding by EDA-NOCV methods.

A convenient entry into a potential gold difluorocarbene involves fluoride abstraction from a parent trifluoromethyl gold compound,^15,16^ which in turn can be prepared from the corresponding Au-fluoride by treatment with TMSCF_3_.^20^ Therefore, we first examined the reaction of complex (**L1**)­AuCl (**1**) with an excess of AgF in dichloromethane to generate the desired fluoride gold complex. However, no conversion was observed even after heating the reaction for several hours, which is consistent with literature reports.^21b^ In view of these findings, we used the approached disclosed by Kaupp, Braun and co-workers consisting in substituting the chloride for a more reactive Au–iodide bond. With this aim, we performed the reaction between complex **1** and an excess of NaI in a mixture of CH_2_Cl_2_/MeOH to generate the respective air-stable iodo complex **2** in excellent yield (89%, Scheme 1). ^31^P­{^1^H} NMR spectroscopy revealed full conversion within 30 min with the appearance of a new signal at 18.6 ppm (c.f. 9.5 ppm for complex **1**), though very few changes were apparent by ^1^H NMR. The complex was confirmed by X-ray diffraction analysis (see Figure S24)

**Scheme 1:**
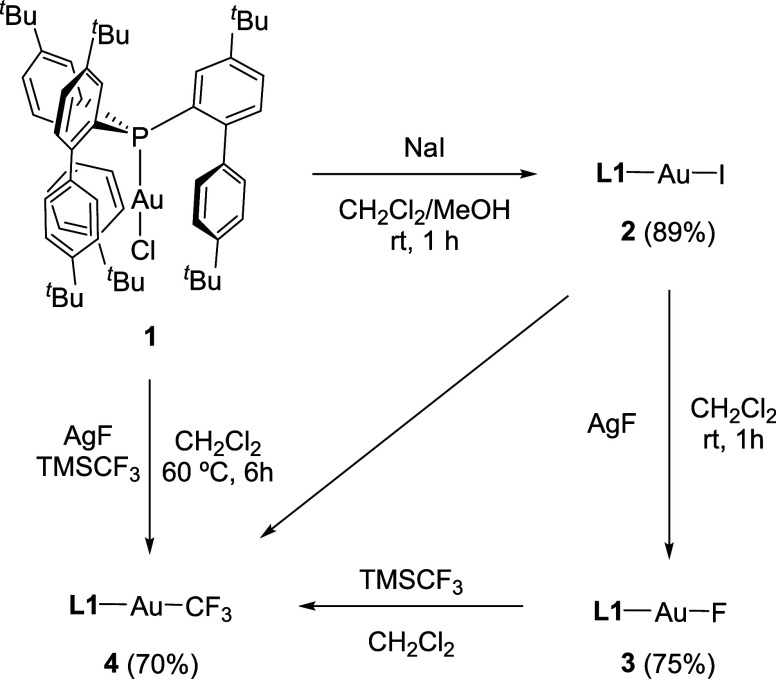
Synthesis of Gold Complexes **2**, **3**, and **4**

Treatment of complex **2** with an excess of AgF in dichloromethane under sonication for 1 h afforded the desired gold fluoride complex **3** (Scheme 1) in good yields (75%). The ^31^P­{^1^H} and ^19^F­{^1^H} NMR spectra revealed characteristic doublets (^2^ *J*_PF_ = 147 Hz) at 1.7 and –222.5 ppm, respectively. Complex **3** showed remarkable stability both in solution and in the solid state under nitrogen atmosphere; however, it slowly decomposes through hydrolysis in the presence of air and moisture, generating the previously reported [**L1**–Au–OH] complex^19^ and HF. Additionally, the linear structure of **3** was corroborated by X-ray diffraction analysis (Figure 2).

**Figure 2:**
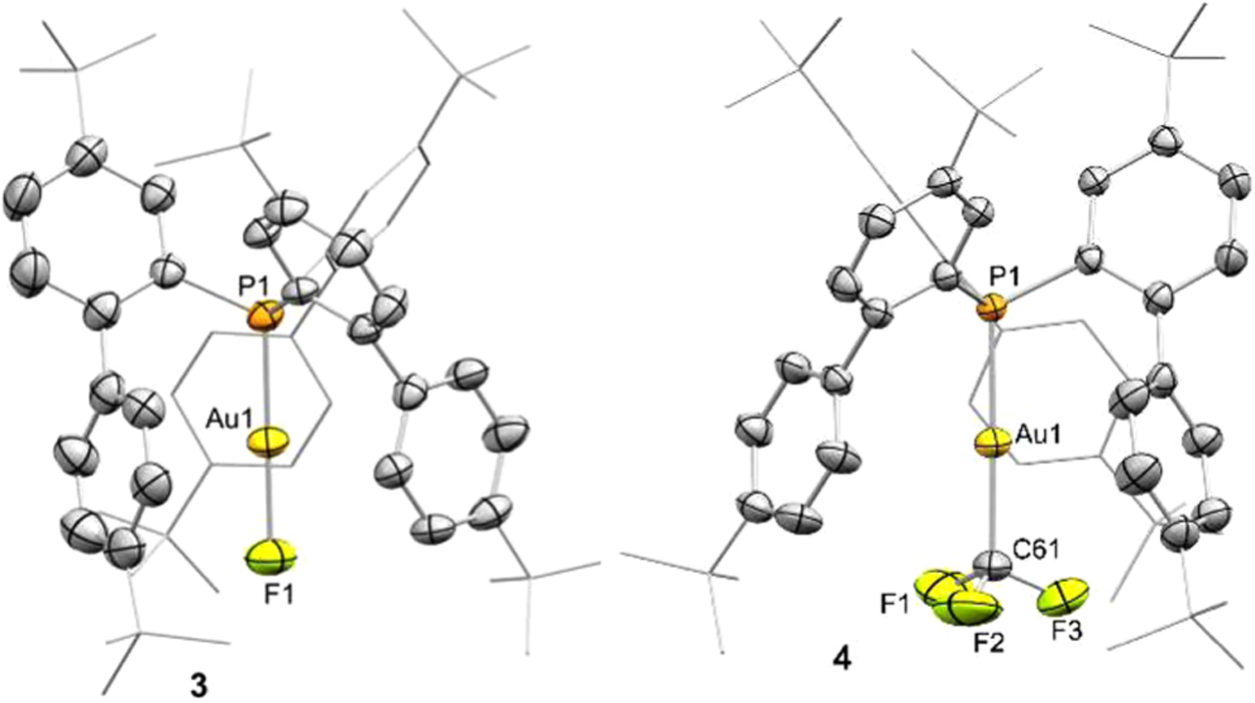
ORTEP diagram of compounds **3** and **4**. Solvent molecules (one dichloromethane and one toluene molecule for **3** and **4**, respectively) and hydrogen atoms are excluded for clarity, while *tert*-butyl groups and one biaryl fragment are represented in wireframe format. Thermal ellipsoids are set at 50% probability. Selected bond distances (Å) and angles (°): **3**, Au1–P1, 2.200(2); Au1–F1, 2.013(5); P1–Au1–F1, 179.4(1); **4**, Au1–P1, 2.2878(7), Au1–C61, 2.085(3); P1–Au1–C61, 179.3(1).

Next, we focused our efforts on the preparation of the target gold­(I) trifluoromethyl complex **4**, for which different synthetic pathways were explored (Scheme 1). First, the reaction of the fluorido complex **3** with trimethyltrifluoromethylsilane (TMSCF_3_) in dichloromethane was monitored by ^31^P­{^1^H} NMR spectroscopy, resulting in the appearance of a new quadruplet signal at 22.2 ppm (^3^ *J*_PF_ = 41 Hz), indicative of the formation of complex **4**. However, conversions were very low, even at long reaction times and high temperatures. Optimization of the reaction conditions led us to explore one-pot reactions starting either from precursors **1** or **2** in the presence of an excess of TMSCF_3_ and AgF. While using complex **2** resulted in the formation of complex **4** in low conversion, along with some other side products, the use of **1** as precursor afforded exclusively complex **4** with complete conversion after 6 h. The trifluoromethyl species **4** presents remarkable stability in open air conditions and was purified by column chromatography in order to remove excess TMSCF_3_, the resulting silanes, and silver salts. The structure of complex **4** was authenticated as well by a single-crystal X-ray diffraction analysis (Figure 2).

With compound **4** in hand, we explored the reactivity with different Lewis acids to mediate an α-fluoride elimination toward the target difluorocarbene. The reaction of **4** with BF_3_·Et_2_O or [Ph_3_C]­[BF_4_] was monitored by ^31^P­{^1^H}­NMR spectroscopy at −80 °C. This revealed the fast disappearance of the quadruplet at 22.2 ppm corresponding to complex **4** and the appearance of a new singlet at 6.7 ppm, which was identified as the previously described [**L1**–Au–CO]^+^ complex.^18a^ The generation of the carbonyl adduct is consistent with the hydrolysis of a potential gold­(I) difluorocarbene due to traces of water.^14c,22^ To minimize its presence, we reacted complex **4** with a freshly sublimated sample of B­(C_6_F_5_)_3_ at −80 °C in dichloromethane, which provoked a drastic color change of the solution from colorless to bright orange, suggesting the formation of the aimed gold­(I) difluorocarbene complex **5** (Figure 3a). ^31^P­{^1^H} NMR monitoring revealed complete consumption of **4** (quartet at 22.2 ppm, ^3^ *J*_PF_ = 41 Hz) and full conversion to complex **5** identified with the appearance of a new triplet at 9.6 ppm with ^3^ *J*_PF_ = 28 Hz (Figure 3b), notably smaller compared to **4**. In addition, a new doublet was observed in the ^19^F­{^1^H} NMR spectrum at 146.9 ppm with the same coupling constant (c.f. −27.2 ppm and ^3^ *J*_PF_ = 41 Hz for precursor **4**). This chemical shift is slightly downfield shifted in comparison with other metal-difluorocarbene species from other late transition metals;^23^ however, together with its coupling constant, it is in full agreement with the planar sp^2^ character of the CF_2_ moiety that makes two fluorine atoms chemically equivalent. Compound **5** showed remarkable stability in dichloromethane solution, though only at low temperature (<−50 °C), which allowed us to unambiguously characterize it in situ by ^1^H, ^19^F­{^1^H}, ^31^P­{^1^H} and ^13^C­{^1^H} NMR spectroscopy. It exhibits a distinctive carbenic carbon signal similar to reported difluorocarbenes of other transition metals^23^ at 247.1 ppm as a triplet of doublets with coupling constants of ^1^ *J*_CF_ = 524 Hz and ^2^ *J*_CP_ = 134 Hz (Figure 3b; c.f. ^1^ *J*_CF_ = 356 Hz and ^2^ *J*_CP_ = 180 Hz for precursor **4**), which evince the genuine carbenic nature of the CF_2_ fragment.

**Figure 3:**
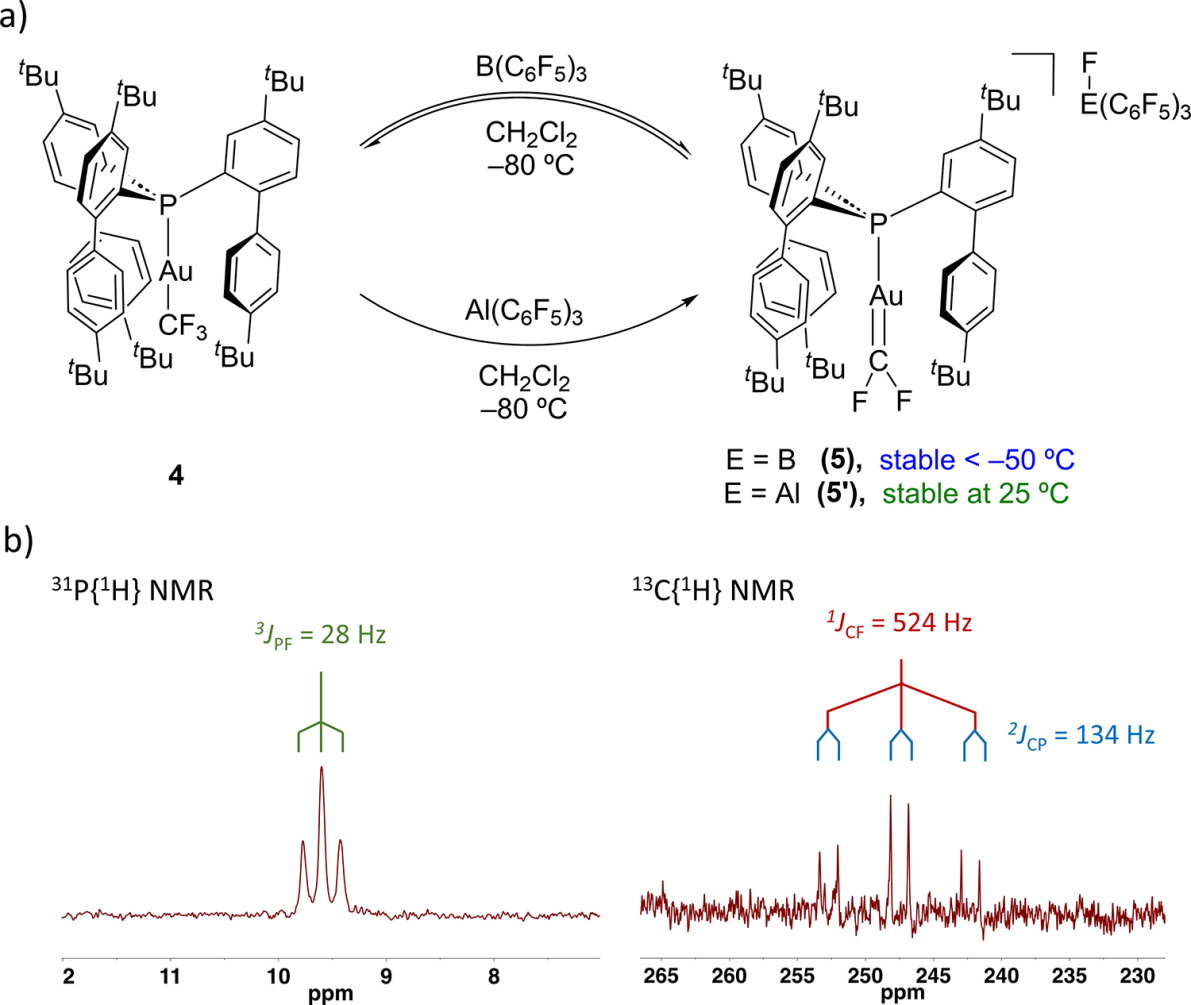
Synthesis of gold difluorocarbene complex **5** or **5′** by α-fluoride abstraction with B­(C_6_F_5_)_3_ or Al­(C_6_F_5_)_3_, respectively; (b) ^31^P­{^1^H} and ^13^C­{^1^H} (carbenic region) NMR spectra of complex **5**.

Upon warming up the solution above −50 °C, slow decomposition of **5** was observed, generating the aforementioned hydrolyzed [**L1**–Au–CO]^+^ carbonyl complex. We carried out many attempts to grow single crystals suitable for X-ray diffraction analysis, however, in all cases, precursor **4** crystallized even from samples in which its apparent full consumption was recorded by NMR spectroscopy. Although it seemed surprising, this behavior is consistent with our DFT-computed reaction profile for α-fluoride abstraction. This revealed an exergonic single-step elimination process with a small 5.3 kcal/mol barrier that perfectly fits the spontaneous formation of **5** at low temperature (Figure S25). However, the process is almost thermoneutral (Δ*G°* = −1.2 kcal/mol), with the reverse reaction presenting only a 6.0 kcal/mol barrier, indicating reversibility to the overall process and enabling the crystallization of precursor **4**.

Interestingly, the use of Al­(C_6_F_5_)_3_ instead of B­(C_6_F_5_)_3_ as the Lewis acid to mediate the α-fluoride elimination also resulted in the formation of the aluminate derivative of the gold­(I) difluoro-carbene complex, **5′** (Figure 3a). The analogous nature of the gold difluorocarbene was determined by virtually identical ^1^H and ^31^P­{^1^H} NMR spectra (Figures S18 and S19). The formation of the resulting aluminate [Al­(F)­(C_6_F_5_)_3_]-anion was ascertained by the appearance of slightly shifted ^19^F­{^1^H} NMR resonances compared to the precursor at –122.8, –154.9, and –163.6 ppm, along with a new weaker signal at –162.1 ppm due to the Al–F termini (Figure S20), in accordance with prior reports.^24^ Nonetheless, the most notable feature resulting from an apparent innocent substitution of the counteranion is the remarkably higher thermal stability of the gold-difluorocarbene (**5′**), which remained stable for hours even at 25 °C (Figure S23). Besides, the aforesaid dynamic reversibility for the α-fluoride elimination reaction that accounts for the equilibrium between **4** and **5** is not evident in the case of the heavier aluminate (**5′**). Our computational investigations reveal that at variance with the thermoneutral reaction with the borane (ΔG° = –1.2 kcal/mol), the reaction with the alane is exergonic (ΔG° = –11.5 kcal/mol) due to the higher fluoride anion affinity (FIA) of the latter (Figure S25 and S26).^25^ This is also consistent with our experimental observations, as the variable temperature ^31^P­{^1^H} NMR of a sample containing **4** and **5** reveals dynamic exchange at temperatures above 253K (Figure S21), whereas no apparent equilibrium was discerned for **4** and **5′** even at 298K (Figure S22).

The reactivity of complex **5** was explored to further corroborate its carbene character. While complex **4** showed no reactivity in the presence of *E*- or *Z*-stilbene, addition of B­(C_6_F_5_)_3_ at −50 °C afforded a mixture of difluorocyclopropane (**6** and **6′**) and difluoro-olefin **7** in moderate yields of ∼30% after 18 h (Scheme 2). The reactivity is the same as that found by Fürstner for his reported base-stabilized carbenoids, which corroborate the proposed formulation for **5**, as well as the masked carbenic character of the aforesaid prior work. As for the resulting gold complex, the main species resulting from these tests was the carbonyl adduct [**L1**–Au–CO]^+^ due to adventitious water, as well as other unidentified species in minor amounts.

**Scheme 2:**
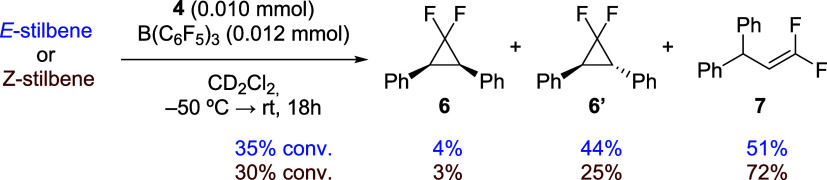
Reactivity of In Situ Generated Gold Difluorocarbene **5** with *E*- and *Z*-Stilbene

Finally, we explored the bonding in the difluorocarbene complex **5** by Energy Decomposition Analysis-Natural Orbital for Chemical Valence (EDA-NOCV) method (Table 1 and Figure 4) at the ZORA-BP86-D3/TZ2P//BP86-D3/def2-SVP level of theory (see Section 4.1 of the SI), as well as in complexes **3** and **4** for comparison. In all cases, the main contribution to the attractive interaction energy between the metal fragment [**L1**–Au]^+^ and the fluorinated moieties comes from the electrostatic attractions (**3**, 65%; **4**, 74%; and **5**, 66%). The main orbital interaction within the total Δ*E*_orb_ term is the σ-donation from the doubly occupied p­(F), p­(CF_3_), or p­(CF_2_) orbital to the vacant σ*­(Au–P) orbital (denoted as Δ*E*(ρ1) in Table 1 and Figure 4). Besides, there are considerable π-contributions to the bonding that are highly dissimilar along the series. In **3**, we found two π-donations from two doubly occupied p­(F), each to a π*–type orbital of [**L1**–Au]^+^ (Δ*E*(ρ2) and Δ*E*(ρ3)), which combined are three times weaker than Δ*E*(ρ1). In stark contrast, complexes **4** and **5** exhibit π-backdonation from an occupied dπ­(Au) atomic orbital to empty p­(CF_3_) or p­(CF_2_) orbitals (Δ*E*(ρ2)), although of notably different energy. In complex **4** π-backdonation is remarkably weak (Δ*E*(ρ1) is 8 times stronger than Δ*E*(ρ2)). In contrast, in complex **5** π-backdonation is half as strong as the main σ-donation, evidencing the high electrophilic character of the CF_2_ fragment, in line with its genuine carbenic character.

**Table 1:** EDA-NOCV Data (in kcal/mol) Computed for Complexes **3**, **4**, and **5**

	**3**	**4**	**5**
Δ*E*_int_	–250.7	–397.0	–254.5
Δ*E*_Pauli_	96.5	238.5	199.3
Δ*E*_elstat_ ^a^	–162.6 (64.9%)	–293.6 (74.0%)	–167.1 (65.7%)
Δ*E*_orb_ ^a^	–85.1 (33.9%)	–92.6 (23.3%)	–76.8 (30.2%)
Δ*E*_orb_(ρ1)^b^	–47.4 (55.7%)	–60.7 (65.6%)	–38.4 (50.0%)
Δ*E*_orb_(ρ2)^b^	–7.4 (8.7%)	–7.3 (7.9%)	–19.2 (24.9%)
Δ*E*_orb_(ρ3)	–7.2 (8.5%)	-	-
Δ*E*_orb_(rest)^b^	–23.1 (27.1%)	–24.6 (26.5%)	–19.2 (25.1%)
Δ*E*_disp_ ^a^	–3.0 (1.2%)	–10–8 (2.7%)	–10.5 (4.1%)

a The values within parentheses indicate the percentage of the total attractive interactions, Δ*E*_int_ = Δ*E*_elstat_ + Δ*E*_orb_ + Δ*E*_disp_.

b The values within parentheses indicate the percentage of the total orbital interactions (Δ*E*_orb_).

**Figure 4:**
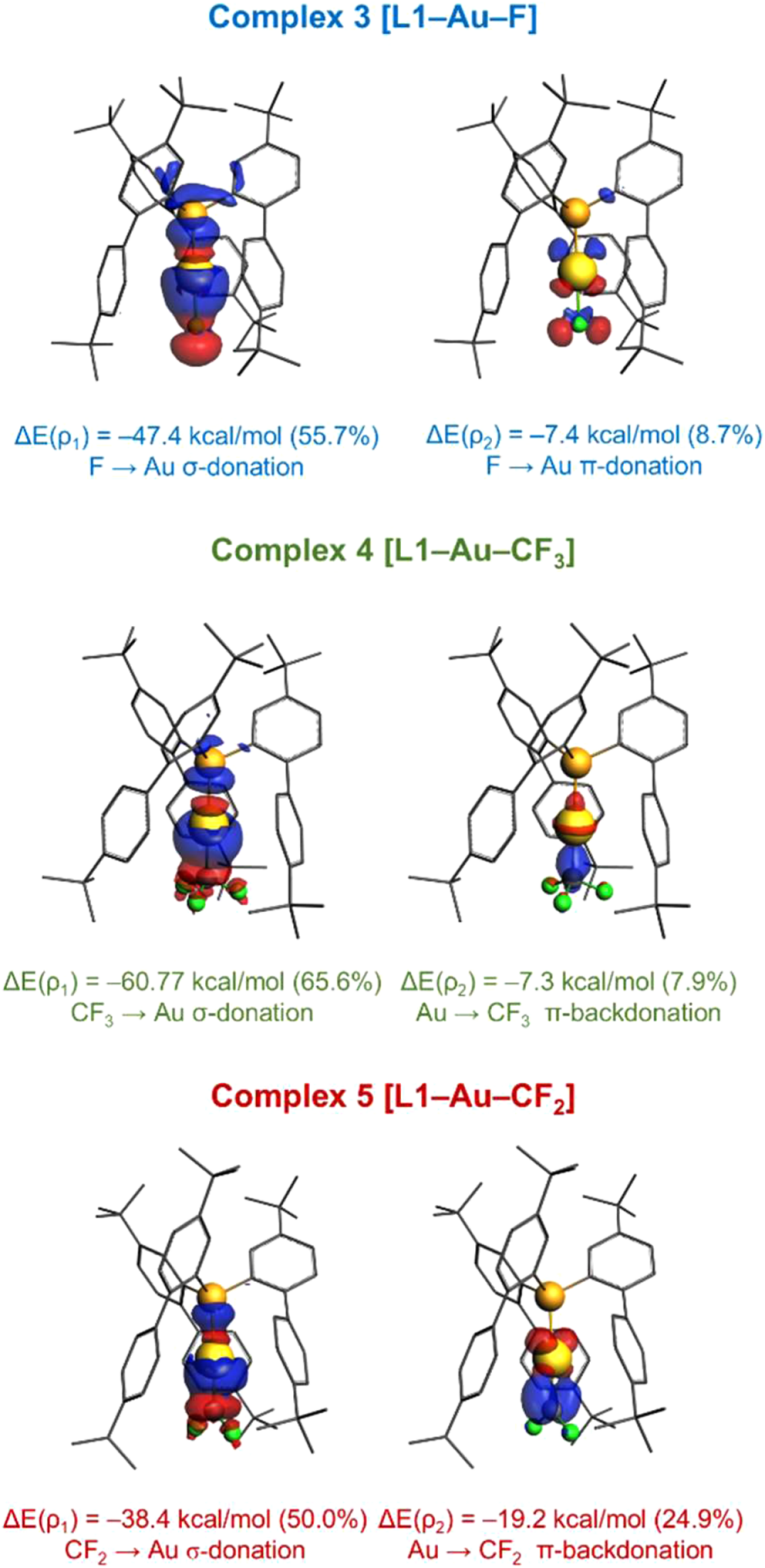
Contour plots of NOCV deformation densities Δρ and associated energies Δ*E*(ρ) in complexes **3**, **4**, and **5**. Electron-density charge flows in the direction red → blue.

In summary, we have spectroscopically characterized, for the first time and unambiguously, a gold difluorocarbene complex. At variance with previous studies, the shielding provided by a cavity-shaped phosphine was instrumental to in situ stabilize such a fleeting species, though attempts to isolate it in pure form proved unsuccessful due to extreme reactivity with adventitious water. The nature of the counteranion was also important to modulate the stability of the carbene, which remains stable even at 25 °C for prolonged times when moving from the more common B­(C_6_F_5_)_3_ to its less frequent heavier version, Al­(C_6_F_5_)_3_. Its carbene-like reactivity was demonstrated by the cyclopropanation reaction with *E*- and *Z*-stilbene. Finally, our computational studies evinced a notable contribution of π-backdonation from the gold fragment into the empty p-orbital of the carbene to compensate its high electrophilicity, around half as strong as the main σ-donation from the CF_2_ fragment to gold.

## Supplementary Material



## Notes

Experimental procedures, NMR spectra, X-ray structural data, and computational details, brief description (PDF)

## †

Departamento de Química Orgánica, Universidad Autónoma de Madrid (UAM) and Institute for Advanced Research in Chemical Sciences (IAdChem), Avda. Francisco Tomás y Valiente 7, Cantoblanco, 28049 Madrid, Spain
