# Supplementary material for: Unambiguous Spectroscopic Characterization of a Gold Difluorocarbene
Source: JACS Au. 2025 Sep 4;5(9):4604–10. doi: 10.1021/jacsau.5c00911 (PMC12458014; doi:10.1021/jacsau.5c00911)
Supplement: Supplementary file 1 [file au5c00911_si_001.pdf]

Supporting Information

Unambiguous Spectroscopic Characterization of a Gold Difluorocarbene

Miquel Navarro,*[a],[b],[c] Louise Delaurenti,[a] Alejandra Pita-Milleiro[a] and Jesús Campos*[a]

[a] Instituto de Investigaciones Químicas (IIQ), Departamento de Química Inorgánica and Centro de Innovación en Química Avanzada (ORFEO−CINQA). Consejo Superior de Investigaciones Científicas (CSIC) and University of Sevilla, 41092 Sevilla, Spain

[b] Organic Chemistry Department, Universidad Autónoma de Madrid (UAM), Avda. Francisco Tomás y Valiente 7, Cantoblanco, 28049 Madrid, Spain

[c] Institute for Advanced Research in Chemical Sciences (IAdChem), Universidad Autónpma de Madrid (UAM), Avda. Francisco Tomás y Valiente 7, Cantoblanco, 28049 Madrid, Spain

E-mail: miquel.navarro@uam.es, jesus.campos@iiq.csic.es

1. Synthetic procedures S2

2. NMR spectroscopic experiments S6

3. Crystal structure determinations S20

4. Computational details S23

5. References S73

1. Synthetic procedures

General considerations. Unless otherwise stated, all reactions and manipulations were carried out under an atmosphere of dry argon or nitrogen using standard Schlenk techniques or in a nitrogen glovebox. Solvents were distilled under inert atmosphere prior to use. Solution ^{1}H, ^{13}C and ^{31}P NMR spectra were recorded on Bruker DRX-400 or DRX-500 spectrometers at 298 K unless otherwise stated. Chemical shifts (δ) are expressed with a positive sign, in parts per million. ^{1}H and ^{13}C chemical shifts reported are referenced internally to residual protio (^{1}H) or deutero (^{13}C) solvent, while ^{11}B shifts are relative to $BF_3{\cdot}OEt_2$, ^{19}F to fluorotrichloromethane and ^{31}P chemical shifts are relative to 85% H_3PO_4. The following abbreviations and their combinations are used: br, broad; s, singlet; d, doublet; t, triplet; m, multiplet. The ^{1}H and ^{13}C resonance signals were attributed by means of 2D HSQC and HMBC experiments. Infrared spectra were recorded on a Bruker Vector 22 spectrometer, and sample preparation was carried out in dry dichloromethane solution. For elemental analyses a LECO TruSpec CHN elementary analyser was utilized. Complex **1** was synthesized as reported in the literature.[1] All other reagents were used as received from commercial suppliers.

Compound 2

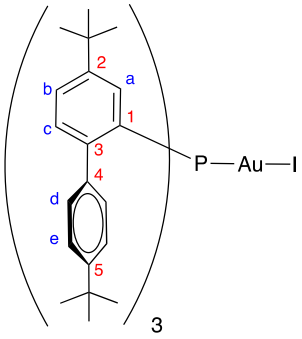

In a round-bottom flask, a suspension of complex **1** (105 mg, 0.10 mmol) and NaI (43 mg, 0.30 mmol) in a 1:1 mixture CH_2Cl_2/MeOH (10 mL) was stirred at rt for 1 h. The solvent was then removed under vacuum, redissolved in CH_2Cl_2 and filtered through a short pad of Celite, yielding complex **2** as a colorless solid (98 mg, 89%). Crystals suitable for X-ray diffraction were grown by slow evaporation of toluene into a dichloromethane solution of complex **2**. **Anal. Calcd.** for $C_{60}H_{75}AuIP$: C, 62.61; H, 6.57. **Found:** C, 62.71; H, 6.31. **^{1}H NMR** (400 MHz, CD_2Cl_2, 25 ºC) δ: 7.59 (d, 3H, $^{3}J_{HH}$ = 8.0 Hz, H_b), 7.36 (t, 2H, $^{3}J_{HH}$ = 11.4 Hz, H_a), 7.32–7.25 (m, 3H, H_c), 7.08 (d, 6H, $^{3}J_{HH}$ = 7.8 Hz, H_e), 6.62 (d, 6H, $^{3}J_{HH}$ = 7.8 Hz, H_d), 1.29 (s, 27H, $CH_{3(tBu3)}$), 1.23 (s, 27H, $CH_{3(tBu3)}$). **^{13}C{^{1}H} NMR** (100 MHz, CD_2Cl_2, 25 ºC) δ: 151.2 (s, C_5), 150.5 (d, $^{3}J_{CP}$ = 7 Hz, C_2), 145.2 (d, $^{2}J_{CP}$ = 17 Hz, C_3), 138.0 (d, $^{3}J_{CP}$ = 7 Hz, C_4), 133.4 (d, $^{2}J_{CP}$ = 5 Hz, CH_a), 132.5 (d, $^{2}J_{CP}$ = 9 Hz, CH_c), 130.8 (d, $^{1}J_{CP}$ = 56 Hz, C_1), 129.4 (s, CH_d), 127.9 (s, CH_b), 125.9 (s, CH_e), 35.2 (s, $C_{(tBu3)}$), 34.9 (s, $C_{(tBu3)}$), 31.8 (s, $CH_{3(tBu3)}$), 31.5 (s, $CH_{3(tBu3)}$). **^{31}P{^{1}H} NMR** (162 MHz, CD_2Cl_2, 25 ºC) δ: 18.6.

Compound 3

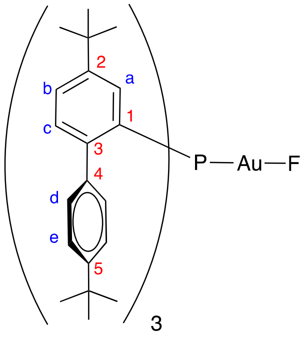

In an J. Young NMR tube a suspension of compound **2** (45 mg, 0.04 mmol) and AgF (15 mg, 0.12 mmol) in CH_2Cl_2 (1 mL) was subjected to sonication at rt 1 h. Then, the reaction mixture was filtered through a short pad of Celite to remove the silver salts and the solvent was removed under vacuum, thus yielding complex **3** as a colorless (30 mg, 75%). Crystals suitable for X-ray diffraction were grown by slow diffusion of toluene into a concentrated dichloromethane solution of complex **3**. **Anal. Calcd.** for $C_{60}H_{75}AuFP$: C, 69.08; H, 7.25. **Found:** C, 69.13; H, 7.22.**^{1}H NMR** (500 MHz, CD_2Cl_2, 25 ºC) δ: 7.56 (d, 3H, $^3J_{HH}$ = 8.1 Hz, H_b), 7.28–7.19 (m, 6H, H_a + H_c), 7.12 (d, 6H, $^3J_{HH}$ = 7.9 Hz, H_e), 6.79 (d, 6H, $^3J_{HH}$ = 7.9 Hz, H_d), 1.28 (s, 27H, $CH_{3(tBu3)}$), 1.21 (s, 27H, $CH_{3(tBu3)}$). **^{13}C{^{1}H} NMR** (125 MHz, CD_2Cl_2, 25 ºC) δ: 151.2 (s, C_5), 150.3 (d, $^3J_{CP}$ = 8 Hz, C_2), 145.3 (d, $^2J_{CP}$ = 15 Hz, C_3), 138.0 (d, $^3J_{CP}$ = 7 Hz, C_4), 132.5 (d, $^2J_{CP}$ = 6 Hz, CH_a), 132.1 (d, $^2J_{CP}$ = 9 Hz, CH_c), 129.8 (d, $^1J_{CP}$ = 62 Hz, C_1), 129.6 (s, CH_d), 127.9 (d, $^4J_{CP}$ = 3 Hz, CH_b), 125.2 (s, CH_e), 35.1 (s, $C_{(tBu3)}$), 34.9 (s, $C_{(tBu3)}$), 31.6 (s, $CH_{3(tBu3)}$), 31.4 (s, $CH_{3(tBu3)}$). **^{19}F{^{1}H} NMR** (470 MHz, CD_2Cl_2, 25 ºC) δ: –222.5 (d, $^2J_{PF}$ = 147 Hz). **^{31}P{^{1}H} NMR** (202 MHz, CD_2Cl_2, 25 ºC) δ: 1.7 (d, $^2J_{PF}$ = 147 Hz).

Compound 4

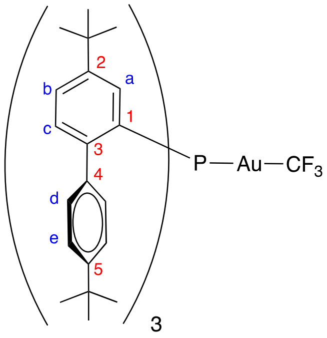

In an ampoule complex **1** (105 mg, 0.10 mmol) was dissolved in CH_2Cl_2 (5 mL) in the presence of $TMSCF_3$ (44 μL, 0.30 mmol) and AgF (126 mg, 1.00 mmol) and stirred in the dark at rt for 4 days. After this time, the reaction mixture was filtered through a short pad of Celite to remove the silver salts, and the solvent was removed under vacuum. The crude solid was prufied through column chromatography (SiO_2; Pentane/CH_2Cl_2 4:1), yielding complex **4** (70 mg, 70%) as a colorless solid. Crystals suitable for X-ray diffraction were grown by slow evaporation of a toluene solution of complex **4**. **Anal. Calcd.** for $C_{61}H_{75}AuF_3P$: C, 67.02, H, 6.92. **Found:** C, 67.29; H, 6.83. **^{1}H NMR** (500 MHz, $CDCl_3$, 25 ºC) δ: 7.54 (dt, 3H, $^3J_{HH}$ = 8.1 Hz, $^4J_{HH}$ = 1.7 Hz, H_b), 7.33–7.22 (m, 6H, H_a + H_c), 7.10 (d, 6H, $^3J_{HH}$ = 8.0 Hz, H_e), 6.61 (broad d, 6H, $^3J_{HH}$ = 8.0 Hz, H_d), 1.25 (s, 27H, $CH_{3(tBu3)}$), 1.21 (s, 27H, $CH_{3(tBu3)}$). **^{13}C{^{1}H} NMR** (125 MHz, $CDCl_3$, 25 ºC) δ: 161.2 (qd, $^1J_{CF}$ = 356 Hz, $^2J_{CP}$ = 180 Hz, CF_3), 150.6 (s, C_5), 149.8 (d, $^3J_{CP}$ = 7 Hz, C_2), 145.1 (d, $^2J_{CP}$ = 17 Hz, C_3), 137.4 (d, $^3J_{CP}$ = 6 Hz, C_4), 132.9 (d, $^2J_{CP}$ = 4 Hz, CH_a), 131.9 (d, $^2J_{CP}$ = 9 Hz, CH_c), 130.5 (d, $^1J_{CP}$ = 52 Hz, C_1), 129.1 (s, CH_d), 127.2 (d, $^4J_{CP}$ = 2 Hz, CH_b), 125.1 (s, CH_e), 34.8 (s, $C_{(tBu3)}$),

34.6 (s, $C_{(tBu3)}$), 31.3 (s, $CH_{3(tBu3)}$). **^{19}F{^{1}H} NMR** (470 MHz, $CDCl_3$, 25 ºC) δ: –27.2 (d, $^{3}J_{PF}$ = 41 Hz). **^{31}P{^{1}H} NMR** (202 MHz, $CDCl_3$, 25 ºC) δ: 22.2 (q, $^{3}J_{PF}$ = 41 Hz).

Compound 5

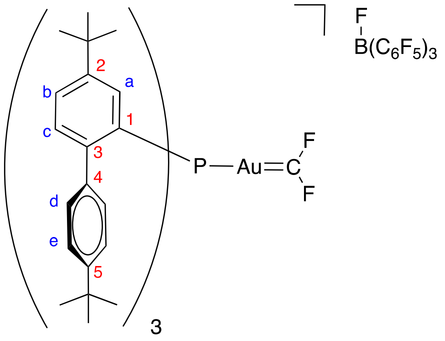

In an screw-cap NMR tube, complex **4** (30 mg, 0.023 mmol) was dissolved in CD_2Cl_2 (0.3 mL) and cooled down to –78 ºC. In parallel $B(C_6F_5)_3$ (14 mg, 0.027 mmol) was dissolved in CD_2Cl_2 (0.3 mL) in an ampoule and cooled down to –78 ºC. The borane solution was cannulated into the solution of complex **4** with an immediaty colour change from clear to bright yellow/orange, thus forming complex **5**, which was characterized in situ at –80 ºC.

^{1}H NMR (500 MHz, CD_2Cl_2, –80 ºC) δ: 7.71 (broad s, 3H, H_b), 7.47 (broad d, 3H, J_{HP} = 10 Hz, H_a), 7.39 (d, 3H, J_{HH} = 5 Hz, H_c), 7.27 (broad s, 3H, H_d), 7.14 (broad s, 3H, H_e), 7.08 (broad s, 3H, H_d), 6.05 (broad s, 3H, H_d), 6.05 (broad s, 3H, H_d), 1.20 (s, 27H, $CH_{3(tBu3)}$), 1.12 (s, 27H, $CH_{3(tBu3)}$). **^{11}B{^{1}H} NMR** (125 MHz, CD_2Cl_2, –80 ºC) δ: 1.6. **^{13}C{^{1}H} NMR** (125 MHz, CD_2Cl_2, –80 ºC) δ: 247.1 (td, $^{1}J_{CF}$ = 524 Hz, $^{2}J_{CP}$ = 134 Hz, CF_2), 151.3 (s, C_5), 151.3 (d, $^{3}J_{CP}$ = 7 Hz, C_2), 148.8 (br s, *o*-C_6F_5), 146.4 (br s, *p*-C_6F_5), 142.7 (d, $^{2}J_{CP}$ = 16 Hz, C_3), 137.7 (d, $^{3}J_{CP}$ = 8 Hz, C_4), 137.7 (br s *m*-C_6F_5), 135.5 (br s, *ipso*-C_6F_5), 132.4 (d, $^{2}J_{CP}$ = 4 Hz, CH_a), 132.3 (d, $^{2}J_{CP}$ = 9 Hz, CH_c), 129.6 (s, CH_b), 129.6 (s, CH_d), 129.2 (s, CH_d), 125.8 (d, $^{1}J_{CP}$ = 64 Hz, C_1), 125.7 (s, CH_e), 125.4 (s, CH_e). 34.8 (s, $C_{(tBu3)}$), 34.4 (s, $C_{(tBu3)}$), 30.7 (s, $CH_{3(tBu3)}$), 30.7 (s, $CH_{3(tBu3)}$). **^{19}F{^{1}H} NMR** (470 MHz, CD_2Cl_2, –80 ºC) δ: 165.2 (d, $^{3}J_{PF}$ = 28 Hz), –136.6 (br s, *o*-C_6F_5), –161.1 (br s, *p*-C_6F_5), –164.2 (br, *m*-C_6F_5), –191.7 (br s, BF). **^{31}P{^{1}H} NMR** (202 MHz, CD_2Cl_2, –80 ºC) δ: 9.6 (t, $^{3}J_{PF}$ = 28 Hz).

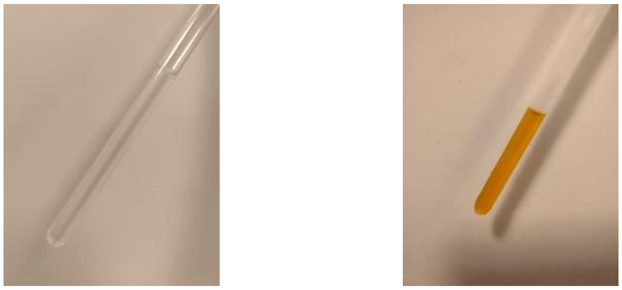

Figure S1. Clear solution of compound **4** in CD_2Cl_2 (left); bright yellow/orange solution after addition of $B(C_6F_5)_3$ into a solution of compound **4** in CD_2Cl_2 generating the gold(I) difluorcarbene complex **5** (right).

Compound 5'

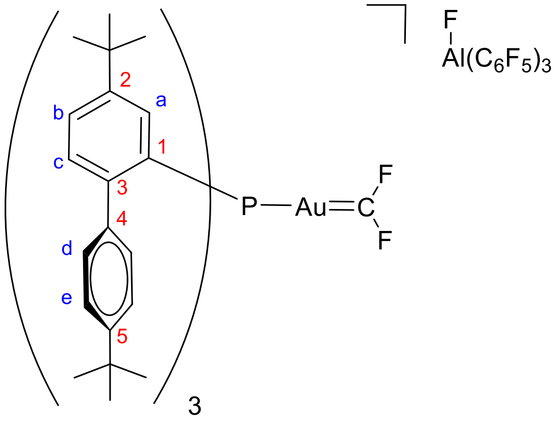

Compound **5'** was prepared following the same synthetic procedure employed for compound **5** but using $Al(C_6F_5)_3$ (14 mg, 0.027 mmol) instead of the analogous borane. Compound **5'** was characterized in situ at 25 ºC.

^{1}H NMR (400 MHz, CD_2Cl_2, 25 ºC) δ: 7.81 (d, 3H, $^3J_{HP}$ = 8 Hz, H_b), 7.56 (d, 3H, $^3J_{HP}$ = 12 Hz, H_a), 7.48 (t, 3H, $^3J_{HH}$ = 6 Hz, H_c), 7.26 (d, $^3J_{HH}$ = 8 Hz, 6H, H_e), 6.72 (broad s, 6H, H_d), 1.31 (s, 27H, $CH_{3(tBu3)}$), 1.25 (s, 27H, $CH_{3(tBu3)}$). **^{19}F{^{1}H} NMR** (376 MHz, CD_2Cl_2, –80 ºC) δ:–122.8 (br s, *o*-C_6F_5), –154.9 (br s, *p*-C_6F_5), 162.1 (br s, AlF), –163.6 (br, *m*-C_6F_5). **^{31}P{^{1}H} NMR** (162 MHz, CD_2Cl_2, 25 ºC) δ: 9.8 (t, $^3J_{PF}$ = 31 Hz).

Experimental procedure of gold-mediated =CF_2 transfer to *E*- and *Z*-stilbene

In parallel three different solutions were preapared into the respective J-Young NMR tubes and cooled down to –78 ºC:

Solution A: complex **4** (10 mg, 0.010 mmol, 1.00 equiv.) in 0.2 mL of CD_2Cl_2.

Solution B: $B(C_6F_5)_3$ (6 mg, 0.012 mmol, 1.20 equiv.) in 0.2 mL of CD_2Cl_2.

Solution C: *E*-stilbene or *Z*-stilbene (4 μL, 0.020 mmol, 2.00 equiv.) in 0.2 mL of CD_2Cl_2.

Firstly, solution B containing the $B(C_6F_5)_3$ was transferred onto solution A containing complex **4**. An immediate colour change from clear to bright yellow/orange was observed (Figure S1). Then, solution C with *E*- or *Z*-stilbene was transfered via canula to the reaction mixture and the reaction was slowly warmed up to rt and left it overnight (18 h). The reaction mixture was analized by ^{19}F{1H} NMR using 4-(trifluoromethyl)anisole (3 μL, 0.020 mmol, 2.00 equiv.) as internal standard.

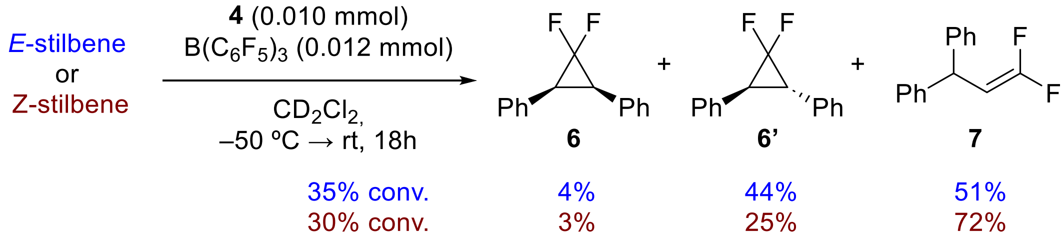

2. NMR spectroscopic experiments

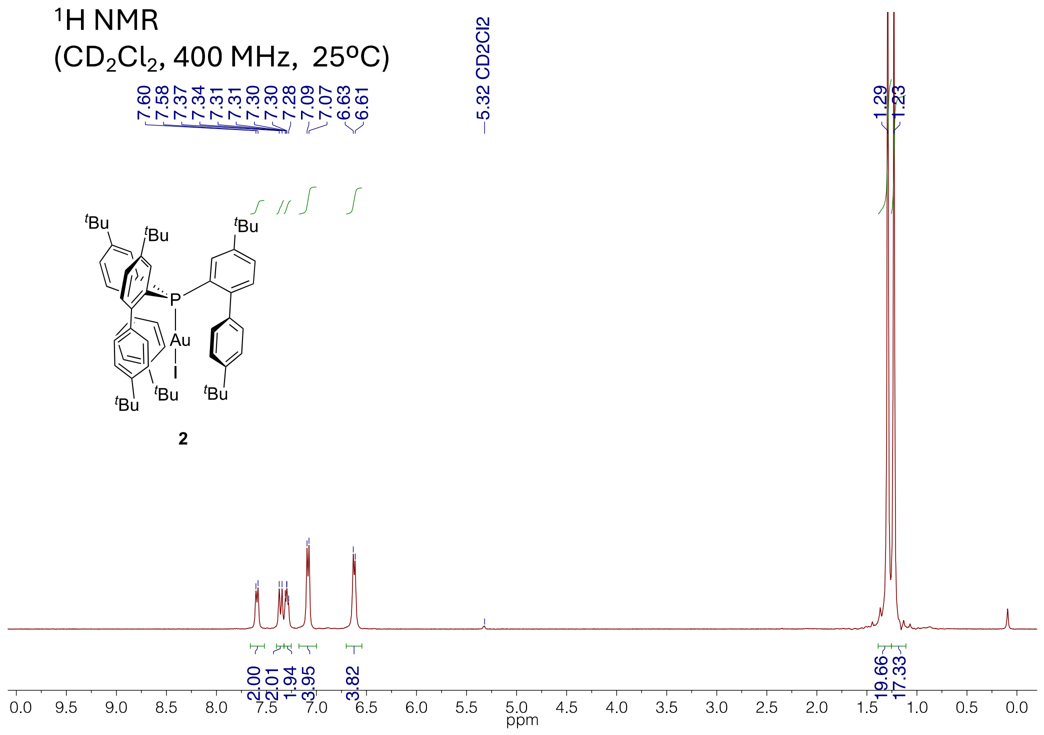

Figure S2. ^{1}H NMR of complex **2** in CD_2Cl_2 at 298K.

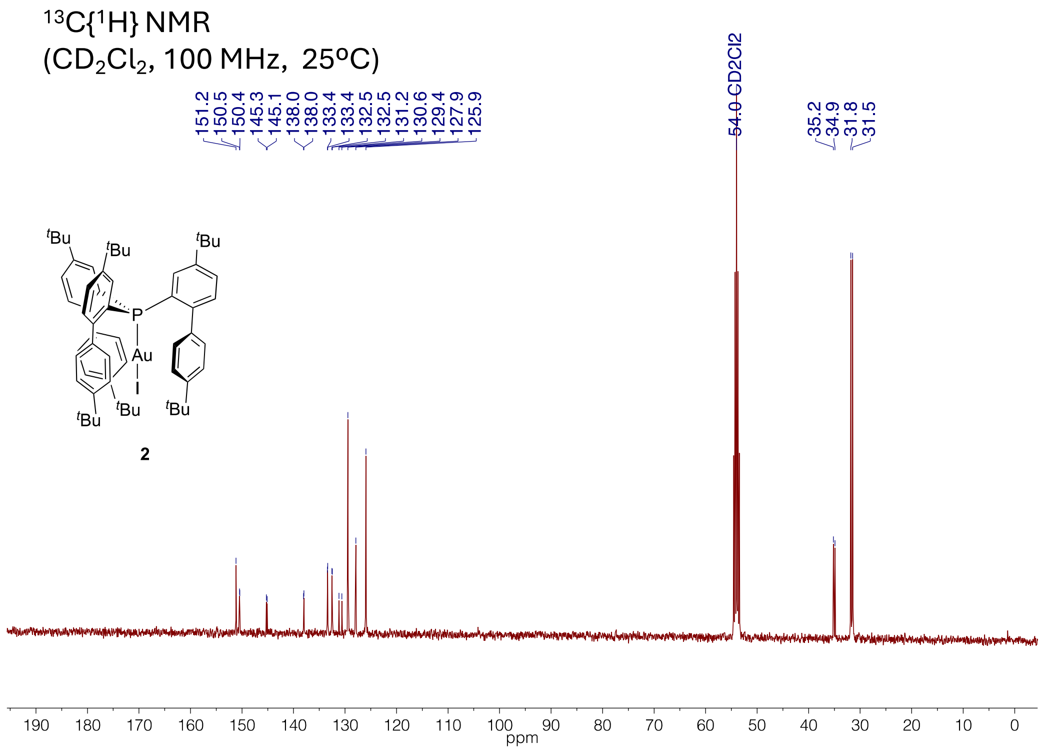

Figure S3. $^{13}C\{^{1}H\}$ NMR of complex **2** in CD_2Cl_2 at 298K.

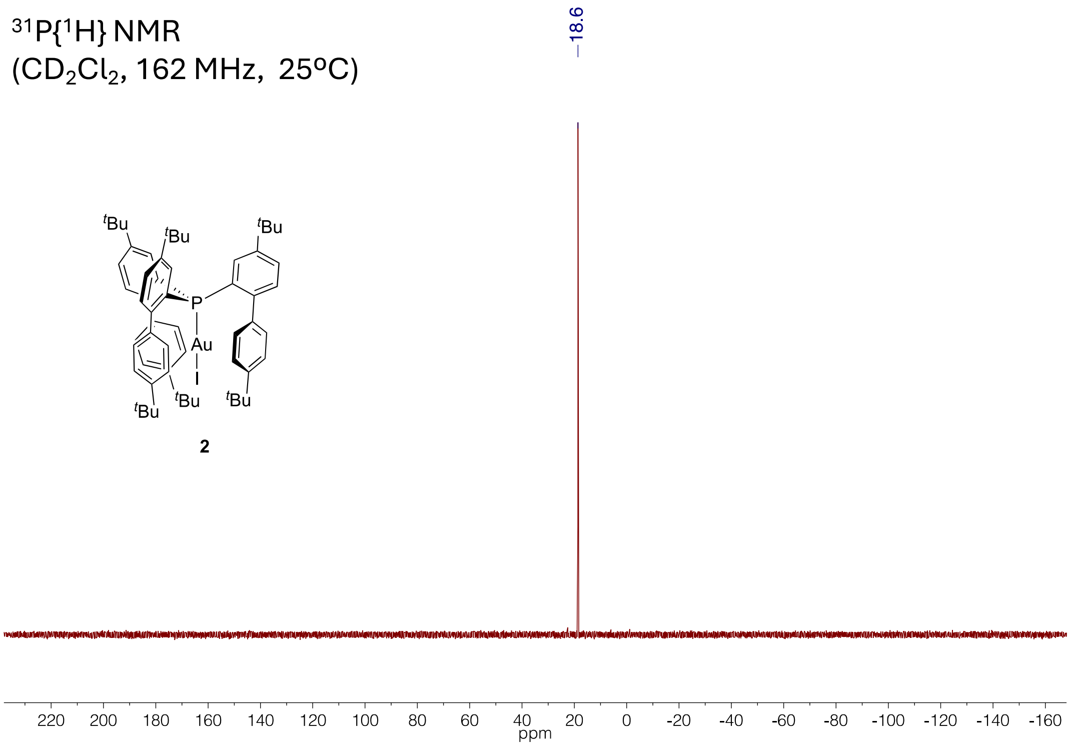

Figure S4. $^{31}P\{^1H\}$ NMR of complex **2** in CD_2Cl_2 at 298K.

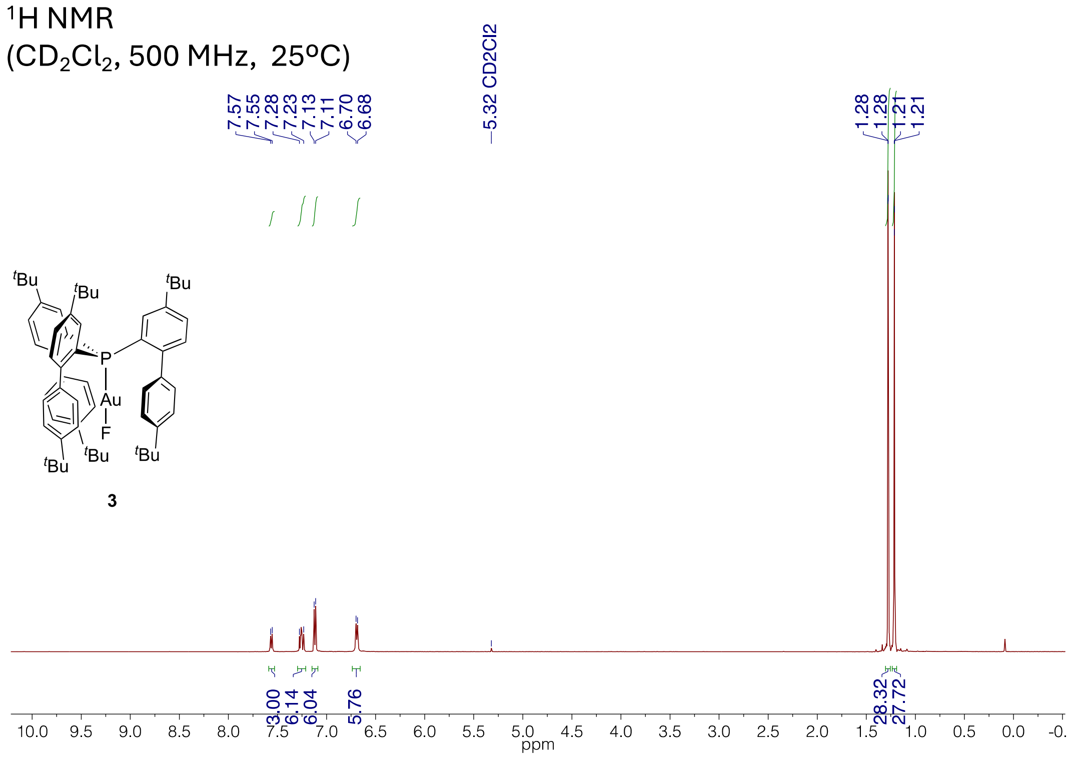

Figure S5. ^1H NMR of complex **3** in CD_2Cl_2 at 298K.

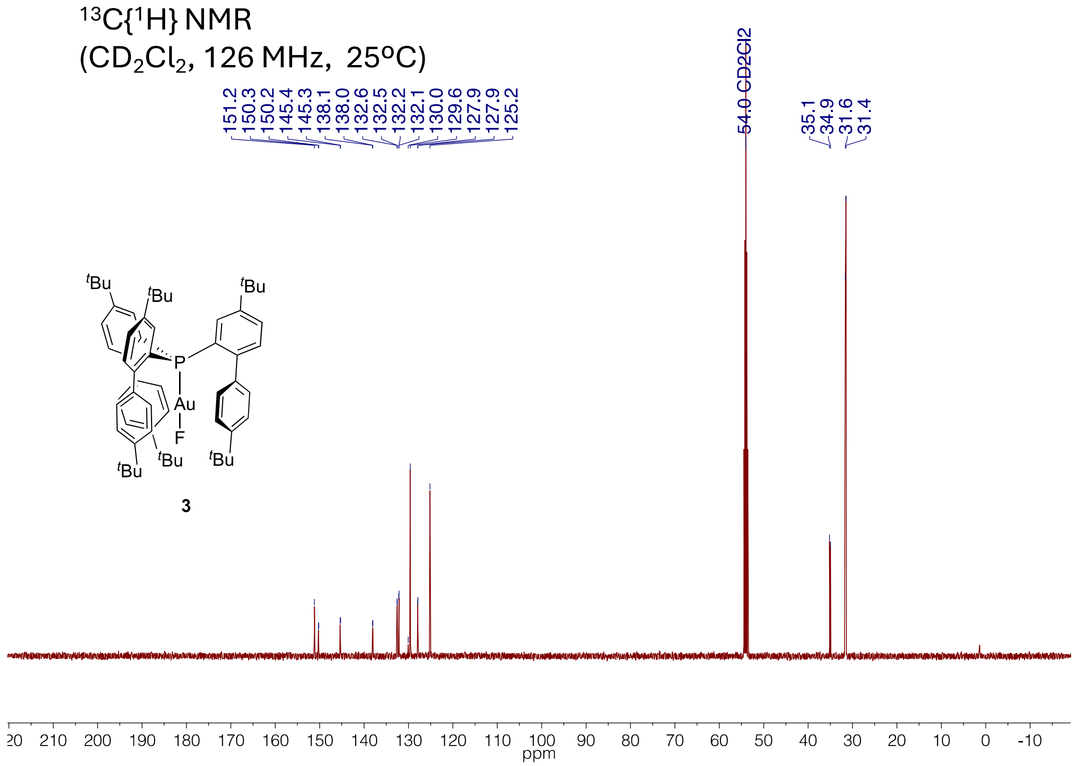

Figure S6. $^{13}C\{^1H\}$ NMR of complex **3** in CD_2Cl_2 at 298K.

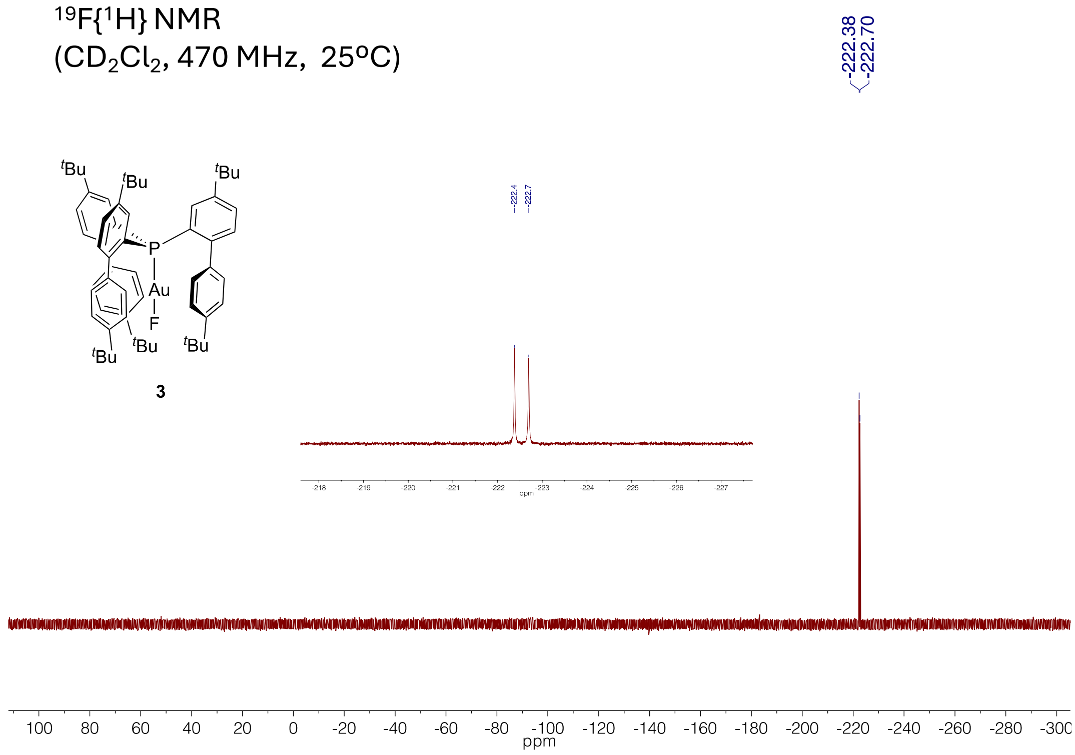

Figure S7. $^{19}F\{^1H\}$ NMR of complex **3** in CD_2Cl_2 at 298K.

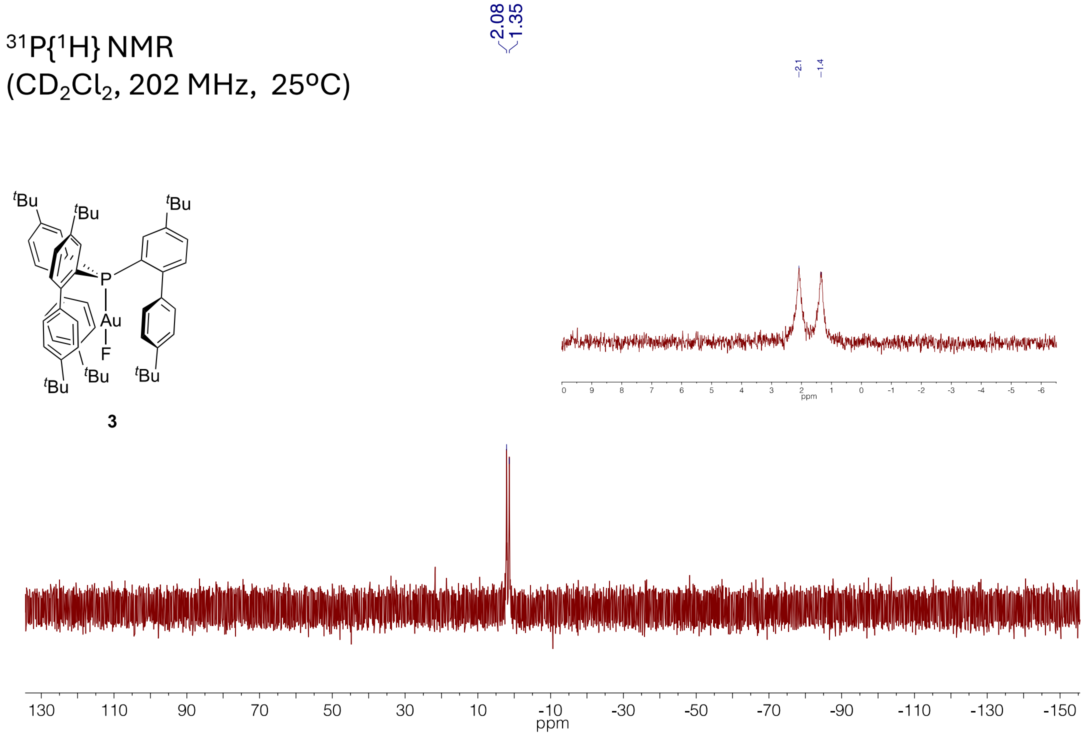

Figure S8. $^{31}P\{^1H\}$ NMR of complex **3** in CD_2Cl_2 at 298K.

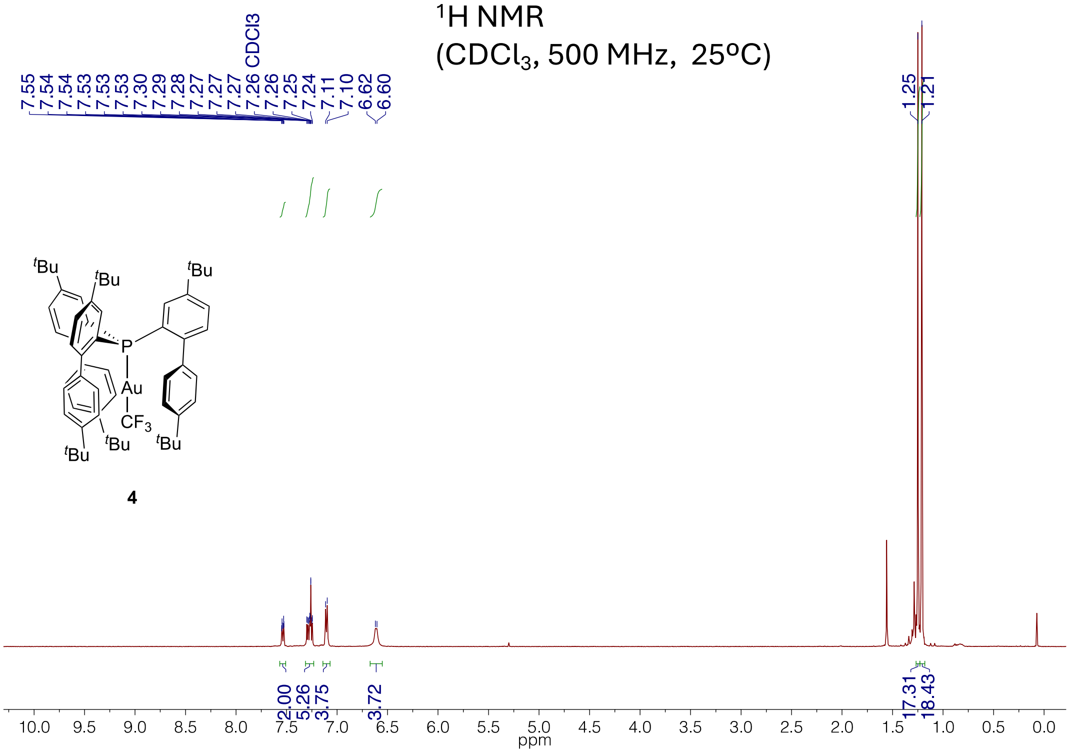

Figure S9. ^1H NMR of complex **4** in $CDCl_3$ at 298K.

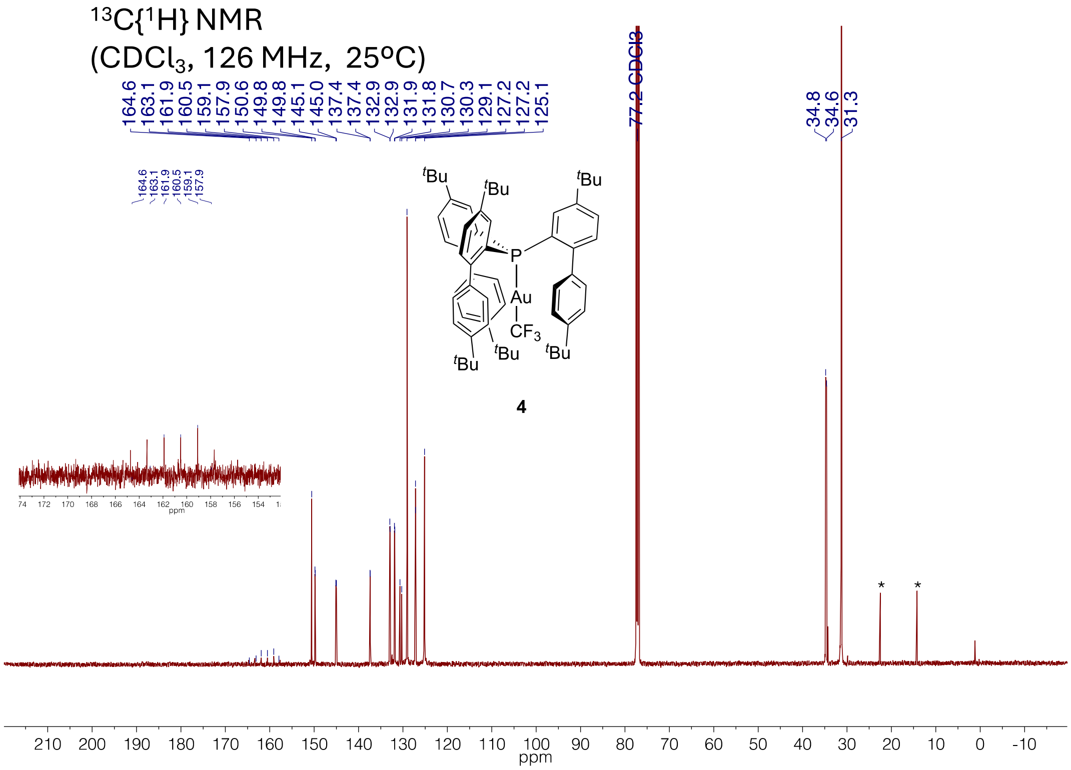

Figure S10. $^{13}C\{^{1}H\}$ NMR of complex **4** in $CDCl_3$ at 298K (* denotes n-pentane).

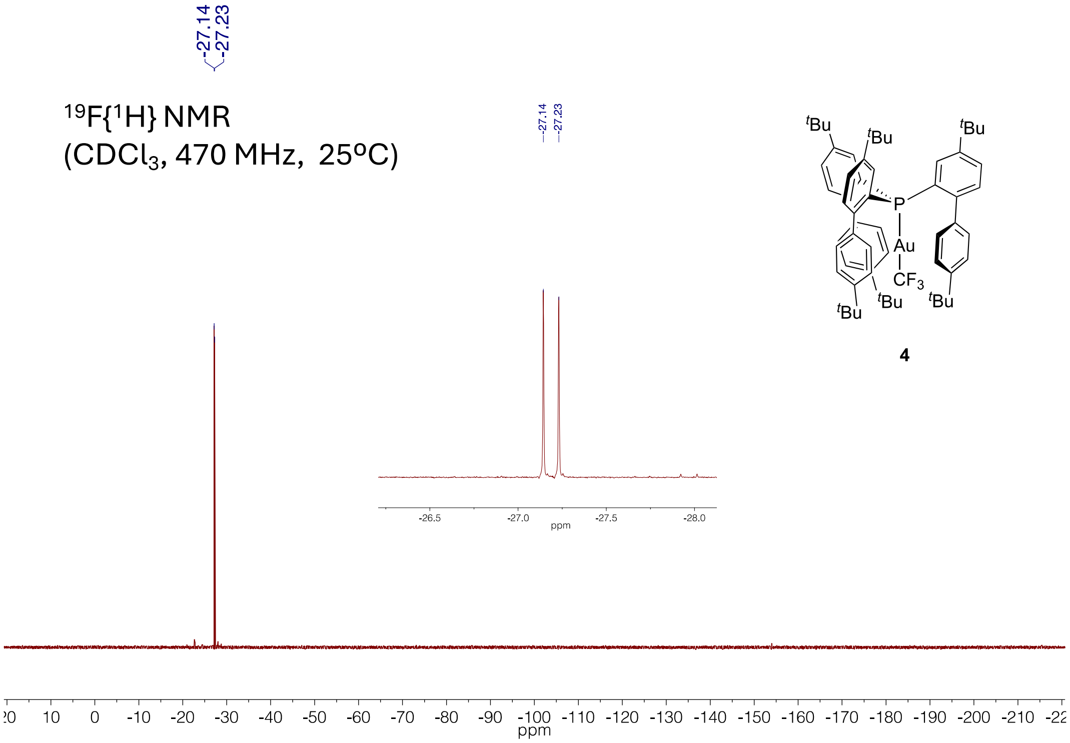

Figure S11. $^{19}F\{^{1}H\}$ NMR of complex **4** in $CDCl_3$ at 298K.

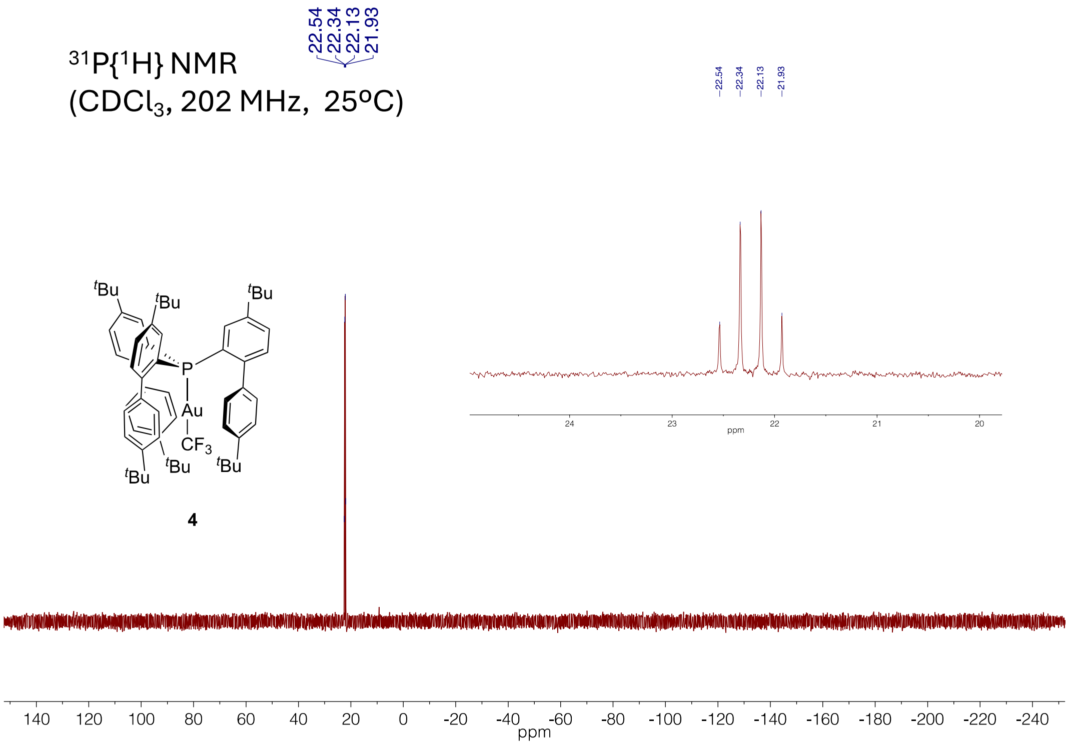

Figure S12. ^{31}P{^{1}H} NMR of complex **4** in CDCl$_3$ at 298K.

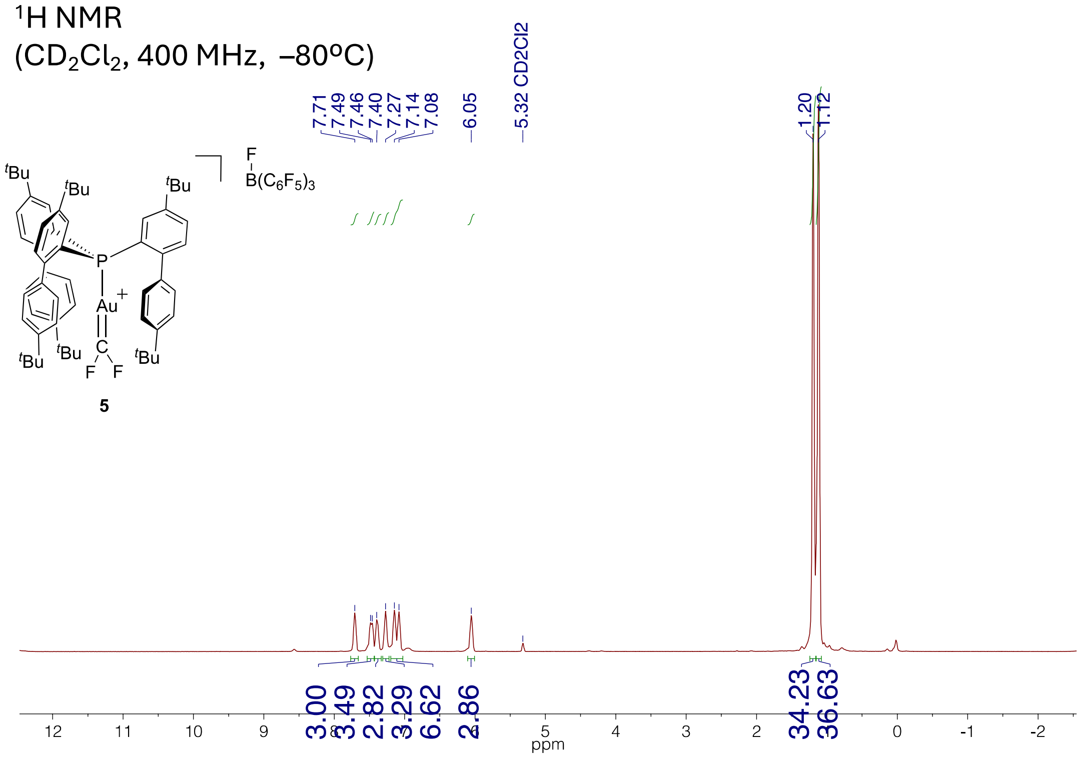

Figure S13. ^{1}H NMR of complex **5** in CD2Cl$_2$ at 193K.

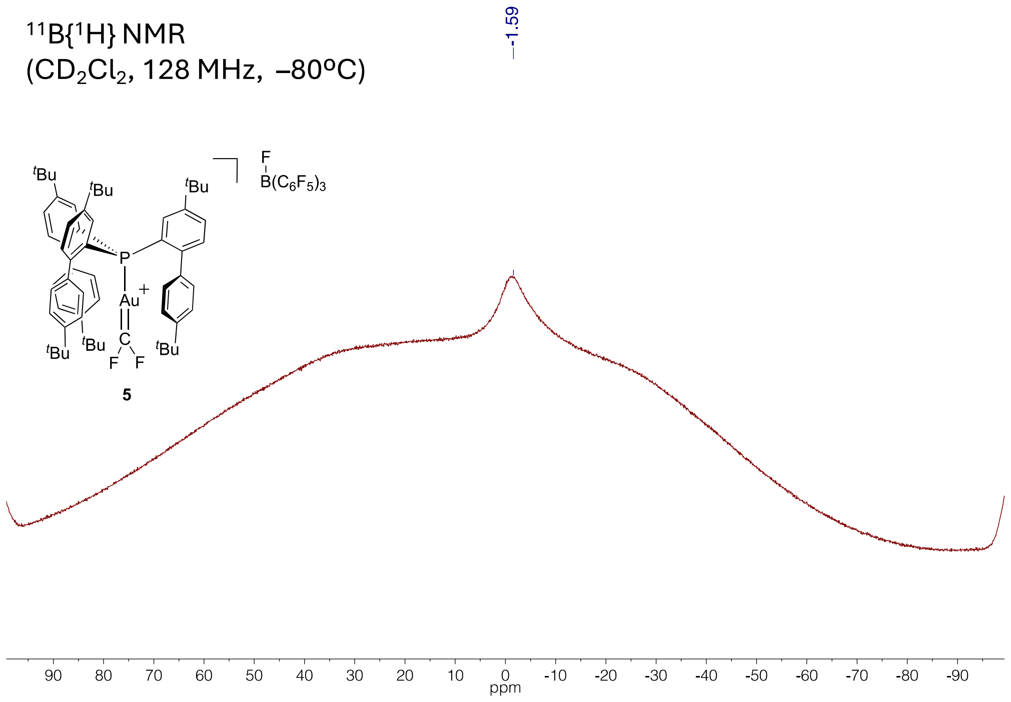

Figure S14. $^{11}B\{^{1}H\}$ NMR of complex **5** in CD_2Cl_2 at 193K.

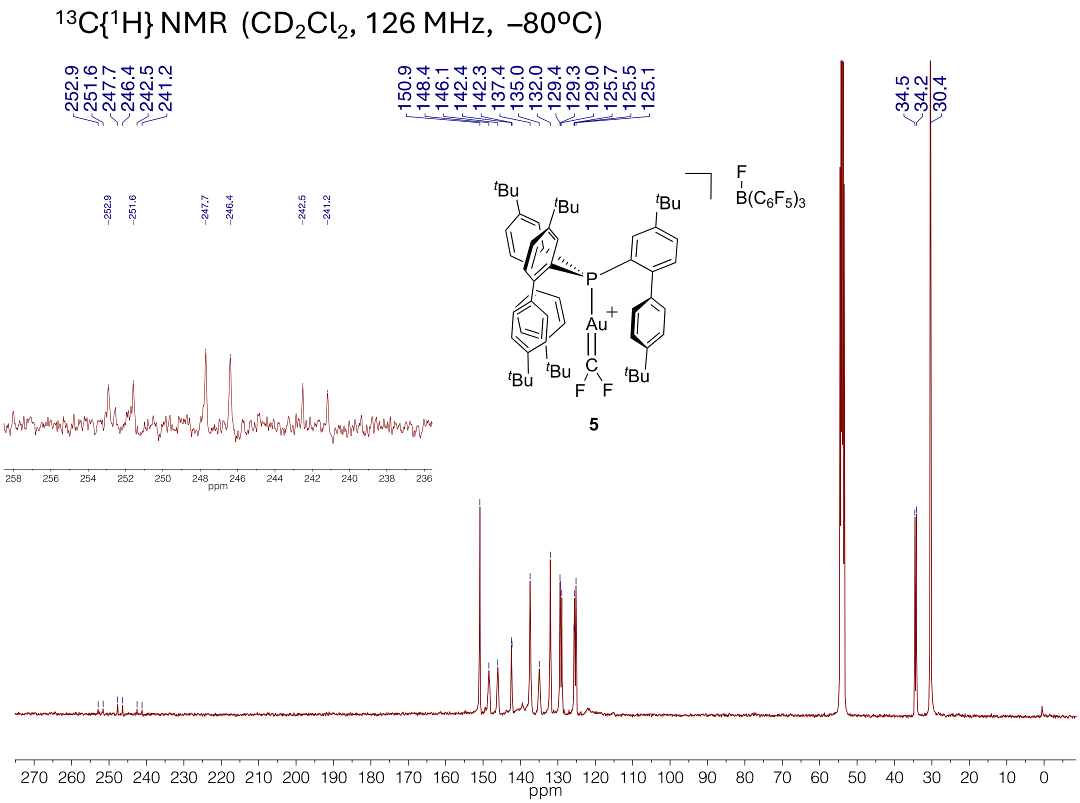

Figure S15. $^{13}C\{^{1}H\}$ NMR of complex **5** in CD_2Cl_2 at 193K.

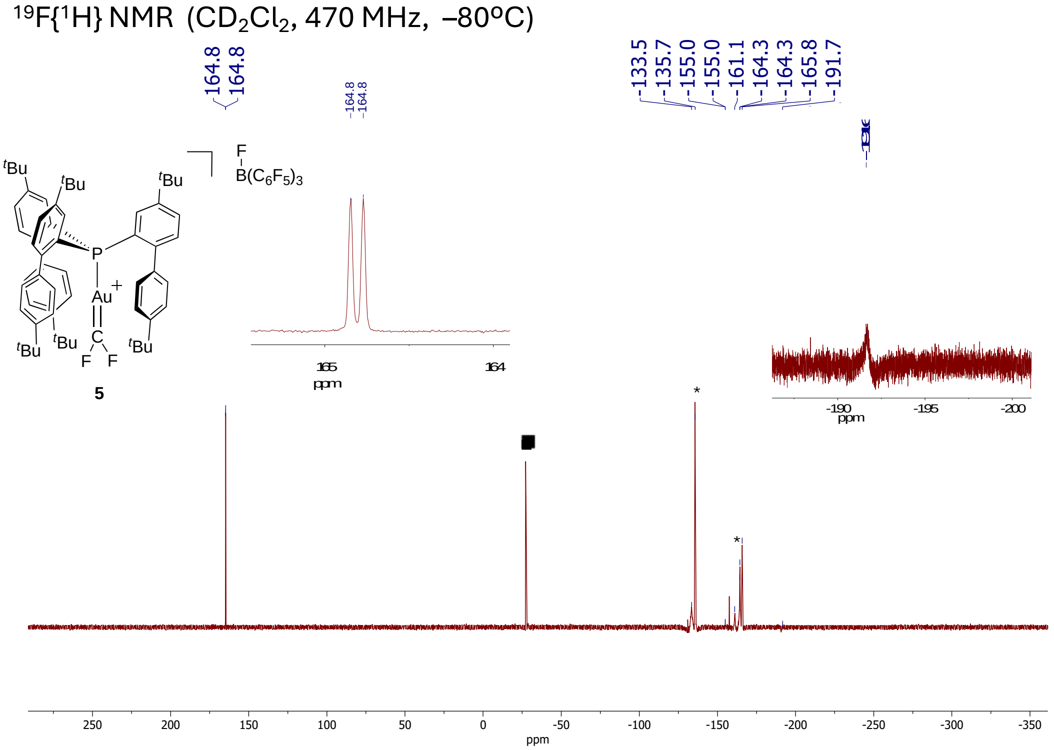

Figure S16. $^{19}F\{^{1}H\}$ NMR of complex **5** in CD_2Cl_2 at 193K (■ denotes unreacted compound **4** and * denotes excess of $B(C_6F_5)_3$).

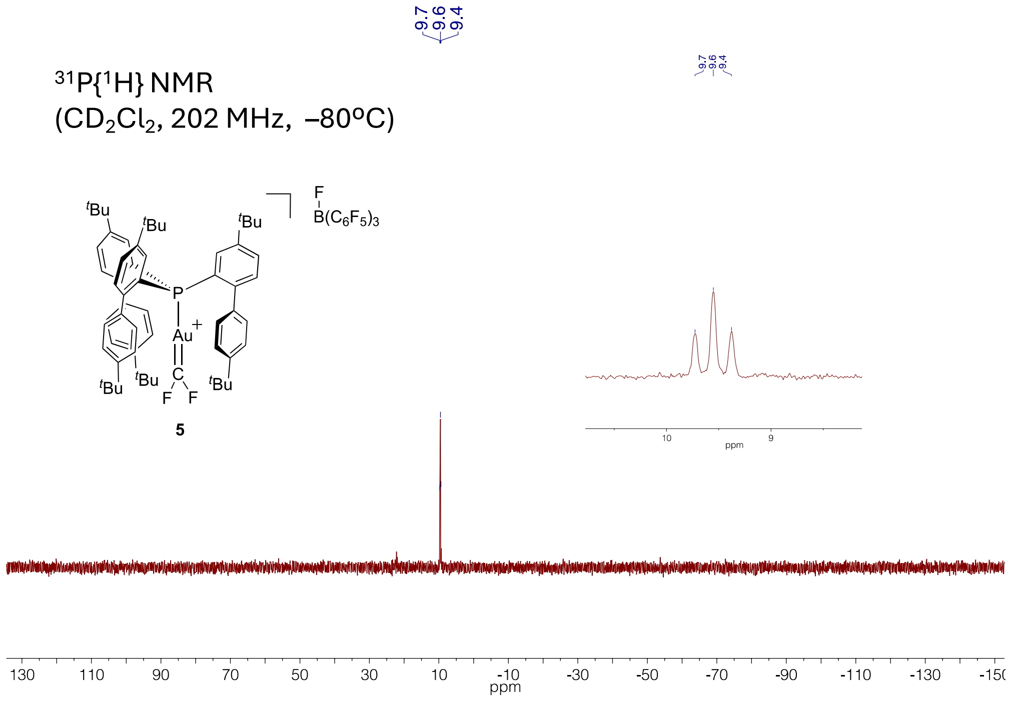

Figure S17. $^{31}P\{^{1}H\}$ NMR of complex **5** in CD_2Cl_2 at 193K.

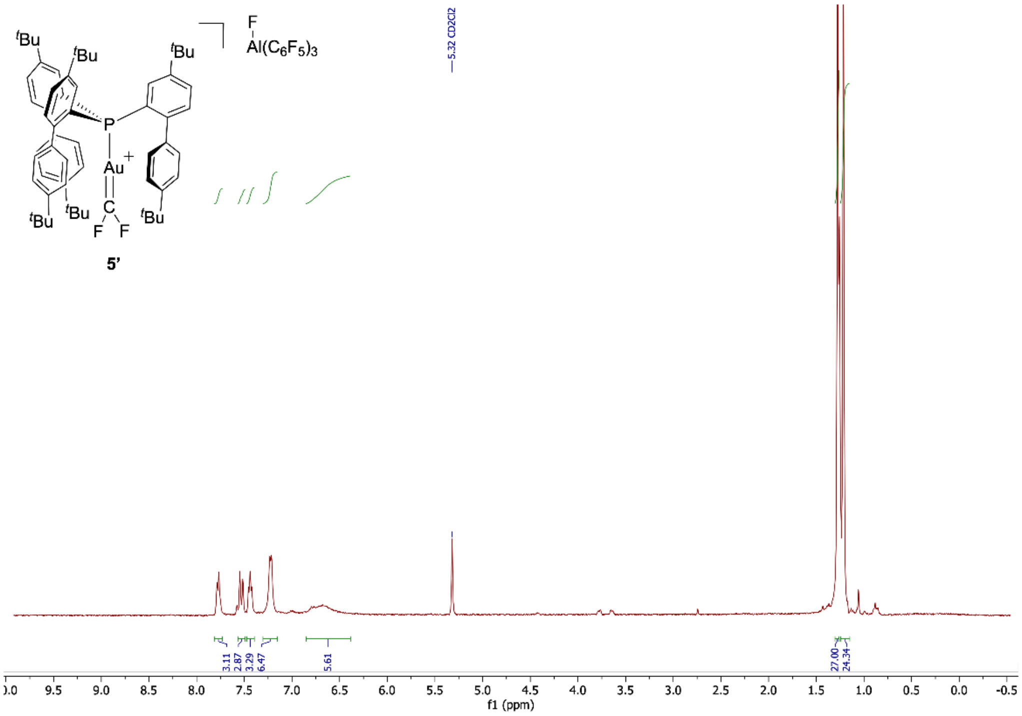

Figure S18. ^{1}H NMR of complex **5'** in CD_2Cl_2 at 298K.

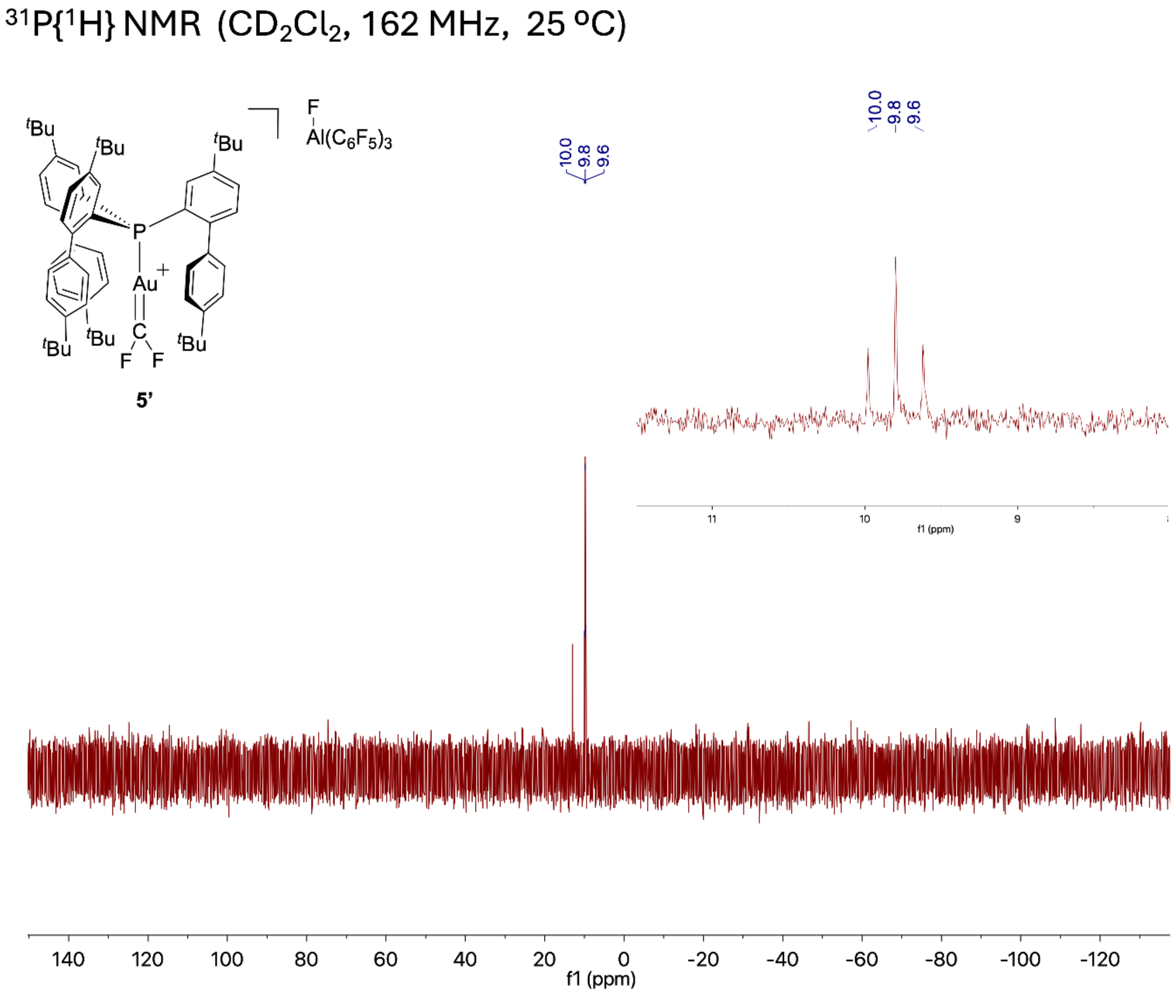

Figure S19. $^{31}P\{^1H\}$ NMR of complex **5’** in CD_2Cl_2 at 298K.

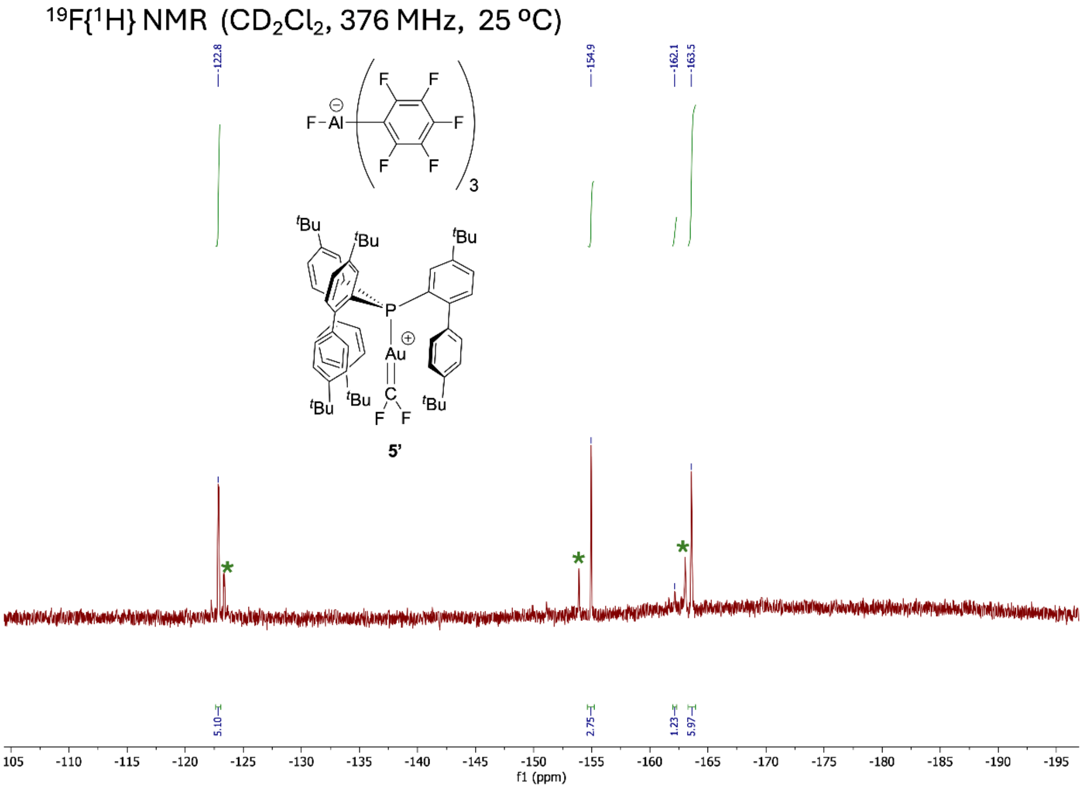

Figure S20. $^{19}F\{^{1}H\}$ NMR of the aluminate region of complex **5’** in CD_2Cl_2 at 298K. * Denotes excess of unreacted $Al(C_6F_5)_3$.

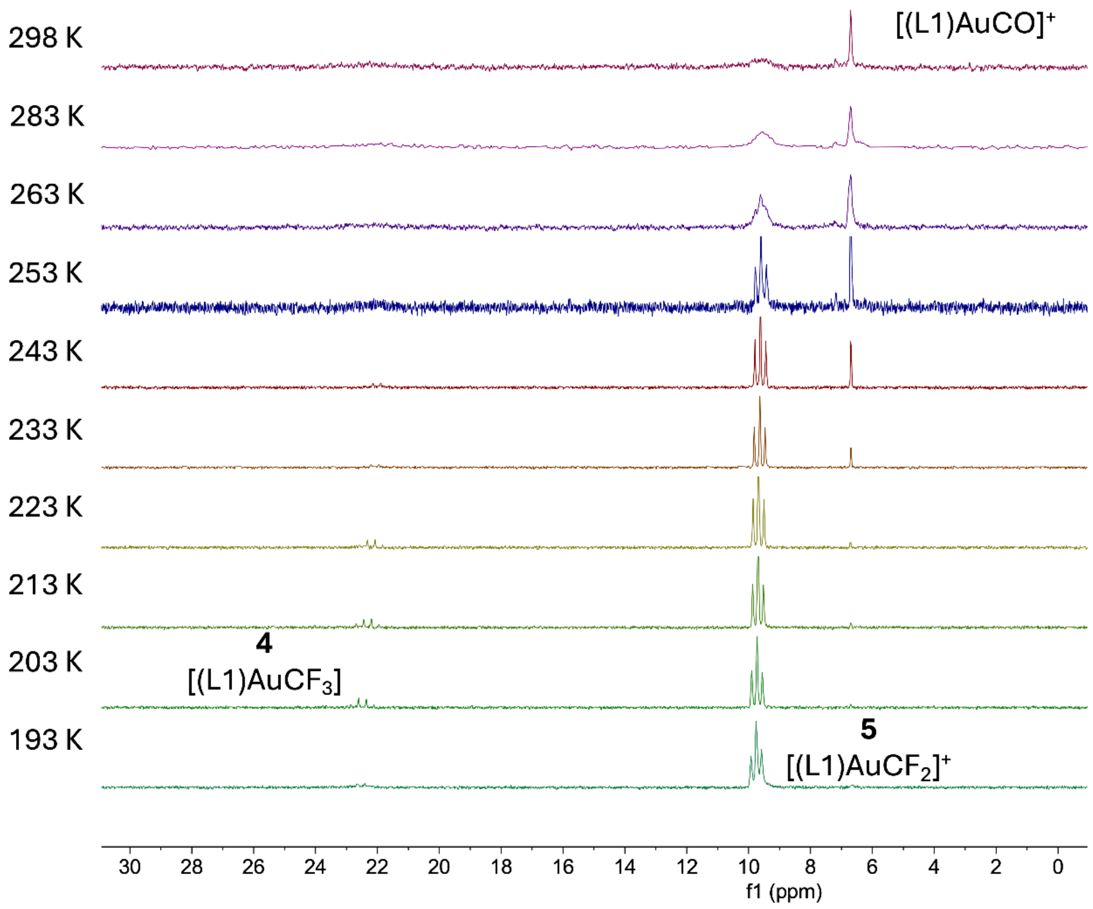

Figure S21. Variable temperature $^{31}P\{^{1}H\}$ NMR (CD_2Cl_2, 162 MHz) of complex **5**.

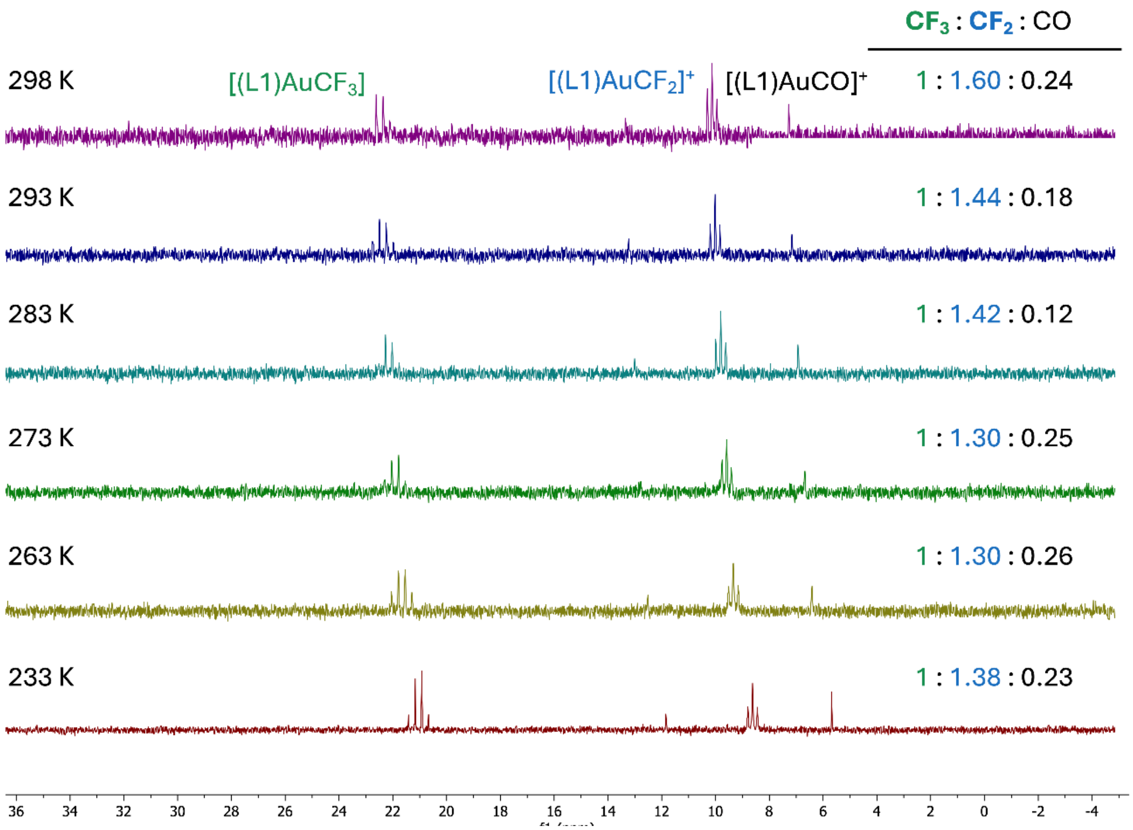

Figure S22. Variable temperature $^{31}P\{^{1}H\}$ NMR (CD_2Cl_2, 162 MHz) of complexes **5’** in a mixture containing compounds **4** and [**L1**−Au−CO]$^{+}$.

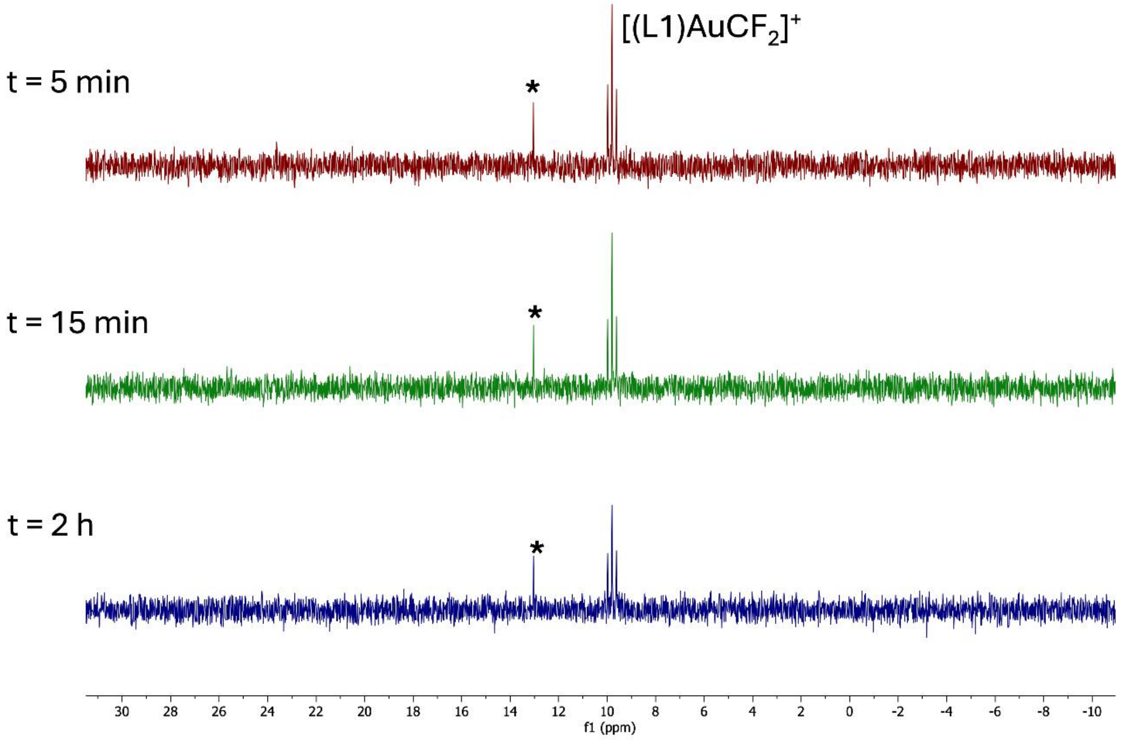

Figure S23. $^{31}P\{^{1}H\}$ NMR (CD_2Cl_2, 162 MHz) monitoring of complex **5’** at 25 ºC. * Denotes an impurity already accompanying precursor **4** which serves as a standard to evince the stability of **5’** at room temperature.

3. Crystal structure determinations

Crystallographic details. Single crystals of suitable size, coated with dry perfluoropolyether or FLOMBLIN oil were mounted on a glass fiber and fixed in a cold nitrogen stream [T = 193 K] to the goniometer head. Data collection was performed on a Bruker D8 Quest APEX-III CCD area detector PhotonIII using monochromatic radiation λ (Mo Kα1) = 0.71073 Å by a IμS 3.0 microfocus X-ray source at the Instituto de Investigaciones Químicas, Sevilla. Data collections were processed with APEX-W2D-NT (Bruker, 2004), cell refinement and data reduction with SAINT-Plus (Bruker, 2004) and the absorption was corrected by multiscan method applied by SADABS. The space-group assignment was based upon systematic absences, E statistics, and successful refinement of the structure. The structures were solved with SHELXT and was refined against F2 on all data by full-matrix least squares with SHELXL,[2] using Olex2[3] as graphical interface. All non-hydrogen atoms were refined anisotropically. Hydrogen atoms were included in the model at geometrically calculated positions and refined using a riding model, unless otherwise noted. The isotropic displacement parameters of all hydrogen atoms were fixed to 1.2 times the U value of the atoms to which they are linked (1.5 times for methyl groups). Atomic coordinates, anisotropic displacement parameters and bond lengths and angles can be found in the cif files which have been deposited in the Cambridge Crystallographic Data Centre with no. 2440124-2440126. These data can be obtained free of charge from The Cambridge Crystallographic Data Centre via www.ccdc.cam.ac.uk/data_request/cif. All structures contain solvent molecules within the unit cell which have been located in the Fourier electron density map (one toluene (**4**) and one dichloromethane (**3**) molecules). Compounds **2** and **4** also contained several toluene molecules per formula unit which have been treated as a diffuse contributions using a solvent mask for 552 and 105 electrons, respectively, that were found in volume of 3747 (3 voids) and 449 $Å^3$ (1 void) per unit cell. In compound **3** there is a weak hydrogen bond between the terminal Au−F and H62A from the solvent dichloromethane molecule defined by a F1⋯C62 distance of 3.116Å and a F1⋯H62A−C62 angle of 176.43º. Some of the tert-butyl groups were modelled over two positions and refined accordingly using the corresponding restraints. A summary of all crystallographic data and refinement parameters for each compound is provided in Table S1.

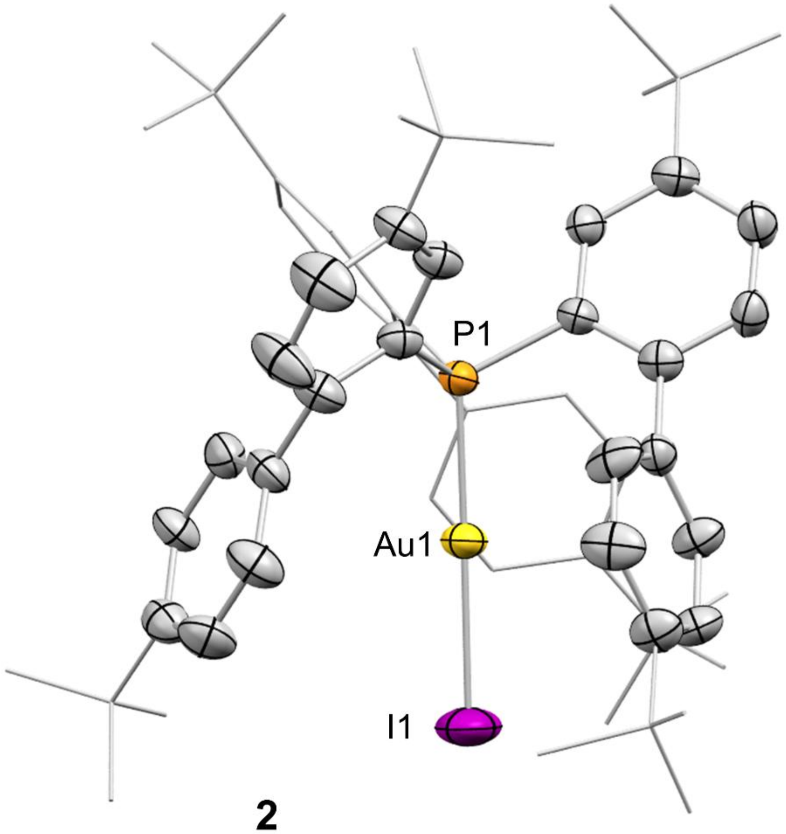

2

Figure S24. ORTEP diagram of compound **2**. Solvent molecules and hydrogen atoms are excluded for clarity, while *tert*-butyl groups and one biaryl fragment are represented in wireframe format. Thermal ellipsoids are set at 50% probability.

Table S1. Crystal data and structure refinement for compounds **2**, **3** and **4**.

	2	***3***	***4***
Formula	$C_{67}H_{83}AuPI$	$C_{67}H_{83}AuPI \cdot CH_2Cl_2$	$C_{68}H_{83}AuF_3P \cdot C_7H_8$
FW	1242.49	1220.27	1277.49
T/K	193.00	193.00	193.00
Crystal system	trigonal	monoclinic	triclinic
Space group	R-3	$P2_1/n$	P-1
a/Å	48.480(3)	17.0222(13)	12.7393(6)
b/Å	48.480(3)	18.2721(15)	14.2947(6)
c/Å	13.2437(9)	20.0522(14)	20.2696(10)
α/°	90	90	70.446(2)
β/°	90	112.733(3)	78.307(2)
γ/°	120	90	75.098(2)
Volume/Å^3	26956(3)	5752.4(8)	3333.7(3)
Z	24	6	2
ρ_{calc} g/cm^3	1.327	1.303	1.181
μ/mm^{-1}	3.033	2.717	2.274
F(000)	10925.0	2320.0	1224.0
Size/mm^3	0.1 × 0.1 × 0.08	0.14 × 0.12 × 0.1	0.2 × 0.2 × 0.1
Radiation	MoKα (λ = 0.71073)	MoKα (λ = 0.71073)	MoKα (λ = 0.71073)
2Θ range data collection/°	3.636 to 56.538	4 to 50.498	4.056 to 56.646
Index ranges	$-61 \leq h \leq 64$, $-64 \leq k \leq 59$, $-17 \leq l \leq 13$	$-20 \leq h \leq 20$, $-21 \leq k \leq 21$, $-24 \leq l \leq 23$	$-16 \leq h \leq 16$, $-19 \leq k \leq 19$, $-27 \leq l \leq 27$
Reflections collected	97617	73507	131993
Independent reflections	14844 [R_{int} = 0.1664, R_{sigma} = 0.1010]	10413 [R_{int} = 0.1321, R_{sigma} = 0.0720]	16565 [R_{int} = 0.0588, R_{sigma} = 0.0308]
Data/restraints/parameters	14844/0/586	10413/63/638	16565/78/715
Goodness-of-fit on F^2	0.845	1.062	1.048
Final R indexes [I>=2σ (I)]	R_1 = 0.0667, wR_2 = 0.1534	R_1 = 0.0477, wR_2 = 0.1113	R_1 = 0.0325, wR_2 = 0.0755
Final R indexes [all data]	R_1 = 0.1308, wR_2 = 0.1921	R_1 = 0.0927, wR_2 = 0.1417	R_1 = 0.0428, wR_2 = 0.0803
Largest diff. peak/hole eÅ^{-3}	1.07/-1.46	2.02/-1.40	0.67/-0.70
CCDC number	2440124	2440126	2440125

4. Computational details

Calculations were performed at the DFT level with the Gaussian 09 (Revision D.01) program.[4] The hybrid functional PBE0[5] was used throughout the computational study, and dispersion effects were accounted for by using Grimme's D3 parameter set with Becke–Johnson (BJ) damping at the optimization stage.[6] Geometry optimizations were carried out without geometry constraints, using the 6-31G(d,p)[7] basis set to represent the C, H, B, F and P atoms and the Stuttgart/Dresden Effective Core Potential and its associated basis set (SDD)[8] to describe the Au atoms. Bulk solvent effects (dichloromethane) were included at the optimization stage with the SMD continuum model[9]. The stationary points and their nature as minima or saddle points (TS) were characterized by vibrational analysis, which also produced enthalpy (H), entropy (S) and Gibbs energy (G) data at 298.15 K. The minima connected by a given transition state were determined by perturbing the transition states along the TS coordinate and optimizing to the nearest minimum.

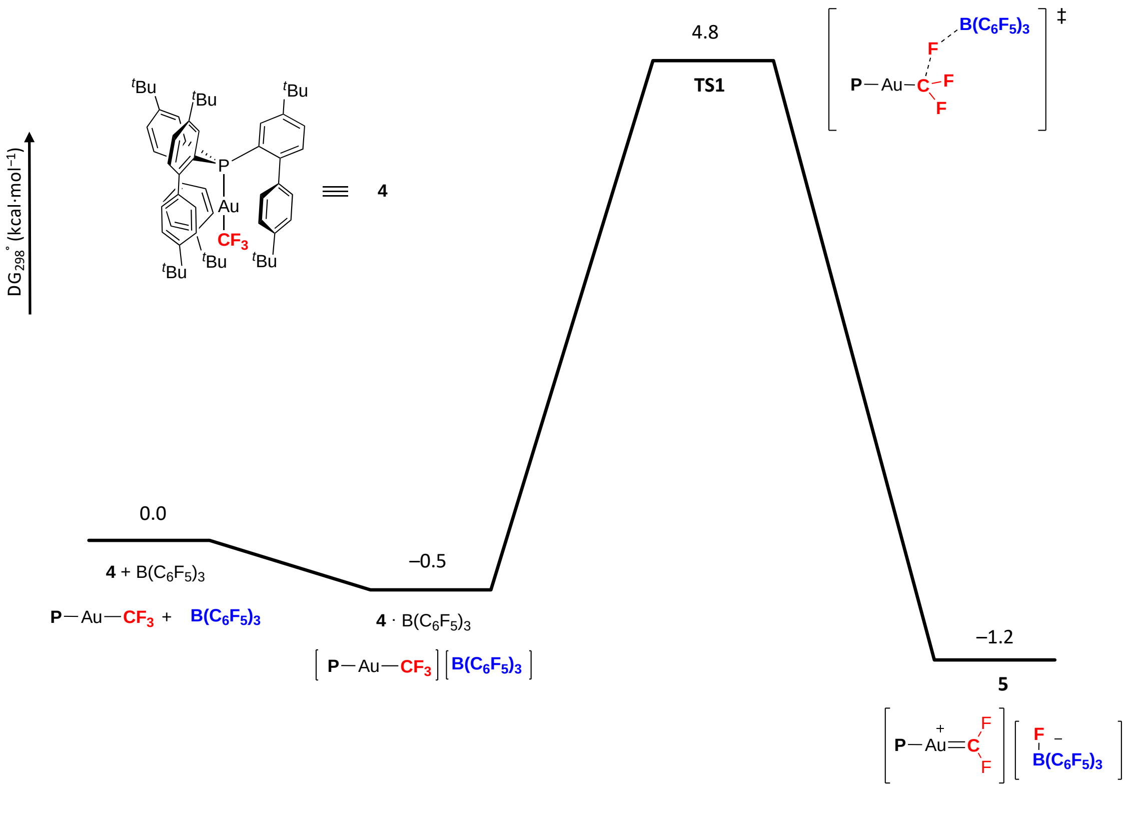

Figure S25. Free energy profile for the reaction of complex **4** with $B(C_6F_5)_3$ to generate complex **5**.

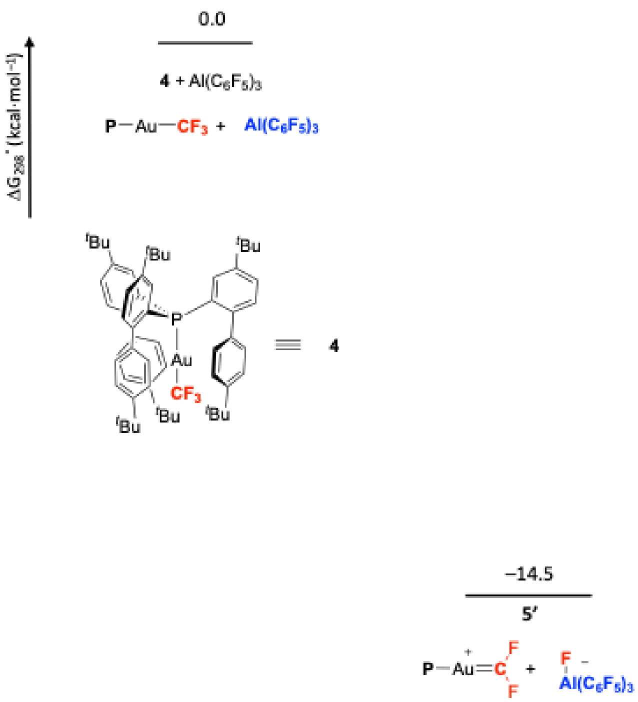

Figure S26. Calculated free energies for complex **4** with $B(C_6F_5)_3$ and complex **5'**.

4.1 Energy Decomposition Analysis Calculations

To enable a direct comparison with the available bonding analyses on related gold(I) complexes (see main text), the geometry of complex **4** (as well as complexes A and B) was re-optimized at the dispersion corrected BP86[10]-D3[11]/def2-SVP[12] level. The interaction between the transition metal fragment and the groups F, CF_3 and CF_2 has been investigated with the EDA-NOCV method,[13] which combines the energy decomposition analysis (EDA)[14] with the natural orbitals for chemical valence (NOCV)[15] methods. Within this approach, the interaction energy can be decomposed into the following physically meaningful terms:

$$\Delta E_{int} = \Delta E_{elstat} + \Delta E_{Pauli} + \Delta E_{orb} + \Delta E_{disp}$$

The term ΔE_{elstat} corresponds to the classical electrostatic interaction between the unperturbed charge distributions of the deformed reactants and is usually attractive. The Pauli repulsion ΔE_{Pauli} comprises the destabilizing interactions between occupied orbitals and is responsible for any steric repulsion. The orbital interaction ΔE_{orb} accounts for charge transfer (interaction between occupied orbitals on one moiety with

unoccupied orbitals on the other, including HOMO–LUMO interactions) and polarization (empty-occupied orbital mixing on one fragment due to the presence of another fragment). Finally, the ΔE_{disp} term takes into account the interactions which are due to dispersion forces.

The EDA-NOCV method makes it possible to further partition the total orbital interactions into pairwise contributions of the orbital interactions. Details of the method can be found in the literature.[16]

The EDA-NOCV calculations were carried out using the BP86-D3/def2-SVP optimized geometries with the program package ADF 2020[17] using the same functional (BP86-D3) in conjunction with a triple-ζ-quality basis set using uncontracted Slater-type orbitals (STOs) augmented by two sets of polarization function with a frozen-core approximation for the core electrons.[18] An auxiliary set of s, p, d, f, and g STOs were used to fit the molecular densities and to represent the Coulomb and exchange potentials accurately in each SCF cycle.[19] Scalar relativistic effects were incorporated by applying the zeroth-order regular approximation (ZORA).[20] This level of theory is denoted ZORA-BP86-D3/TZ2P//BP86-D3/def2-SVP.

Table S2. EDA-NOCV data (in kcal/mol) computed for complexes **3**, **4** and **5**.

	**3**	**4**	**5**
ΔEint	–250.7	–397.0	–254.5
ΔEPauli	96.5	238.5	199.3
ΔEelstat[a]	–162.6 (64.9%)	–293.6 (74.0%)	–167.1 (65.7%)
ΔEorb[a]	–85.1 (33.9%)	–92.6 (23.3%)	–76.8 (30.2%)
ΔEorb(ρ_1)[b]	–44.4 (66.7%)	–60.7 (65.6%)	–38.4 (50.0%)
ΔEorb(ρ_2)[b]	–7.4 (8.7%)	–7.3 (7.9%)	–19.2 (24.9%)
ΔEorb(ρ_3)[b]	–7.2 (8.5%)	-	-
ΔEorb(rest)[b]	–23.1 (27.1%)	–24.6 (26.5%)	–19.2 (25.1%)
ΔEdisp[a]	–3.0 (1.2%)	–10.8 (2.7%)	–10.5 (4.1%)

[a] The values within parentheses indicate the percentage to the total attractive interactions, ΔEint = ΔEelstat + ΔEorb + ΔEdisp. [b] The values within parentheses indicate the percentage to the total orbital interactions (ΔEorb).

4.2 Cartesian coordinates and energies for all the species discussed in the text

3 E(RPBE1PBE) = -2905.95055794

Au	-1.489542000	-0.061857000	-0.007280000
C	3.296295000	-3.211661000	-0.463334000
C	3.355231000	1.230428000	2.902381000
C	0.927869000	-2.646848000	1.010247000
C	-0.277239000	-2.385014000	1.831214000
C	-2.629301000	-1.779472000	3.294035000
C	-0.318786000	-0.387805000	-3.000573000
C	0.907553000	0.425194000	-2.829098000
C	3.268726000	1.994850000	-2.583470000
C	-3.887075000	-1.378843000	4.062856000
C	-2.721261000	-1.883582000	-3.156292000
C	4.589382000	-3.523051000	-1.212817000
C	4.537888000	2.821028000	-2.383144000
C	-3.955847000	4.199537000	-0.734355000
C	-2.644236000	3.735823000	-0.103487000
C	0.956546000	2.210679000	1.724852000
C	-0.267183000	2.776425000	1.108329000
C	5.655589000	-3.972644000	-0.202717000
H	6.594511000	-4.199011000	-0.720271000
H	5.854021000	-3.185987000	0.533774000
H	5.347757000	-4.872128000	0.339568000
C	5.124595000	-2.305235000	-1.968758000
H	4.421009000	-1.951195000	-2.730202000
H	5.343473000	-1.472118000	-1.291694000
H	6.056315000	-2.572770000	-2.477654000
C	4.330083000	-4.651959000	-2.220445000
H	3.571895000	-4.355113000	-2.953226000
H	5.251313000	-4.894568000	-2.761992000
H	3.982689000	-5.564225000	-1.725122000

C	4.233848000	3.984007000	-1.426078000
H	3.901513000	3.628004000	-0.445329000
H	3.450106000	4.632032000	-1.833341000
H	5.132823000	4.591761000	-1.273613000
C	5.061506000	3.394462000	-3.699596000
H	5.970005000	3.975527000	-3.510537000
H	5.315202000	2.604225000	-4.414393000
H	4.332073000	4.062363000	-4.170139000
C	5.627516000	1.930220000	-1.766934000
H	5.317261000	1.525028000	-0.797992000
H	6.543704000	2.509964000	-1.607924000
H	5.867312000	1.088740000	-2.426210000
C	2.682127000	-1.961913000	-0.524615000
H	3.112930000	-1.187825000	-1.148128000
C	1.513771000	-1.665981000	0.188831000
C	1.520089000	-3.914671000	1.037418000
C	2.675804000	-4.192573000	0.320940000
C	2.662731000	1.402669000	-1.471670000
H	3.102144000	1.559290000	-0.492520000
C	2.655027000	1.803006000	-3.822685000
C	1.498372000	1.035510000	-3.936859000
C	1.501927000	0.633906000	-1.567231000
C	-0.327783000	-1.759445000	-2.721719000
C	-1.507335000	-2.488229000	-2.802259000
H	-1.479838000	-3.548832000	-2.567193000
C	-2.692341000	-0.523162000	-3.476293000
C	-1.512094000	0.212858000	-3.407072000
C	-1.472812000	-3.061169000	1.578513000
H	-1.510322000	-3.802384000	0.784547000
C	-2.628444000	-2.756342000	2.294675000
H	-3.543925000	-3.280005000	2.042306000
C	-1.411808000	-1.143931000	3.574893000
H	-1.362258000	-0.392628000	4.358521000

C	-0.257385000	-1.436555000	2.860783000
H	0.667016000	-0.916643000	3.094684000
C	2.751200000	2.410315000	3.356325000
C	2.712733000	0.539677000	1.876985000
C	1.579854000	2.884752000	2.781680000
C	1.529971000	1.003886000	1.287907000
C	-0.269765000	3.215961000	-0.216845000
H	0.648198000	3.183545000	-0.796007000
C	-1.439400000	3.687144000	-0.809293000
H	-1.398102000	4.009989000	-1.844109000
C	-2.622867000	3.323092000	1.236961000
C	-1.459835000	2.858558000	1.836443000
H	-1.479420000	2.519445000	2.868700000
C	-4.208321000	-3.348848000	-1.790103000
H	-5.135379000	-3.934039000	-1.778163000
H	-4.274035000	-2.577464000	-1.016626000
H	-3.386513000	-4.022885000	-1.528698000
C	-5.234712000	-1.860594000	-3.491934000
H	-6.129937000	-2.491092000	-3.472137000
H	-5.172463000	-1.406615000	-4.486937000
H	-5.371707000	-1.061620000	-2.755311000
C	-3.681774000	-1.660335000	5.558653000
H	-3.504536000	-2.726743000	5.736978000
H	-2.829348000	-1.104690000	5.962487000
H	-4.572474000	-1.364776000	6.124965000
C	-5.122419000	-2.144740000	3.588466000
H	-5.327492000	-1.962013000	2.528295000
H	-5.016886000	-3.224684000	3.739115000
H	-5.997441000	-1.815362000	4.158758000
C	-4.142691000	0.121924000	3.856267000
H	-3.308199000	0.728692000	4.221264000
H	-4.286675000	0.348391000	2.795041000
H	-5.043213000	0.429339000	4.400848000

C	-3.774866000	4.635765000	-2.188650000
H	-3.410601000	3.814273000	-2.815123000
H	-4.738414000	4.960340000	-2.595490000
H	-3.076382000	5.474886000	-2.279717000
C	-4.516610000	5.386726000	0.062662000
H	-4.710929000	5.120359000	1.106319000
H	-3.819245000	6.231747000	0.054205000
H	-5.462917000	5.722505000	-0.376636000
C	4.446089000	0.474607000	5.017344000
H	5.379039000	0.132929000	5.479511000
H	4.121839000	1.376930000	5.545365000
H	3.686167000	-0.299088000	5.172066000
H	1.068801000	-4.687079000	1.654577000
H	3.104553000	-5.188905000	0.384546000
H	3.205346000	2.976184000	4.165235000
H	1.140017000	3.811626000	3.140454000
H	3.134729000	-0.392128000	1.521186000
C	4.662348000	0.739179000	3.520537000
C	5.736197000	1.822510000	3.341137000
H	5.909151000	2.030361000	2.279280000
H	5.450088000	2.761177000	3.825835000
H	6.683520000	1.492468000	3.781878000
C	5.161877000	-0.550447000	2.864835000
H	4.446821000	-1.372661000	2.978492000
H	5.358263000	-0.412833000	1.795508000
H	6.100030000	-0.858982000	3.337244000
H	-3.537959000	3.343365000	1.822256000
C	-4.004594000	-2.711342000	-3.173121000
C	-3.880292000	-3.814838000	-4.233971000
H	-3.032517000	-4.476711000	-4.029479000
H	-4.788260000	-4.428635000	-4.252702000
H	-3.739878000	-3.385614000	-5.232273000
H	1.044076000	0.892347000	-4.913792000

H	3.074310000	2.248657000	-4.718604000
H	0.593216000	-2.254527000	-2.427735000
H	-3.604795000	-0.009856000	-3.759390000
H	-1.526183000	1.277307000	-3.626717000
C	-4.963384000	3.040561000	-0.700002000
H	-4.595397000	2.186993000	-1.277916000
H	-5.149728000	2.695565000	0.321422000
H	-5.920164000	3.361225000	-1.128967000
P	0.748910000	-0.011766000	-0.023390000
F	-3.509027000	-0.163788000	-0.001523000

B(C6F5)3 E(RPBE1PBE) = -2206.08903751

B	0.004033000	0.002687000	-0.001039000
C	1.517654000	0.389843000	-0.001812000
C	2.000675000	1.509706000	0.682535000
C	2.478211000	-0.362238000	-0.685374000
C	3.341042000	1.860218000	0.701754000
C	3.823156000	-0.030230000	-0.702619000
C	4.255747000	1.086427000	0.000013000
C	-1.090865000	1.117895000	0.002975000
C	-2.293612000	0.976594000	0.701466000
C	-0.932052000	2.319165000	-0.694048000
C	-3.272918000	1.956608000	0.720183000
C	-1.898321000	3.311862000	-0.712884000
C	-3.073790000	3.128837000	0.003044000
C	-0.419325000	-1.502029000	-0.002936000
C	-1.543183000	-1.957040000	-0.699036000
C	0.298932000	-2.478651000	0.693216000
C	-1.928867000	-3.287700000	-0.718095000
C	-0.069266000	-3.814181000	0.711714000
C	-1.188531000	-4.219615000	-0.003050000
F	5.539381000	1.412539000	0.001498000
F	3.757275000	2.925740000	1.380446000
F	1.169937000	2.286506000	1.379992000

F	2.121211000	-1.441889000	-1.382983000
F	4.698702000	-0.767500000	-1.380212000
F	1.379632000	-2.145590000	1.401388000
F	0.635895000	-4.706615000	1.401301000
F	-1.548971000	-5.494164000	-0.003523000
F	-2.996572000	-3.678406000	-1.408540000
F	-2.289580000	-1.107492000	-1.407330000
F	-2.536867000	-0.126085000	1.412374000
F	-4.396210000	1.785021000	1.411639000
F	-4.003649000	4.072196000	0.002815000
F	-1.710852000	4.432956000	-1.403831000
F	0.175304000	2.547642000	-1.402486000

$F(B(C6F5)3)^-$ E(RPBE1PBE) = -2306.03120927

B	-0.083154000	0.061351000	0.912245000
C	-1.611376000	0.483712000	0.477941000
C	-2.014929000	1.668563000	-0.123614000
C	-2.654563000	-0.368802000	0.832200000
C	-3.345995000	1.986455000	-0.378183000
C	-3.993769000	-0.095279000	0.600409000
C	-4.343510000	1.099335000	-0.012838000
C	1.071367000	1.099416000	0.375938000
C	1.935026000	1.817234000	1.194086000
C	1.307436000	1.240513000	-0.988005000
C	2.963071000	2.615838000	0.699088000
C	2.315691000	2.024150000	-1.525252000
C	3.156928000	2.719452000	-0.667709000
C	0.362647000	-1.420112000	0.334127000
C	1.451816000	-2.062955000	0.916783000
C	-0.210369000	-2.103702000	-0.732026000
C	1.931050000	-3.299408000	0.504042000
C	0.236039000	-3.341523000	-1.179807000
C	1.313822000	-3.947349000	-0.554657000
F	-0.084785000	0.004468000	2.331833000

F	1.824423000	1.788088000	2.530485000
F	3.765267000	3.285970000	1.533254000
F	4.136911000	3.481751000	-1.156353000
F	2.485765000	2.121622000	-2.847801000
F	0.512459000	0.602862000	-1.865174000
F	-1.251876000	-1.591240000	-1.407161000
F	2.123605000	-1.481690000	1.923640000
F	-0.362699000	-3.952396000	-2.207610000
F	1.755942000	-5.135313000	-0.970183000
F	2.981776000	-3.865045000	1.107066000
F	-1.126928000	2.605055000	-0.497014000
F	-3.669427000	3.144722000	-0.962173000
F	-5.624480000	1.388621000	-0.244856000
F	-4.947117000	-0.961638000	0.957203000
F	-2.386228000	-1.546946000	1.419693000

4 E(RPBE1PBE) = -3143.50685454

Au	-1.426128000	0.098393000	0.081499000
C	3.590087000	2.863213000	-1.194316000
C	3.343587000	-2.647184000	-1.976257000
C	1.159546000	1.758098000	-2.179573000
C	-0.063818000	1.158763000	-2.762086000
C	-2.421170000	-0.034584000	-3.791769000
C	0.075437000	1.996285000	2.341712000
C	1.200997000	1.072150000	2.622572000
C	3.405969000	-0.620107000	3.236473000
C	-3.677425000	-0.717793000	-4.330293000
C	-2.106859000	3.716955000	1.769735000
C	4.911813000	3.445452000	-0.699343000
C	4.600962000	-1.535874000	3.493062000
C	-3.921329000	-3.663780000	2.686256000
C	-2.656483000	-3.392480000	1.871793000
C	0.921116000	-2.811732000	-0.481498000
C	-0.308535000	-2.985677000	0.323718000

C	5.970956000	3.285319000	-1.799838000
H	6.927616000	3.703988000	-1.467889000
H	6.127296000	2.227814000	-2.040723000
H	5.680375000	3.801388000	-2.720219000
C	5.414843000	2.740945000	0.562549000
H	4.710326000	2.838448000	1.395960000
H	5.593079000	1.673989000	0.388205000
H	6.363552000	3.188533000	0.875993000
C	4.719614000	4.935811000	-0.384151000
H	3.965962000	5.075781000	0.398553000
H	5.661008000	5.372671000	-0.032473000
H	4.398746000	5.498538000	-1.266533000
C	4.173771000	-2.996979000	3.285928000
H	3.824732000	-3.179686000	2.264314000
H	3.365514000	-3.270154000	3.972924000
H	5.020716000	-3.666670000	3.473155000
C	5.146112000	-1.382748000	4.912694000
H	6.001654000	-2.051959000	5.050140000
H	5.488585000	-0.360920000	5.108460000
H	4.395669000	-1.644244000	5.666460000
C	5.718870000	-1.188053000	2.498031000
H	5.392649000	-1.320983000	1.461059000
H	6.586099000	-1.837776000	2.661274000
H	6.044101000	-0.149053000	2.620638000
C	2.940664000	1.825048000	-0.529799000
H	3.367755000	1.430608000	0.384247000
C	1.738047000	1.276862000	-0.992696000
C	1.789856000	2.827757000	-2.826326000
C	2.974365000	3.370006000	-2.346600000
C	2.785826000	-0.649211000	1.983434000
H	3.154368000	-1.339373000	1.232058000
C	2.882455000	0.257016000	4.187697000
C	1.802738000	1.082727000	3.882142000

C	1.700752000	0.166987000	1.663170000
C	0.203013000	3.032864000	1.409788000
C	-0.868061000	3.872647000	1.133397000
H	-0.730032000	4.666193000	0.404270000
C	-2.212834000	2.703941000	2.727067000
C	-1.142233000	1.859403000	3.012003000
C	-1.247594000	1.890888000	-2.873341000
H	-1.274527000	2.920994000	-2.528118000
C	-2.405006000	1.300139000	-3.375350000
H	-3.311313000	1.894165000	-3.412102000
C	-1.216571000	-0.746513000	-3.717240000
H	-1.175390000	-1.778321000	-4.054443000
C	-0.060586000	-0.167070000	-3.211525000
H	0.855741000	-0.747359000	-3.164726000
C	2.645577000	-3.855992000	-1.853027000
C	2.782447000	-1.527347000	-1.368005000
C	1.467951000	-3.931816000	-1.124230000
C	1.589393000	-1.583389000	-0.635268000
C	-0.358203000	-2.614553000	1.670064000
H	0.519575000	-2.185262000	2.143538000
C	-1.512060000	-2.812245000	2.425571000
H	-1.501126000	-2.508047000	3.466595000
C	-2.595945000	-3.766671000	0.521326000
C	-1.450004000	-3.572153000	-0.238131000
H	-1.441394000	-3.857962000	-1.286687000
C	-3.459877000	4.697571000	-0.098856000
H	-4.304614000	5.349972000	-0.347804000
H	-3.667784000	3.698206000	-0.491668000
H	-2.577802000	5.089791000	-0.614318000
C	-4.579182000	4.193886000	2.057240000
H	-5.387395000	4.866135000	1.750211000
H	-4.536613000	4.211371000	3.151453000
H	-4.843723000	3.180281000	1.738022000

C	-3.498570000	-0.968586000	-5.834824000
H	-3.357936000	-0.026933000	-6.376866000
H	-2.629119000	-1.604670000	-6.031798000
H	-4.382989000	-1.469330000	-6.245815000
C	-4.928402000	0.135299000	-4.114257000
H	-5.078906000	0.359244000	-3.052992000
H	-4.882420000	1.081590000	-4.663713000
H	-5.808720000	-0.408755000	-4.472531000
C	-3.887644000	-2.059775000	-3.613246000
H	-3.064750000	-2.757511000	-3.796554000
H	-3.974119000	-1.911361000	-2.533434000
H	-4.807565000	-2.534096000	-3.973744000
C	-3.829365000	-3.086034000	4.099079000
H	-3.673906000	-2.001750000	4.080130000
H	-4.764339000	-3.280246000	4.635120000
H	-3.017656000	-3.540469000	4.677266000
C	-4.119740000	-5.184270000	2.788360000
H	-4.236214000	-5.640000000	1.799567000
H	-3.265293000	-5.660937000	3.281386000
H	-5.019782000	-5.411917000	3.371267000
C	4.399194000	-2.993136000	-4.210452000
H	5.331571000	-2.953123000	-4.784703000
H	4.008380000	-4.013456000	-4.276142000
H	3.676125000	-2.322160000	-4.686867000
H	1.345848000	3.221783000	-3.736703000
H	3.431469000	4.192967000	-2.889394000
H	3.030488000	-4.756948000	-2.322844000
H	0.959877000	-4.887159000	-1.023143000
H	3.272644000	-0.567821000	-1.473651000
C	4.655339000	-2.577735000	-2.754675000
C	5.666876000	-3.541852000	-2.117598000
H	5.864149000	-3.270101000	-1.074634000
H	5.307795000	-4.575778000	-2.134807000

H	6.616193000	-3.508166000	-2.663844000
C	5.256501000	-1.170346000	-2.746883000
H	4.591823000	-0.437005000	-3.216613000
H	5.478324000	-0.828864000	-1.729511000
H	6.196619000	-1.173280000	-3.307970000
H	-3.462803000	-4.218577000	0.047211000
C	-3.264683000	4.652216000	1.423785000
C	-2.931081000	6.061727000	1.934636000
H	-2.011144000	6.443985000	1.479645000
H	-3.742405000	6.757651000	1.691194000
H	-2.795854000	6.063487000	3.021824000
H	1.422725000	1.768360000	4.634974000
H	3.315290000	0.313615000	5.180987000
H	1.148238000	3.184044000	0.898553000
H	-3.149050000	2.541928000	3.249190000
H	-1.263920000	1.062538000	3.741426000
C	-5.142642000	-3.041899000	1.994455000
H	-5.035834000	-1.957573000	1.911019000
H	-5.292978000	-3.444632000	0.988251000
H	-6.046428000	-3.257280000	2.576002000
P	0.909046000	-0.009881000	0.017335000
C	-3.480646000	0.188622000	0.167622000
F	-3.999334000	0.246238000	1.442020000
F	-4.048640000	1.273957000	-0.457159000
F	-4.132820000	-0.879447000	-0.395802000

4·B(C6F5)3 E(RPBE1PBE) = -5349.62747017

Au	-0.311309000	-0.212768000	0.954162000
C	-3.228396000	-0.889347000	-3.808440000
C	-5.201336000	2.865692000	0.538071000
C	-2.138236000	1.217325000	-2.236220000
C	-1.562241000	2.352100000	-1.468650000
C	-0.558903000	4.441468000	0.164351000
C	-1.758417000	-3.470628000	0.334284000

C	-3.173109000	-3.034509000	0.416808000
C	-5.958789000	-2.448726000	0.567003000
C	-0.073388000	5.559728000	1.084024000
C	0.867275000	-4.545014000	0.245183000
C	-3.772370000	-2.029800000	-4.666015000
C	-7.434714000	-2.061922000	0.634090000
C	0.338379000	-1.059980000	5.955608000
C	-0.639433000	-0.383407000	4.996452000
C	-3.282459000	1.598651000	2.215821000
C	-2.377489000	0.922460000	3.171237000
C	-4.934289000	-1.518765000	-5.528776000
H	-5.324157000	-2.329945000	-6.153878000
H	-5.753635000	-1.148447000	-4.902712000
H	-4.624217000	-0.707306000	-6.194402000
C	-4.280043000	-3.197399000	-3.817966000
H	-3.477953000	-3.649615000	-3.224920000
H	-5.078263000	-2.889528000	-3.133220000
H	-4.684549000	-3.975665000	-4.473220000
C	-2.641972000	-2.539920000	-5.572429000
H	-1.788976000	-2.879314000	-4.975572000
H	-2.993802000	-3.380467000	-6.181499000
H	-2.287633000	-1.757579000	-6.250887000
C	-7.673577000	-1.178395000	1.867567000
H	-7.076687000	-0.261188000	1.834643000
H	-7.418408000	-1.713043000	2.788893000
H	-8.728683000	-0.887717000	1.923591000
C	-8.344268000	-3.286863000	0.727596000
H	-9.388787000	-2.963374000	0.782439000
H	-8.244204000	-3.936531000	-0.148587000
H	-8.132320000	-3.881642000	1.622662000
C	-7.799825000	-1.273501000	-0.633083000
H	-7.196279000	-0.365331000	-0.729274000
H	-8.853841000	-0.975066000	-0.602907000

H	-7.642435000	-1.879640000	-1.532069000
C	-3.126441000	-0.992645000	-2.421253000
H	-3.475407000	-1.894488000	-1.935705000
C	-2.599658000	0.030522000	-1.626403000
C	-2.267303000	1.329802000	-3.623893000
C	-2.794121000	0.302309000	-4.396506000
C	-4.992768000	-1.440878000	0.518195000
H	-5.327183000	-0.410468000	0.528392000
C	-5.503633000	-3.766588000	0.529288000
C	-4.142674000	-4.043301000	0.456138000
C	-3.621308000	-1.696796000	0.437444000
C	-1.342358000	-4.268323000	-0.737265000
C	-0.055891000	-4.789746000	-0.780145000
H	0.225818000	-5.408824000	-1.626978000
C	0.443085000	-3.749421000	1.313619000
C	-0.844706000	-3.218431000	1.360723000
C	-0.299103000	2.264040000	-0.886439000
H	0.318194000	1.390341000	-1.068846000
C	0.186714000	3.287416000	-0.073440000
H	1.160841000	3.159625000	0.381341000
C	-1.791599000	4.559960000	-0.496000000
H	-2.391370000	5.456968000	-0.366262000
C	-2.283439000	3.539278000	-1.297272000
H	-3.254402000	3.646512000	-1.771916000
C	-4.935710000	3.342462000	1.828278000
C	-4.451941000	1.777913000	0.096100000
C	-4.000401000	2.723567000	2.643320000
C	-3.495092000	1.145624000	0.901690000
C	-2.545952000	-0.428948000	3.484628000
H	-3.358669000	-0.988004000	3.029613000
C	-1.690855000	-1.067488000	4.380430000
H	-1.856335000	-2.119113000	4.589434000
C	-0.503180000	0.981635000	4.705034000

C	-1.352929000	1.625859000	3.816634000
H	-1.202120000	2.677476000	3.587167000
C	2.888121000	-4.987130000	-1.188095000
H	3.896302000	-5.412367000	-1.200452000
H	2.962243000	-3.927055000	-1.442087000
H	2.313071000	-5.483655000	-1.975466000
C	3.191505000	-4.576026000	1.244885000
H	4.190714000	-5.011388000	1.141922000
H	2.852201000	-4.780964000	2.265673000
H	3.276059000	-3.491221000	1.125637000
C	0.136229000	6.836166000	0.256250000
H	0.892042000	6.675575000	-0.520413000
H	-0.789352000	7.156469000	-0.232981000
H	0.477723000	7.655702000	0.899233000
C	1.246636000	5.206624000	1.771660000
H	1.164337000	4.289045000	2.364575000
H	2.057228000	5.077010000	1.047847000
H	1.534375000	6.018193000	2.448419000
C	-1.130646000	5.817959000	2.167106000
H	-2.093047000	6.112696000	1.737280000
H	-1.296286000	4.918922000	2.770648000
H	-0.800792000	6.622091000	2.834914000
C	0.063663000	-2.557588000	6.097780000
H	0.150170000	-3.077572000	5.137174000
H	0.795467000	-2.999942000	6.781791000
H	-0.932993000	-2.755519000	6.506876000
C	0.219881000	-0.406009000	7.340120000
H	0.446694000	0.664155000	7.300387000
H	-0.791799000	-0.522123000	7.744198000
H	0.922141000	-0.871916000	8.040855000
C	-5.891345000	5.011741000	-0.544432000
H	-6.659538000	5.510066000	-1.146532000
H	-5.807139000	5.552340000	0.403461000

H	-4.936263000	5.102540000	-1.072389000
H	-1.928203000	2.244647000	-4.102917000
H	-2.853929000	0.434027000	-5.473175000
H	-5.477544000	4.202315000	2.212340000
H	-3.834930000	3.093605000	3.651570000
H	-4.597619000	1.407786000	-0.912363000
C	-6.264413000	3.537667000	-0.329092000
C	-7.618369000	3.457499000	0.391810000
H	-7.911097000	2.415882000	0.563786000
H	-7.590800000	3.964619000	1.361497000
H	-8.397758000	3.934096000	-0.213449000
C	-6.403308000	2.867717000	-1.696779000
H	-5.472231000	2.911718000	-2.272404000
H	-6.700456000	1.816898000	-1.608227000
H	-7.175869000	3.382138000	-2.277470000
H	0.297307000	1.553390000	5.166010000
C	2.255742000	-5.181334000	0.197099000
C	2.110483000	-6.685606000	0.472497000
H	1.467743000	-7.165393000	-0.273247000
H	3.090690000	-7.175856000	0.440549000
H	1.672317000	-6.863711000	1.460657000
H	-3.809134000	-5.077337000	0.441551000
H	-6.202861000	-4.595371000	0.562125000
H	-2.039445000	-4.484245000	-1.543042000
H	1.117483000	-3.527978000	2.133002000
H	-1.150227000	-2.633508000	2.223079000
C	1.771362000	-0.883030000	5.431759000
H	1.888467000	-1.333186000	4.441428000
H	2.049377000	0.171964000	5.353192000
H	2.480776000	-1.365565000	6.113929000
P	-2.519921000	-0.238285000	0.194975000
C	1.650341000	0.127106000	1.491765000
F	2.396734000	-0.957943000	1.863756000

F	2.389309000	0.702995000	0.466107000
F	1.820859000	1.014244000	2.518807000
B	4.267343000	0.322023000	-1.337814000
C	4.760084000	1.792609000	-1.125464000
C	3.905182000	2.890600000	-1.267433000
C	6.066011000	2.100892000	-0.729677000
C	4.293312000	4.193373000	-1.002354000
C	6.494622000	3.394682000	-0.477325000
C	5.598160000	4.446700000	-0.603973000
C	3.147503000	-0.000997000	-2.383807000
C	2.168813000	-0.970348000	-2.146391000
C	3.033235000	0.688161000	-3.593043000
C	1.124455000	-1.218499000	-3.023698000
C	2.017976000	0.448974000	-4.504408000
C	1.055500000	-0.508783000	-4.214376000
C	4.937971000	-0.841515000	-0.533186000
C	5.243196000	-2.066207000	-1.128444000
C	5.274437000	-0.719823000	0.816912000
C	5.866752000	-3.097964000	-0.445267000
C	5.864502000	-1.744173000	1.539289000
C	6.167398000	-2.937618000	0.898904000
F	5.988953000	5.687463000	-0.353784000
F	3.427495000	5.199185000	-1.120325000
F	2.642440000	2.721357000	-1.655685000
F	6.982221000	1.140499000	-0.594063000
F	7.750969000	3.634903000	-0.110266000
F	5.022297000	0.410543000	1.476818000
F	6.140475000	-1.595855000	2.832791000
F	6.717877000	-3.936925000	1.575685000
F	6.144606000	-4.251948000	-1.050088000
F	4.952224000	-2.284522000	-2.414191000
F	2.188664000	-1.691048000	-1.024607000
F	0.193478000	-2.124688000	-2.736106000

F	0.080816000	-0.748433000	-5.079767000
F	1.955473000	1.124073000	-5.650094000
F	3.930505000	1.619976000	-3.924855000

TS1 E(RPBE1PBE) = -5349.61778118

Au	-0.222015000	-0.201712000	0.877265000
C	-3.247569000	-1.107647000	-3.747156000
C	-5.306500000	2.632843000	0.619023000
C	-2.286878000	1.127225000	-2.269757000
C	-1.810115000	2.331923000	-1.546160000
C	-1.020383000	4.564063000	0.014627000
C	-1.492090000	-3.493931000	0.457787000
C	-2.925129000	-3.119198000	0.544921000
C	-5.736155000	-2.685093000	0.741291000
C	-0.648185000	5.756827000	0.892397000
C	1.157479000	-4.511542000	0.389690000
C	-3.733369000	-2.307314000	-4.557314000
C	-7.229403000	-2.378240000	0.828005000
C	0.680067000	-0.766773000	5.913664000
C	-0.355006000	-0.182875000	4.953016000
C	-3.218424000	1.545508000	2.220056000
C	-2.227313000	0.954683000	3.146905000
C	-4.995441000	-1.919321000	-5.340347000
H	-5.347132000	-2.770882000	-5.933512000
H	-5.801702000	-1.622206000	-4.660599000
H	-4.809238000	-1.088931000	-6.028327000
C	-4.066693000	-3.507106000	-3.668879000
H	-3.187635000	-3.864912000	-3.122383000
H	-4.850686000	-3.272379000	-2.940159000
H	-4.427527000	-4.332245000	-4.291433000
C	-2.624037000	-2.718277000	-5.537139000
H	-1.704455000	-2.969255000	-4.999142000
H	-2.936032000	-3.594434000	-6.116931000
H	-2.391921000	-1.913738000	-6.241902000

C	-7.494559000	-1.474125000	2.040760000
H	-6.946964000	-0.528537000	1.972140000
H	-7.197381000	-1.968586000	2.971890000
H	-8.562432000	-1.237300000	2.106712000
C	-8.067756000	-3.648070000	0.971911000
H	-9.127502000	-3.380797000	1.036071000
H	-7.946157000	-4.315763000	0.112242000
H	-7.809690000	-4.205159000	1.879078000
C	-7.659329000	-1.647584000	-0.452990000
H	-7.111075000	-0.709050000	-0.582887000
H	-8.728235000	-1.409586000	-0.412897000
H	-7.480312000	-2.267487000	-1.338440000
C	-3.075017000	-1.168727000	-2.364770000
H	-3.317447000	-2.085755000	-1.844513000
C	-2.611206000	-0.081407000	-1.617194000
C	-2.484287000	1.193374000	-3.652555000
C	-2.948553000	0.103901000	-4.378023000
C	-4.829683000	-1.627464000	0.641824000
H	-5.221430000	-0.617805000	0.634376000
C	-5.208817000	-3.976208000	0.729899000
C	-3.836320000	-4.178215000	0.636040000
C	-3.447944000	-1.809109000	0.533045000
C	-1.047725000	-4.276576000	-0.612539000
C	0.250506000	-4.768995000	-0.645934000
H	0.553331000	-5.374767000	-1.494576000
C	0.704390000	-3.732700000	1.459163000
C	-0.593502000	-3.226312000	1.494471000
C	-0.570132000	2.360381000	-0.911273000
H	0.116165000	1.527856000	-1.037222000
C	-0.193867000	3.449636000	-0.126692000
H	0.768477000	3.416901000	0.366632000
C	-2.230581000	4.562007000	-0.694341000
H	-2.896203000	5.418695000	-0.631504000

C	-2.618705000	3.471333000	-1.459098000
H	-3.575261000	3.487019000	-1.973383000
C	-5.013871000	3.154384000	1.885920000
C	-4.500985000	1.594494000	0.158094000
C	-3.994906000	2.624921000	2.662455000
C	-3.463857000	1.049099000	0.928075000
C	-2.316022000	-0.387520000	3.525680000
H	-3.120761000	-1.003234000	3.133390000
C	-1.395616000	-0.942973000	4.412480000
H	-1.505113000	-1.988330000	4.680630000
C	-0.291943000	1.170785000	4.589365000
C	-1.208279000	1.733315000	3.710551000
H	-1.123944000	2.781358000	3.434963000
C	3.164508000	-4.972133000	-1.056214000
H	4.200366000	-5.324928000	-1.054882000
H	3.159647000	-3.931516000	-1.390868000
H	2.619858000	-5.565371000	-1.796677000
C	3.499231000	-4.419224000	1.343372000
H	4.510004000	-4.828401000	1.243910000
H	3.187314000	-4.575890000	2.381276000
H	3.549139000	-3.341899000	1.158652000
C	-0.639872000	7.035717000	0.042723000
H	0.093241000	6.960375000	-0.767700000
H	-1.619196000	7.234519000	-0.403924000
H	-0.374390000	7.899626000	0.662601000
C	0.733139000	5.594464000	1.529929000
H	0.783668000	4.710838000	2.175799000
H	1.522628000	5.513043000	0.775230000
H	0.954177000	6.469849000	2.149806000
C	-1.691633000	5.893144000	2.011047000
H	-2.699697000	6.034841000	1.608139000
H	-1.707357000	4.997082000	2.641085000
H	-1.456701000	6.754272000	2.647262000

C	0.463863000	-2.261258000	6.155620000
H	0.534216000	-2.836715000	5.225817000
H	1.234999000	-2.636075000	6.836549000
H	-0.509759000	-2.466310000	6.613431000
C	0.584860000	-0.034763000	7.260787000
H	0.777748000	1.037332000	7.152928000
H	-0.409461000	-0.156012000	7.704343000
H	1.322657000	-0.438587000	7.963438000
C	-6.209158000	4.701708000	-0.454603000
H	-7.044583000	5.133118000	-1.017339000
H	-6.111160000	5.261291000	0.480840000
H	-5.294183000	4.852435000	-1.036249000
H	-2.251754000	2.123243000	-4.164868000
H	-3.070074000	0.203143000	-5.452967000
H	-5.598045000	3.980407000	2.281839000
H	-3.807987000	3.027751000	3.654167000
H	-4.667494000	1.193575000	-0.835567000
C	-6.459428000	3.207882000	-0.201921000
C	-7.766135000	3.039111000	0.587678000
H	-7.973197000	1.981499000	0.785109000
H	-7.728940000	3.560802000	1.549248000
H	-8.607064000	3.449121000	0.017057000
C	-6.615815000	2.507094000	-1.551982000
H	-5.718497000	2.604635000	-2.172310000
H	-6.834856000	1.440192000	-1.435903000
H	-7.448578000	2.957628000	-2.101571000
H	0.496903000	1.799876000	4.992598000
C	2.563071000	-5.109447000	0.349227000
C	2.468252000	-6.600026000	0.708506000
H	1.822785000	-7.136821000	0.004970000
H	3.461526000	-7.063433000	0.679039000
H	2.058346000	-6.736692000	1.715249000
H	-3.445550000	-5.191963000	0.645076000

H	-5.859985000	-4.841196000	0.799357000
H	-1.732805000	-4.507875000	-1.424001000
H	1.366228000	-3.504627000	2.287072000
H	-0.920308000	-2.650595000	2.355266000
C	2.087842000	-0.574217000	5.331076000
H	2.194822000	-1.100440000	4.377510000
H	2.318662000	0.481496000	5.161457000
H	2.836862000	-0.973039000	6.024548000
P	-2.450393000	-0.296061000	0.204620000
C	1.727652000	0.056277000	1.462058000
F	2.483991000	-0.989590000	1.744206000
F	2.592220000	0.743990000	0.238009000
F	2.002802000	0.979434000	2.368600000
B	3.733881000	0.449121000	-0.951274000
C	4.474786000	1.882265000	-0.961787000
C	3.728134000	3.055761000	-1.049033000
C	5.848549000	2.076121000	-0.835922000
C	4.269001000	4.327709000	-0.965965000
C	6.434625000	3.335298000	-0.760959000
C	5.640707000	4.469018000	-0.813641000
C	2.767808000	0.114756000	-2.192824000
C	1.854957000	-0.934713000	-2.096113000
C	2.696886000	0.846314000	-3.374470000
C	0.907668000	-1.225892000	-3.064593000
C	1.768864000	0.582380000	-4.375279000
C	0.866430000	-0.456802000	-4.219055000
C	4.646618000	-0.746600000	-0.392318000
C	5.053001000	-1.836150000	-1.153295000
C	5.177491000	-0.690670000	0.894613000
C	5.920229000	-2.811724000	-0.676861000
C	6.026780000	-1.652642000	1.415574000
C	6.401584000	-2.724757000	0.618040000
F	6.187996000	5.677833000	-0.735507000

F	3.488536000	5.408310000	-1.037752000
F	2.407143000	2.986952000	-1.246521000
F	6.701592000	1.044816000	-0.792678000
F	7.756570000	3.457362000	-0.634467000
F	4.882807000	0.346010000	1.690836000
F	6.487127000	-1.557613000	2.663588000
F	7.193623000	-3.677473000	1.102477000
F	6.267412000	-3.846399000	-1.444464000
F	4.622016000	-1.998642000	-2.411929000
F	1.869547000	-1.726280000	-1.014044000
F	0.044339000	-2.229444000	-2.901288000
F	-0.019486000	-0.721446000	-5.174023000
F	1.742413000	1.320492000	-5.485563000
F	3.537696000	1.859232000	-3.616730000

5 E(RPBE1PBE) = -5349.62569801

Au	-0.465532000	-0.402247000	1.133476000
C	-2.963605000	-0.567131000	-3.949218000
C	-5.021785000	3.107004000	0.357193000
C	-1.804871000	1.328503000	-2.171373000
C	-1.200999000	2.348368000	-1.277286000
C	-0.129215000	4.214434000	0.570127000
C	-1.930163000	-3.390792000	0.121452000
C	-3.336457000	-2.916225000	0.207278000
C	-6.099731000	-2.221278000	0.297733000
C	0.429689000	5.234932000	1.558463000
C	0.673757000	-4.510710000	-0.047898000
C	-3.562067000	-1.581430000	-4.920853000
C	-7.561715000	-1.780389000	0.321290000
C	-0.158647000	-1.245872000	6.097191000
C	-1.007644000	-0.507859000	5.063327000
C	-3.334216000	1.667284000	2.143755000
C	-2.533770000	0.920693000	3.139884000
C	-4.594997000	-0.882718000	-5.816085000

H	-5.024048000	-1.598131000	-6.526693000
H	-5.412479000	-0.465617000	-5.217658000
H	-4.147601000	-0.067724000	-6.393039000
C	-4.256247000	-2.736053000	-4.196021000
H	-3.556725000	-3.312015000	-3.580858000
H	-5.069754000	-2.386053000	-3.550350000
H	-4.688045000	-3.420987000	-4.932762000
C	-2.431774000	-2.154939000	-5.788997000
H	-1.657422000	-2.615979000	-5.167924000
H	-2.827684000	-2.915780000	-6.471100000
H	-1.955242000	-1.377063000	-6.393105000
C	-7.805703000	-0.876707000	1.539054000
H	-7.188897000	0.027485000	1.508840000
H	-7.584643000	-1.405747000	2.472324000
H	-8.854572000	-0.561163000	1.567023000
C	-8.517169000	-2.970960000	0.397634000
H	-9.550090000	-2.609343000	0.424855000
H	-8.419629000	-3.628601000	-0.472745000
H	-8.348468000	-3.567739000	1.300515000
C	-7.858624000	-0.990870000	-0.963099000
H	-7.215747000	-0.108957000	-1.049591000
H	-8.899968000	-0.649725000	-0.966089000
H	-7.700482000	-1.612018000	-1.851580000
C	-3.004402000	-0.749913000	-2.567027000
H	-3.495089000	-1.626099000	-2.163230000
C	-2.438338000	0.169213000	-1.678629000
C	-1.780213000	1.516941000	-3.555635000
C	-2.337495000	0.588922000	-4.426788000
C	-5.095997000	-1.249444000	0.294365000
H	-5.386055000	-0.205548000	0.320697000
C	-5.693099000	-3.555222000	0.253367000
C	-4.342174000	-3.887035000	0.207441000
C	-3.736216000	-1.565063000	0.246163000

C	-1.454645000	-3.928083000	-1.077832000
C	-0.179909000	-4.473022000	-1.157703000
H	0.149808000	-4.879027000	-2.109236000
C	0.186862000	-3.981507000	1.151941000
C	-1.091337000	-3.429228000	1.239245000
C	-0.030035000	2.073661000	-0.575236000
H	0.514310000	1.155554000	-0.767860000
C	0.486529000	2.984328000	0.344118000
H	1.397503000	2.712514000	0.862232000
C	-1.266264000	4.515891000	-0.196395000
H	-1.758885000	5.477650000	-0.080588000
C	-1.794653000	3.603651000	-1.100455000
H	-2.691899000	3.855555000	-1.658784000
C	-4.839240000	3.523419000	1.682688000
C	-4.319792000	1.978153000	-0.056692000
C	-4.014852000	2.822744000	2.549792000
C	-3.478235000	1.262504000	0.805129000
C	-2.796069000	-0.425131000	3.413643000
H	-3.606844000	-0.930917000	2.896661000
C	-2.045950000	-1.123677000	4.358666000
H	-2.290527000	-2.164653000	4.540996000
C	-0.766339000	0.849062000	4.797707000
C	-1.513391000	1.553316000	3.862564000
H	-1.291856000	2.599407000	3.668368000
C	2.759765000	-4.657720000	-1.440096000
H	3.776866000	-5.059063000	-1.486949000
H	2.820822000	-3.567279000	-1.451928000
H	2.240898000	-4.975716000	-2.349205000
C	2.944875000	-4.828396000	1.031511000
H	3.936210000	-5.270408000	0.888096000
H	2.541148000	-5.233149000	1.965629000
H	3.074576000	-3.748311000	1.150686000
C	0.995743000	6.421772000	0.764129000

H	1.801461000	6.094345000	0.098363000
H	0.222681000	6.896673000	0.151005000
H	1.401526000	7.179837000	1.444281000
C	1.550639000	4.646030000	2.418006000
H	1.213014000	3.766311000	2.977698000
H	2.418697000	4.354676000	1.817732000
H	1.888623000	5.395355000	3.141483000
C	-0.685834000	5.725379000	2.491651000
H	-1.502058000	6.202618000	1.941260000
H	-1.110232000	4.894643000	3.066453000
H	-0.288285000	6.460583000	3.200424000
C	-0.568195000	-2.712113000	6.239359000
H	-0.454719000	-3.257992000	5.295928000
H	0.071872000	-3.199124000	6.982171000
H	-1.605458000	-2.817561000	6.574571000
C	-0.316767000	-0.558153000	7.461262000
H	0.008496000	0.486337000	7.427860000
H	-1.361296000	-0.576916000	7.790594000
H	0.287658000	-1.073698000	8.215980000
C	-5.485695000	5.350012000	-0.639739000
H	-6.136554000	5.918958000	-1.312859000
H	-5.521705000	5.831313000	0.342441000
H	-4.459556000	5.425140000	-1.015410000
H	-1.297807000	2.407011000	-3.950393000
H	-2.278236000	0.773872000	-5.495497000
H	-5.355720000	4.405716000	2.050467000
H	-3.907550000	3.155204000	3.578691000
H	-4.408277000	1.644367000	-1.084427000
C	-5.943451000	3.885236000	-0.578943000
C	-7.377113000	3.819757000	-0.032798000
H	-7.735235000	2.784910000	0.005183000
H	-7.443266000	4.236721000	0.977150000
H	-8.054545000	4.390596000	-0.677769000

C	-5.935486000	3.318419000	-1.999608000
H	-4.935191000	3.347192000	-2.446264000
H	-6.293092000	2.283353000	-2.030751000
H	-6.599177000	3.915372000	-2.633324000
H	0.028145000	1.368226000	5.326912000
C	2.055201000	-5.154057000	-0.169735000
C	1.876872000	-6.677340000	-0.255644000
H	1.265640000	-6.956834000	-1.120349000
H	2.851333000	-7.169338000	-0.356095000
H	1.388800000	-7.068145000	0.644017000
H	-4.049891000	-4.932977000	0.172693000
H	-6.425054000	-4.355960000	0.253431000
H	-2.092981000	-3.921053000	-1.956939000
H	0.802983000	-3.993699000	2.044695000
H	-1.450334000	-3.051998000	2.193379000
C	1.316904000	-1.200113000	5.674202000
H	1.467095000	-1.722875000	4.723745000
H	1.679880000	-0.174030000	5.562196000
H	1.939115000	-1.694039000	6.428956000
P	-2.562618000	-0.163135000	0.120758000
C	1.325732000	-0.442560000	2.012104000
F	2.121637000	-1.447428000	2.057105000
F	2.698482000	0.647347000	0.252950000
F	1.811381000	0.494620000	2.738756000
B	3.742624000	0.406329000	-0.739681000
C	4.636975000	1.782756000	-0.768551000
C	4.007760000	2.990359000	-1.067561000
C	5.974928000	1.908010000	-0.410701000
C	4.624029000	4.229375000	-0.992798000
C	6.637274000	3.129944000	-0.328700000
C	5.956287000	4.300997000	-0.612803000
C	2.943772000	0.103805000	-2.140716000
C	1.885664000	-0.800020000	-2.123901000

C	3.159944000	0.711118000	-3.371564000
C	1.062404000	-1.057945000	-3.210284000
C	2.361374000	0.487565000	-4.487840000
C	1.299709000	-0.398980000	-4.406469000
C	4.593604000	-0.892557000	-0.225135000
C	5.046532000	-1.930033000	-1.029531000
C	4.908967000	-1.023663000	1.124903000
C	5.733123000	-3.035431000	-0.539227000
C	5.582410000	-2.111383000	1.659646000
C	5.993976000	-3.132931000	0.816258000
F	6.572986000	5.478927000	-0.532846000
F	3.954635000	5.349180000	-1.287068000
F	2.732708000	2.997402000	-1.481571000
F	6.729975000	0.836133000	-0.122412000
F	7.923346000	3.181114000	0.029065000
F	4.575719000	-0.053649000	1.992872000
F	5.834945000	-2.186778000	2.969623000
F	6.610223000	-4.207871000	1.307542000
F	6.108943000	-4.022810000	-1.358174000
F	4.828137000	-1.926212000	-2.354674000
F	1.613478000	-1.492094000	-1.001295000
F	0.051382000	-1.925937000	-3.118114000
F	0.522285000	-0.619248000	-5.466590000
F	2.609208000	1.118814000	-5.638801000
F	4.175926000	1.569713000	-3.551360000

5+ E(RPBE1PBE) = -3043.54988926

Au	1.393661000	0.121848000	-0.056155000
C	-3.557926000	2.414771000	2.057979000
C	-3.433549000	-3.075014000	0.996377000
C	-1.123746000	1.042047000	2.606997000
C	0.116834000	0.305461000	2.944874000
C	2.505001000	-1.102722000	3.557103000
C	0.095192000	2.516157000	-1.753476000

C	-1.099865000	1.789621000	-2.238816000
C	-3.404539000	0.472781000	-3.279226000
C	3.763616000	-1.898684000	3.899926000
C	2.429501000	3.854708000	-0.839346000
C	-4.884890000	3.123997000	1.801754000
C	-4.648723000	-0.261641000	-3.773126000
C	4.191476000	-2.686564000	-3.171045000
C	2.852440000	-2.662129000	-2.434509000
C	-0.935723000	-2.800974000	-0.353996000
C	0.356764000	-2.734014000	-1.071596000
C	-5.923786000	2.604310000	2.806441000
H	-6.884705000	3.106245000	2.648201000
H	-6.078693000	1.526182000	2.686818000
H	-5.614809000	2.788428000	3.840109000
C	-5.409712000	2.871500000	0.387229000
H	-4.717225000	3.238017000	-0.378622000
H	-5.591561000	1.806527000	0.204909000
H	-6.360837000	3.396408000	0.252705000
C	-4.696304000	4.635746000	1.992400000
H	-3.961338000	5.030975000	1.282705000
H	-5.645206000	5.157332000	1.825868000
H	-4.355080000	4.878052000	3.003717000
C	-4.309471000	-1.745120000	-3.984365000
H	-3.972416000	-2.223328000	-3.058723000
H	-3.518905000	-1.864082000	-4.733178000
H	-5.195217000	-2.286053000	-4.335554000
C	-5.168583000	0.313188000	-5.090308000
H	-6.060318000	-0.239758000	-5.402268000
H	-5.448557000	1.367481000	-4.991447000
H	-4.427245000	0.227380000	-5.892001000
C	-5.754856000	-0.138885000	-2.713810000
H	-5.451831000	-0.581678000	-1.759244000
H	-6.658073000	-0.659447000	-3.050677000

H	-6.013078000	0.910727000	-2.535581000
C	-2.931234000	1.634733000	1.088157000
H	-3.372320000	1.553141000	0.101697000
C	-1.734095000	0.954602000	1.343903000
C	-1.731457000	1.860825000	3.565672000
C	-2.920007000	2.527210000	3.300710000
C	-2.809817000	0.070517000	-2.079757000
H	-3.233909000	-0.770928000	-1.542708000
C	-2.807768000	1.534585000	-3.961237000
C	-1.680566000	2.171899000	-3.450496000
C	-1.681521000	0.700877000	-1.552529000
C	0.134542000	3.112737000	-0.485447000
C	1.280307000	3.762328000	-0.041577000
H	1.271435000	4.209987000	0.948309000
C	2.367847000	3.292048000	-2.117997000
C	1.225044000	2.635104000	-2.568703000
C	1.291192000	0.989478000	3.271364000
H	1.290989000	2.076466000	3.282306000
C	2.463415000	0.295419000	3.566866000
H	3.354668000	0.866582000	3.804217000
C	1.315759000	-1.779231000	3.249787000
H	1.295628000	-2.865519000	3.254530000
C	0.144468000	-1.095050000	2.949846000
H	-0.762280000	-1.650370000	2.729346000
C	-2.721731000	-4.194444000	0.544233000
C	-2.854628000	-1.826970000	0.782670000
C	-1.506092000	-4.058144000	-0.108900000
C	-1.628173000	-1.672600000	0.121782000
C	0.524178000	-1.968654000	-2.230915000
H	-0.316504000	-1.410893000	-2.634356000
C	1.750893000	-1.933017000	-2.894692000
H	1.830578000	-1.328474000	-3.791804000
C	2.670819000	-3.436446000	-1.278725000

C	1.453466000	-3.475453000	-0.611352000
H	1.348598000	-4.080846000	0.285075000
C	4.142561000	3.834134000	0.979155000
H	5.016119000	4.343890000	1.400756000
H	4.430766000	2.799257000	0.765177000
H	3.362622000	3.817712000	1.746682000
C	4.827838000	4.540086000	-1.302318000
H	5.697212000	5.043787000	-0.867433000
H	4.568143000	5.066444000	-2.226873000
H	5.130485000	3.519202000	-1.560969000
C	3.514450000	-2.672444000	5.203827000
H	3.292221000	-1.987240000	6.028925000
H	2.673758000	-3.366698000	5.105497000
H	4.402766000	-3.254503000	5.474018000
C	4.983148000	-0.996963000	4.095176000
H	5.203809000	-0.410812000	3.196526000
H	4.850943000	-0.304365000	4.933006000
H	5.861055000	-1.613557000	4.313726000
C	4.075012000	-2.896888000	2.774865000
H	3.256154000	-3.605469000	2.617825000
H	4.262330000	-2.380826000	1.827864000
H	4.969979000	-3.476279000	3.027330000
C	4.266821000	-1.614652000	-4.259904000
H	4.115568000	-0.610206000	-3.848066000
H	5.256215000	-1.638861000	-4.727934000
H	3.526976000	-1.776866000	-5.050395000
C	4.354211000	-4.068769000	-3.822850000
H	4.345771000	-4.865481000	-3.071761000
H	3.544505000	-4.265107000	-4.533726000
H	5.305275000	-4.123562000	-4.364686000
C	-4.609921000	-4.125056000	2.930776000
H	-5.573471000	-4.258782000	3.434799000
H	-4.228522000	-5.117186000	2.670353000

H	-3.913288000	-3.669898000	3.643204000
H	-1.268064000	1.951896000	4.544321000
H	-3.362910000	3.138211000	4.082343000
H	-3.124298000	-5.191932000	0.697813000
H	-0.984972000	-4.943430000	-0.462768000
H	-3.359514000	-0.940388000	1.146656000
C	-4.783291000	-3.238635000	1.689304000
C	-5.761752000	-3.908560000	0.713182000
H	-5.898293000	-3.297445000	-0.185883000
H	-5.407872000	-4.895565000	0.399180000
H	-6.739995000	-4.038077000	1.189175000
C	-5.373240000	-1.896616000	2.127273000
H	-4.719396000	-1.373068000	2.833621000
H	-5.556890000	-1.234208000	1.273957000
H	-6.332651000	-2.066995000	2.625862000
H	3.493300000	-4.030827000	-0.891295000
C	3.675974000	4.551725000	-0.296890000
C	3.333494000	6.010691000	0.039250000
H	2.547432000	6.078444000	0.797988000
H	4.219383000	6.525857000	0.426994000
H	2.990752000	6.547218000	-0.851954000
H	-1.244717000	3.004912000	-3.995215000
H	-3.219416000	1.883563000	-4.902216000
H	-0.742578000	3.079009000	0.153001000
H	3.224605000	3.348512000	-2.780516000
H	1.216869000	2.188543000	-3.559798000
C	5.352097000	-2.458178000	-2.192547000
H	5.282526000	-1.476990000	-1.713372000
H	5.388572000	-3.218368000	-1.406840000
H	6.304001000	-2.498348000	-2.733003000
P	-0.957719000	0.019184000	-0.017255000
C	3.357866000	0.267037000	-0.214680000
F	4.234008000	-0.080827000	0.653881000

F	3.974433000	0.735912000	-1.237616000

Al(C6F5)3 E(RPBE1PBE) = −2423.58817233

C	-0.61049300	1.85309100	-0.00457000
C	-1.80075200	2.23167200	0.60492400
C	0.12263100	2.86598800	-0.61139800
C	-2.25364000	3.54235600	0.61924500
C	-0.29091300	4.18969500	-0.62198300
C	-1.48822300	4.52295100	-0.00023800
C	-1.30033500	-1.45736100	-0.00419100
C	-1.02881900	-2.68194500	0.59457000
C	-2.54920300	-1.32464700	-0.59974000
C	-1.93800300	-3.72901700	0.60981800
C	-3.48991700	-2.34362300	-0.60804400
C	-3.17511900	-3.55156800	0.00248100
C	1.91136300	-0.39834000	-0.00206800
C	2.42428500	-1.53897000	-0.60811100
C	2.83241200	0.44497500	0.60789200
C	3.77775900	-1.84093200	-0.61666100
C	4.19431800	0.18367300	0.62428200
C	4.66311200	-0.96892700	0.00540300
F	-1.90266200	5.78311800	0.00122100
F	-3.39658600	3.87297000	1.21586800
F	-2.55363600	1.31245900	1.22438500
F	1.27474600	2.57460100	-1.23052400
F	0.43193900	5.13580100	-1.21692700
F	2.40986400	1.55686300	1.22495900

F	5.05032800	1.00918700	1.22210200
F	5.96195000	-1.23860500	0.00954800
F	4.23779800	-2.93996600	-1.21003200
F	1.59809300	-2.39170200	-1.22901600
F	0.14877000	-2.87983100	1.20235700
F	-1.64854000	-4.88839700	1.19615100
F	-4.05957000	-4.54013100	0.00538900
F	-4.67588600	-2.18588500	-1.19136900
F	-2.87732700	-0.17815300	-1.21074200
Al	0.00028400	-0.00118400	-0.00538700

F(Al(C6F5)3)$^-$ E(RPBE1PBE) = −2523.56459815

C	-1.87243100	-0.55920200	-0.62423600
C	-2.24575500	-1.81237300	-0.16512600
C	-2.90391100	0.36013800	-0.74164500
C	-3.55220600	-2.14514900	0.17290500
C	-4.22451700	0.08339400	-0.41819500
C	-4.54613900	-1.18588400	0.04357200
C	1.35433700	-1.29819500	-0.37243600
C	2.37126500	-1.91554800	-1.08172900
C	1.31903000	-1.58280900	0.98369400
C	3.29876000	-2.77061800	-0.49677000
C	2.21576800	-2.42851500	1.62004000
C	3.21568100	-3.02672100	0.86427100
C	0.47251400	1.77865600	-0.33285300

C	1.58670900	2.44490800	-0.81994700
C	-0.18169700	2.41033300	0.71369600
C	2.03260600	3.66624400	-0.33304500
C	0.21920100	3.63217600	1.24029200
C	1.33511000	4.26271500	0.70799700
F	0.07679700	0.05157500	-2.81217900
F	2.50652700	-1.70402500	-2.40256500
F	4.26471400	-3.34644800	-1.21904500
F	4.09439500	-3.84304200	1.44605100
F	2.13861600	-2.67425100	2.93129200
F	0.36642200	-1.01738900	1.75255900
F	-1.25697600	1.84281200	1.28936200
F	2.30616400	1.89620700	-1.81834600
F	-0.44444900	4.20486500	2.24905700
F	1.73846600	5.43432700	1.19890200
F	3.11163900	4.26940300	-0.84028900
F	-1.33162200	-2.79041800	-0.03097400
F	-3.86615000	-3.36769200	0.61223200
F	-5.80686900	-1.48205100	0.35855100
F	-5.18415200	1.00462000	-0.54461900
F	-2.64131600	1.60063500	-1.19623500
Al	-0.00121600	-0.01488000	-1.11743700

EDA-NOCV calculations (BP86-D3/def2-SVP)

4+ E = - -2884.487396

Au	-1.500820000	0.354197000	-0.102899000
C	1.365237000	-1.630386000	0.535853000
C	0.511062000	-2.588417000	1.137330000
C	0.977886000	-3.923615000	1.196174000
H	0.326213000	-4.687744000	1.648532000
C	2.244305000	-4.285352000	0.721439000
H	2.554922000	-5.339064000	0.796858000
C	3.124302000	-3.325700000	0.165050000
C	2.648875000	-2.007654000	0.078103000
H	3.278147000	-1.246577000	-0.399383000
C	-0.831961000	-2.261876000	1.683273000
C	-1.004719000	-1.281996000	2.686025000
H	-0.127539000	-0.733517000	3.066101000
C	-2.278465000	-1.010629000	3.216848000
H	-2.359306000	-0.250538000	4.006636000
C	-3.430180000	-1.694011000	2.764934000
C	-3.244229000	-2.663419000	1.749542000
H	-4.108843000	-3.229379000	1.369794000
C	-1.978702000	-2.947891000	1.221806000
H	-1.872014000	-3.693817000	0.419706000
C	4.521382000	-3.723076000	-0.344643000
C	5.288986000	-2.515068000	-0.926137000
H	5.453056000	-1.721842000	-0.166108000
H	4.762072000	-2.059853000	-1.791005000
H	6.287707000	-2.840838000	-1.280820000
C	5.340592000	-4.306379000	0.834784000
H	5.461376000	-3.555453000	1.643318000
H	6.352093000	-4.606410000	0.490110000
H	4.857665000	-5.203295000	1.273725000
C	4.364778000	-4.789887000	-1.458307000
H	3.770490000	-4.391225000	-2.306941000

H	3.859786000	-5.705730000	-1.088980000
H	5.359657000	-5.092040000	-1.846452000
C	-4.831493000	-1.472074000	3.370217000
C	-4.872541000	-0.247651000	4.308702000
H	-4.207440000	-0.374233000	5.187142000
H	-5.901600000	-0.102808000	4.695435000
H	-4.580445000	0.688231000	3.786108000
C	-5.880256000	-1.263932000	2.247816000
H	-5.658575000	-0.358722000	1.646107000
H	-6.890466000	-1.135640000	2.688481000
H	-5.933873000	-2.127711000	1.554809000
C	-5.203206000	-2.737184000	4.189058000
H	-5.234923000	-3.642163000	3.548267000
H	-6.202708000	-2.616566000	4.657134000
H	-4.463032000	-2.918122000	4.995383000
C	1.557885000	1.202719000	1.371314000
C	1.123760000	2.550022000	1.552479000
C	1.797183000	3.311101000	2.533463000
H	1.484889000	4.355653000	2.690535000
C	2.870393000	2.796531000	3.278524000
H	3.364290000	3.455381000	4.006343000
C	3.315250000	1.471869000	3.094056000
C	2.619869000	0.694111000	2.144958000
H	2.923360000	-0.352077000	1.999035000
C	0.013519000	3.188446000	0.790244000
C	-1.046810000	3.823073000	1.480147000
H	-1.056095000	3.812133000	2.581654000
C	-2.090841000	4.445716000	0.782983000
H	-2.894054000	4.926606000	1.363337000
C	-2.139440000	4.463701000	-0.633489000
C	-1.073063000	3.838826000	-1.314027000
H	-1.039787000	3.835664000	-2.412220000
C	-0.014931000	3.223956000	-0.622096000

H	0.814654000	2.787649000	-1.196869000
C	4.507379000	0.849294000	3.844495000
C	5.158482000	1.846876000	4.823670000
H	4.445280000	2.184724000	5.603786000
H	5.554047000	2.742142000	4.300574000
H	6.010915000	1.363569000	5.342851000
C	4.018890000	-0.386766000	4.643008000
H	4.866861000	-0.850968000	5.188450000
H	3.581629000	-1.164429000	3.983121000
H	3.247969000	-0.099239000	5.387557000
C	5.571576000	0.408536000	2.803813000
H	5.920706000	1.273223000	2.201744000
H	5.176167000	-0.356447000	2.102598000
H	6.452571000	-0.031897000	3.315563000
C	-3.314039000	5.149644000	-1.358648000
C	-3.382977000	6.636265000	-0.922628000
H	-4.215451000	7.152215000	-1.445027000
H	-2.440072000	7.165830000	-1.170166000
H	-3.556523000	6.744542000	0.167365000
C	-3.161517000	5.096644000	-2.892973000
H	-3.132431000	4.053482000	-3.272152000
H	-2.243487000	5.617377000	-3.235102000
H	-4.025305000	5.598269000	-3.374791000
C	-4.635433000	4.434339000	-0.975827000
H	-4.623740000	3.374530000	-1.306925000
H	-5.500165000	4.930428000	-1.463710000
H	-4.817984000	4.449163000	0.118437000
C	1.735135000	0.398647000	-1.438521000
C	1.283972000	-0.196081000	-2.643200000
C	2.068977000	0.004475000	-3.798371000
H	1.735989000	-0.440808000	-4.749138000
C	3.269149000	0.729157000	-3.747718000
H	3.856301000	0.838233000	-4.672796000

C	3.741125000	1.301369000	-2.542804000
C	2.937319000	1.135194000	-1.398715000
H	3.253351000	1.566136000	-0.438894000
C	0.052651000	-1.044735000	-2.690557000
C	-1.188598000	-0.513102000	-3.117096000
H	-1.240796000	0.539391000	-3.438897000
C	-2.340157000	-1.316853000	-3.144345000
H	-3.283476000	-0.869885000	-3.495158000
C	-2.312767000	-2.671892000	-2.737377000
C	-1.068941000	-3.188783000	-2.316846000
H	-0.981228000	-4.237389000	-2.000577000
C	0.092070000	-2.398271000	-2.298371000
H	1.043709000	-2.840210000	-1.966643000
C	5.094872000	2.033767000	-2.492744000
C	5.389938000	2.605784000	-1.089658000
H	4.625284000	3.345574000	-0.773500000
H	6.370546000	3.123296000	-1.094517000
H	5.441146000	1.809407000	-0.317593000
C	5.090600000	3.200484000	-3.512329000
H	4.932713000	2.846370000	-4.551361000
H	6.062941000	3.735010000	-3.488462000
H	4.290266000	3.931955000	-3.275963000
C	6.211796000	1.020357000	-2.855636000
H	6.224044000	0.171763000	-2.140033000
H	7.205720000	1.513528000	-2.823384000
H	6.077664000	0.602928000	-3.874357000
C	-3.595894000	-3.524885000	-2.799322000
C	-4.710751000	-2.851023000	-1.960566000
H	-5.637585000	-3.460895000	-1.985927000
H	-4.970807000	-1.843321000	-2.344102000
H	-4.396991000	-2.749454000	-0.900597000
C	-3.377371000	-4.950125000	-2.249305000
H	-2.619819000	-5.511200000	-2.833756000

H	-4.325228000	-5.523144000	-2.304150000
H	-3.058626000	-4.940031000	-1.185552000
C	-4.052045000	-3.631889000	-4.277569000
H	-3.267055000	-4.109169000	-4.899407000
H	-4.271853000	-2.636609000	-4.714998000
H	-4.974258000	-4.245149000	-4.355023000
C	-3.721625000	0.434297000	-0.519897000
H	-3.811380000	0.900601000	-1.515851000
H	-4.051559000	-0.613949000	-0.429814000
C	-3.465811000	1.201048000	0.605941000
H	-3.578859000	0.772305000	1.615920000
H	-3.324046000	2.292968000	0.537000000
P	0.813025000	0.076921000	0.121241000

A E = -1249.714923

Au	1.872230000	-0.032543000	0.008212000
C	4.070613000	-0.732862000	0.136282000
C	4.083990000	0.626690000	-0.110366000
H	4.193241000	1.020801000	-1.136369000
H	4.191513000	1.356908000	0.711421000
H	4.168852000	-1.464116000	-0.685716000
H	4.166958000	-1.129553000	1.162666000
P	-0.463317000	-0.004824000	0.000235000
C	-1.085428000	1.667997000	-0.391353000
C	-2.135338000	2.245965000	0.353672000
C	-0.525644000	2.356084000	-1.492431000
C	-2.623201000	3.513257000	-0.009997000
C	-1.023087000	3.618411000	-1.848839000
C	-2.070362000	4.197351000	-1.106909000
H	-2.569952000	1.711023000	1.212452000
H	0.293036000	1.900747000	-2.074573000
H	-3.441680000	3.967389000	0.569544000
H	-0.590146000	4.155332000	-2.706888000
H	-2.455550000	5.190579000	-1.385116000

C	-1.152114000	-1.149090000	-1.244605000
C	-2.245968000	-0.769531000	-2.051701000
C	-0.603774000	-2.447805000	-1.347898000
C	-2.787932000	-1.695313000	-2.959990000
C	-1.154905000	-3.365350000	-2.254856000
C	-2.245739000	-2.988654000	-3.061379000
H	-2.671091000	0.243309000	-1.973565000
H	0.250141000	-2.742432000	-0.714830000
H	-3.639652000	-1.401728000	-3.592770000
H	-0.729582000	-4.377453000	-2.336713000
H	-2.673188000	-3.708532000	-3.776480000
C	-1.149089000	-0.479436000	1.623999000
C	-0.566627000	0.062144000	2.792669000
C	-2.275577000	-1.325019000	1.713784000
C	-1.118313000	-0.237291000	4.047431000
C	-2.816957000	-1.622755000	2.976201000
C	-2.242179000	-1.080315000	4.139140000
H	0.314240000	0.721980000	2.720312000
H	-2.726801000	-1.749923000	0.803631000
H	-0.667191000	0.184767000	4.958641000
H	-3.694044000	-2.284065000	3.049579000
H	-2.669709000	-1.318685000	5.125383000

B E = -1058.903983

Au	0.000000000	0.000000000	1.256743000
C	0.000000000	0.000000000	-0.765308000
C	0.000000000	0.688575000	-2.919625000
C	0.000000000	-0.688575000	-2.919625000
C	-2.540586000	2.329506000	-1.110016000
C	-2.540586000	-2.329506000	-1.110016000
C	2.540586000	-2.329506000	-1.110016000
C	2.540586000	2.329506000	-1.110016000
C	0.000000000	2.451103000	-1.105695000
C	1.247698000	3.069910000	-0.862412000

C	-1.247698000	3.069910000	-0.862412000
C	1.220262000	4.387788000	-0.363692000
C	-1.220262000	4.387788000	-0.363692000
C	0.000000000	5.039110000	-0.120532000
C	0.000000000	-2.451103000	-1.105695000
C	1.247698000	-3.069910000	-0.862412000
C	-1.247698000	-3.069910000	-0.862412000
C	1.220262000	-4.387788000	-0.363692000
C	-1.220262000	-4.387788000	-0.363692000
C	0.000000000	-5.039110000	-0.120532000
N	0.000000000	1.087122000	-1.589276000
N	0.000000000	-1.087122000	-1.589276000
H	0.000000000	1.413865000	-3.739708000
H	0.000000000	-1.413865000	-3.739708000
H	2.171207000	-4.906668000	-0.165419000
H	-2.171207000	-4.906668000	-0.165419000
H	0.000000000	-6.070217000	0.265475000
H	2.171207000	4.906668000	-0.165419000
H	-2.171207000	4.906668000	-0.165419000
H	0.000000000	6.070217000	0.265475000
H	2.613264000	1.425088000	-0.467032000
H	2.619847000	1.979293000	-2.160867000
H	3.418076000	2.969110000	-0.898143000
H	2.619847000	-1.979293000	-2.160867000
H	2.613264000	-1.425088000	-0.467032000
H	3.418076000	-2.969110000	-0.898143000
H	-3.418076000	-2.969110000	-0.898143000
H	-2.613264000	-1.425088000	-0.467032000
H	-2.619847000	-1.979293000	-2.160867000
H	-2.619847000	1.979293000	-2.160867000
H	-2.613264000	1.425088000	-0.467032000
H	-3.418076000	2.969110000	-0.898143000
C	0.692776000	0.000000000	3.414226000

C	-0.692776000	0.000000000	3.414226000
H	-1.263207000	0.938593000	3.529915000
H	-1.263207000	-0.938593000	3.529915000
H	1.263207000	0.938593000	3.529915000
H	1.263207000	-0.938593000	3.529915000

6+ E = -2919.238458

Au	1.478249000	-0.014073000	-0.122927000
C	-3.271051000	-2.103309000	2.646746000
C	-3.475565000	-1.180719000	-2.993302000
C	-0.905171000	-2.647275000	1.093901000
C	0.287917000	-2.968293000	0.262075000
C	2.629896000	-3.336532000	-1.354489000
C	0.407375000	1.697317000	2.367158000
C	-0.838649000	2.247583000	1.764511000
C	-3.297397000	3.285623000	0.681208000
C	3.869505000	-3.461567000	-2.261043000
C	2.826432000	0.512576000	3.359502000
C	-4.570276000	-1.854706000	3.434641000
C	-4.624200000	3.775986000	0.073247000
C	3.661052000	3.779534000	-1.844778000
C	2.375621000	2.987520000	-2.155132000
C	-1.152500000	0.505965000	-2.764250000
C	0.036539000	1.398234000	-2.640388000
C	-5.647410000	-2.847136000	2.923781000
H	-6.603140000	-2.691784000	3.466506000
H	-5.837834000	-2.701554000	1.839801000
H	-5.342821000	-3.902735000	3.076049000
C	-5.097314000	-0.415791000	3.244004000
H	-4.367609000	0.344485000	3.593061000
H	-5.344863000	-0.196880000	2.184056000
H	-6.026656000	-0.277142000	3.832703000
C	-4.313229000	-2.089027000	4.944744000
H	-3.539018000	-1.393211000	5.329719000

H	-5.245201000	-1.919978000	5.523058000
H	-3.976789000	-3.124338000	5.156378000
C	-4.430903000	3.968113000	-1.453932000
H	-4.137303000	3.024328000	-1.958654000
H	-3.644827000	4.723515000	-1.662025000
H	-5.374853000	4.317870000	-1.921380000
C	-5.084747000	5.113436000	0.687567000
H	-6.044374000	5.427556000	0.229209000
H	-5.251065000	5.030549000	1.781735000
H	-4.351905000	5.926846000	0.506245000
C	-5.719357000	2.707588000	0.330256000
H	-5.469770000	1.734241000	-0.141365000
H	-6.689846000	3.041566000	-0.092102000
H	-5.858275000	2.533191000	1.417427000
C	-2.708022000	-1.129653000	1.801356000
H	-3.171714000	-0.136321000	1.737910000
C	-1.547436000	-1.381289000	1.036111000
C	-1.443086000	-3.606120000	1.980774000
C	-2.595991000	-3.344265000	2.732572000
C	-2.757907000	2.057876000	0.243281000
H	-3.278304000	1.491264000	-0.543832000
C	-2.558739000	4.000339000	1.648067000
C	-1.358260000	3.491353000	2.174612000
C	-1.553166000	1.543481000	0.755489000
C	0.379934000	0.480061000	3.088505000
C	1.564398000	-0.095442000	3.564947000
H	1.497536000	-1.047879000	4.112645000
C	2.839495000	1.749819000	2.676930000
C	1.655374000	2.333843000	2.188006000
C	1.541852000	-3.239789000	0.851098000
H	1.631546000	-3.258949000	1.948486000
C	2.688199000	-3.421103000	0.054681000
H	3.646674000	-3.606029000	0.559805000

C	1.356151000	-3.122944000	-1.934505000
H	1.251532000	-3.081448000	-3.029535000
C	0.208242000	-2.948289000	-1.150961000
H	-0.762687000	-2.775468000	-1.638866000
C	-2.903530000	-0.546529000	-4.121829000
C	-2.840484000	-0.973700000	-1.753990000
C	-1.771407000	0.273237000	-4.010955000
C	-1.698058000	-0.153681000	-1.631332000
C	-0.047395000	2.625677000	-1.949572000
H	-1.022338000	2.976406000	-1.581177000
C	1.101982000	3.394772000	-1.703675000
H	0.984347000	4.330694000	-1.140593000
C	2.440966000	1.786736000	-2.903711000
C	1.301441000	1.000450000	-3.137970000
H	1.393286000	0.046641000	-3.680920000
C	4.143517000	-1.644240000	3.419723000
H	5.075173000	-2.133940000	3.771046000
H	4.116638000	-1.714471000	2.313389000
H	3.293374000	-2.236631000	3.815105000
C	5.385120000	0.539172000	3.416058000
H	6.276361000	0.002237000	3.799673000
H	5.448484000	1.582427000	3.787932000
H	5.459900000	0.559259000	2.309283000
C	3.719108000	-4.724966000	-3.147575000
H	3.642943000	-5.638518000	-2.522876000
H	2.814383000	-4.675885000	-3.787481000
H	4.599020000	-4.836130000	-3.815190000
C	5.176426000	-3.582769000	-1.449659000
H	5.345755000	-2.702343000	-0.796184000
H	5.186690000	-4.492949000	-0.815407000
H	6.042073000	-3.654389000	-2.139048000
C	3.964242000	-2.204571000	-3.167117000
H	3.086069000	-2.106378000	-3.837295000

H	4.029290000	-1.274335000	-2.565102000
H	4.867217000	-2.258441000	-3.809975000
C	3.370971000	5.080219000	-1.067279000
H	2.893691000	4.878321000	-0.085158000
H	4.319756000	5.618435000	-0.867115000
H	2.713710000	5.767310000	-1.638822000
C	4.379413000	4.145142000	-3.168161000
H	4.675303000	3.245487000	-3.745176000
H	3.724965000	4.764005000	-3.815661000
H	5.302996000	4.723982000	-2.958401000
C	-4.421879000	-3.232746000	-4.101203000
H	-5.314764000	-3.881522000	-4.217830000
H	-4.136607000	-2.874279000	-5.111233000
H	-3.590757000	-3.858201000	-3.714052000
H	-0.952647000	-4.589713000	2.053500000
H	-2.984103000	-4.135387000	3.392590000
H	-3.353566000	-0.688404000	-5.116631000
H	-1.358518000	0.764054000	-4.906360000
H	-3.225226000	-1.473514000	-0.854942000
C	-4.732001000	-2.061059000	-3.134629000
C	-5.886801000	-1.199033000	-3.706427000
H	-6.121526000	-0.350985000	-3.029867000
H	-5.640521000	-0.780328000	-4.703485000
H	-6.804610000	-1.812693000	-3.820250000
C	-5.182977000	-2.647959000	-1.779665000
H	-4.406377000	-3.298852000	-1.326570000
H	-5.440515000	-1.853826000	-1.047417000
H	-6.089952000	-3.269100000	-1.924442000
H	3.408469000	1.432666000	-3.293311000
C	4.100602000	-0.168370000	3.896083000
C	4.062095000	-0.130727000	5.446254000
H	3.171370000	-0.657437000	5.845944000
H	4.964333000	-0.621019000	5.868206000

H	4.032192000	0.914955000	5.815417000
H	-0.815794000	4.061353000	2.945441000
H	-2.918552000	4.971712000	2.015571000
H	-0.581036000	-0.026582000	3.263291000
H	3.789214000	2.274609000	2.499796000
H	1.702956000	3.275892000	1.619111000
C	4.587173000	2.892665000	-0.974212000
H	4.086072000	2.619058000	-0.021335000
H	4.875049000	1.957933000	-1.496939000
H	5.523192000	3.433830000	-0.722720000
P	-0.887233000	-0.002024000	0.012545000
C	3.424624000	-0.198429000	-0.052933000
O	4.553381000	-0.364787000	0.046297000

5. References

1 M. Navarro, J. Miranda-Pizarro, J. J. Moreno, C. Navarro-Gilabert, I. Fernández, J. Campos, *Chem. Commun* **2021**, *57*, 9280.

2 G. M. Sheldrick, Crystal structure refinement with SHELXL. *Acta Cryst.* **2008**, *A64*, 112.

3 O. V. Dolomanov, L. J. Bourhis, R. J. Gildea, J. A. K. Howard, H. J. Puschmann, *Appl. Cryst.* **2009**, *42*, 339.

4 M. J. Frisch, G. W. Trucks, H. B. Schlegel, G. E. Scuseria, M. A. Robb, J. R. Cheeseman, G. Scalmani, V. Barone, B. Mennucci, G. A. Petersson, H. Nakatsuji, M. Caricato, X. Li, H. P. Hratchian, A. F. Izmaylov, J. Bloino, G. Zheng, J. L. Sonnenberg, M. Hada, M. Ehara, K. Toyota, R. Fukuda, J. Hasegawa, M. Ishida, T. Nakajima, Y. Honda, O. Kitao, H. Nakai, T. Vreven, J. A. J. Montgomery, J. E. Peralta, F. Ogliaro, M. Bearpark, J. J. Heyd, E. Brothers, K. N. Kudin, V. N. Staroverov, R. Kobayashi, J. Normand, K. Raghavachari, A. Rendell, J. C. Burant, S. S. Iyengar, J. Tomasi, M. Cossi, N. Rega, J. M. Millam, M. Klene, J. E. Knox, J. B. Cross, V. Bakken, C. Adamo, J. Jaramillo, R. Gomperts, R. E. Stratmann, O. Yazyev, A. J. Austin, R. Cammi, C. Pomelli, J. W. Ochterski, R. L. Martin, K. Morokuma, V. G. Zakrzewski, G. A. Voth, P. Salvador, J. J. Dannenberg, S. Dapprich, A. D. Daniels, O. Farkas, J. B. Foresman, J. V. Ortiz, J. Cioslowski, D. J. Fox, Gaussian 09, Revision D.01, Gaussian, Inc.: Wallingford CT, **2013**.

5 J. P. Perdew, K. Burke, M. Ernzerhof, *Phys. Rev. Lett.* **1996**, *77*, 3865.

6 S. Grimme, J. Antony, S. Ehrlich, H. Krieg, *J. Chem. Phys*. **2010**, *132*, 154104.

7 a) W. J. Hehre, R. Ditchfield, J A. Pople. *J. Phys. Chem.* **1972**, *56*, 2257; b) P. C. Hariharan, J. A. Pople. *Theor. Chim. Acta.* **1973**, *28*, 213.; c) M. M. Francl, W. J. Pietro, W. J. Hehre, J. S. Binkley, M. S. Gordon, D. J. Defrees, J. A. Pople. *J. Chem. Phys.* **1982**, *77*, 3654.

8 D. Andrae, U. Haeussermann, M. Dolg, H. Stoll, H. Preuss. *Theor. Chim. Acta* **1990**, *77*, 123.

9 A. V. Marenich, C. J. Cramer, D. G. Truhlar, *J. Phys. Chem. B* **2009**, *113*, 6378.

10 (a) A. D. Becke, *Phys. Rev. A* **1988**, *38*, 3098. (b) J. P. Perdew, *Phys. Rev. B* **1986**, *33*, 8822.

11 S. Grimme, J. Antony, S. Ehrlich, H. Krieg, *J. Chem. Phys*. **2010**, *132*, 154104.

12 F. Weigend, R. Ahlrichs, *Phys. Chem. Chem. Phys*. **2005**, *7*, 3297.

13 M. P. Mitoraj, A. Michalak, T. Ziegler, *J. Chem. Theory Comput*. **2009**, *5*, 962.

14 For a review, see: M. von Hopffgarten, G. Frenking, *WIREs Comput. Mol. Sci*. **2012**, *2*, 43.

15 M. P. Mitoraj, A. Michalak, *J. Mol. Model*. **2007**, *13*, 347.

16 See, for instance: (a) M. P. Mitoraj, A. Michalak, T. Ziegler, *Organometallics* **2009**, *28*, 3727. (b) A. N. N. Thi, G. Frenking, *Chem. Eur. J.* **2012**, *18*, 12733. (c) M. Parafiniuk, M. P. Mitoraj, *Organometallics* **2013**, *32*, 4103.

17 ADF program: www.scm.com.

18 J. G. Snijders, E. J. Baerends,P. Vernoojs, P. *At. Data. Nucl. Data Tables* **1982**, *26*, 483.

19 A. Krijn, E. J. Baerends, E. J.; Fit Functions in the HFS-Method, Internal Report (in Dutch), Vrije Universiteit Amsterdam, The Netherlands, **1984**.

20 (a) E. van Lenthe, E. J. Baerends, J. G. Snijders, *J. Chem. Phys.* **1993**, *99*, 4597. (b) E. van Lenthe, E. J. Baerends, J. G. Snijders, *J. Chem. Phys.* **1994**, *101*, 9783. (c) E. van Lenthe, A. Ehlers, E. J. Baerends, *J. Chem. Phys.* **1999**, *110*, 8943.
